# Intracellular BAPTA directly inhibits PFKFB3, thereby impeding mTORC1-driven *Mcl-1* translation and killing MCL-1-addicted cancer cells

**DOI:** 10.1038/s41419-023-06120-4

**Published:** 2023-09-08

**Authors:** Flore Sneyers, Martijn Kerkhofs, Femke Speelman-Rooms, Kirsten Welkenhuyzen, Rita La Rovere, Ahmed Shemy, Arnout Voet, Guy Eelen, Mieke Dewerchin, Stephen W. G. Tait, Bart Ghesquière, Martin D. Bootman, Geert Bultynck

**Affiliations:** 1 KU Leuven, Laboratory of Molecular and Cellular Signaling, Department of Cellular and Molecular Medicine, Campus Gasthuisberg O&N I, Herestraat 49 box 802, 3000 Leuven, Belgium; 2 KU Leuven, Laboratory of Chemical Biology, Department of Cellular and Molecular Medicine, Campus Gasthuisberg O&N I bis, Herestraat 49 box 901, 3000 Leuven, Belgium; 3 KU Leuven, Laboratory for Biomolecular Modelling and Design, Department of Chemistry, Celestijnenlaan 200G, 3001 Heverlee, Belgium; 4 KU Leuven, Laboratory of Angiogenesis and Vascular Metabolism, Department of Oncology, Leuven Cancer Institute, Campus Gasthuisberg O&N4, Herestraat 49 box 912, Leuven, Belgium; 5 VIB–KU Leuven, Center for Cancer Biology, Laboratory of Angiogenesis and Vascular Metabolism, Campus Gasthuisberg O&N4, Herestraat 49 box 912, 3000 Leuven, Belgium; 6 Cancer Research UK Beatson Institute, School of Cancer Sciences, University of Glasgow, Glasgow, UK; 7 KU Leuven, Laboratory of Applied Mass Spectrometry, Department of Cellular and Molecular Medicine, Leuven, Belgium — VIB, Metabolomics Core Facility Leuven, Center for Cancer Biology, Leuven, Belgium, Herestraat 49 box 912, 3000 Leuven, Belgium; 8 School of Life, Health and Chemical Sciences, Faculty of Science, Technology, Engineering and Mathematics, The Open University, Walton Hall, Milton Keynes, MK7 6AA UK

**Keywords:** Target identification, Apoptosis, Lymphoma, Metabolomics, Calcium signalling

## Abstract

Intracellular Ca^2+^ signals control several physiological and pathophysiological processes. The main tool to chelate intracellular Ca^2+^ is intracellular BAPTA (BAPTA_i_), usually introduced into cells as a membrane-permeant acetoxymethyl ester (BAPTA-AM). Previously, we demonstrated that BAPTA_i_ enhanced apoptosis induced by venetoclax, a BCL-2 antagonist, in diffuse large B-cell lymphoma (DLBCL). This finding implied a novel interplay between intracellular Ca^2+^ signaling and anti-apoptotic BCL-2 function. Hence, we set out to identify the underlying mechanisms by which BAPTA_i_ enhances cell death in B-cell cancers. In this study, we discovered that BAPTA_i_ alone induced apoptosis in hematological cancer cell lines that were highly sensitive to S63845, an MCL-1 antagonist. BAPTA_i_ provoked a rapid decline in MCL-1-protein levels by inhibiting mTORC1-driven *Mcl-1* translation. These events were not a consequence of cell death, as BAX/BAK-deficient cancer cells exhibited similar downregulation of mTORC1 activity and MCL-1-protein levels. Next, we investigated how BAPTA_i_ diminished mTORC1 activity and identified its ability to impair glycolysis by directly inhibiting 6-phosphofructo-2-kinase/fructose-2,6-bisphosphatase 3 (PFKFB3) activity, a previously unknown effect of BAPTA_i_. Notably, these effects were also induced by a BAPTA_i_ analog with low affinity for Ca^2+^. Consequently, our findings uncover PFKFB3 inhibition as an Ca^2+^-independent mechanism through which BAPTA_i_ impairs cellular metabolism and ultimately compromises the survival of MCL-1-dependent cancer cells. These findings hold two important implications. Firstly, the direct inhibition of PFKFB3 emerges as a key regulator of mTORC1 activity and a promising target in MCL-1-dependent cancers. Secondly, cellular effects caused by BAPTA_i_ are not necessarily related to Ca^2+^ signaling. Our data support the need for a reassessment of the role of Ca^2+^ in cellular processes when findings were based on the use of BAPTA_i_.

## Introduction

Intracellular Ca^2+^ is a ubiquitous second messenger that controls cellular processes ranging from fertilization and cell division to cell death [1, 2].

Cancer cells attenuate intracellular Ca^2+^ signaling, thereby promoting tumorigenesis and conferring resistance to chemotherapeutic regimens [3, 4]. Several tumor suppressor proteins and (proto-)oncogenes, including anti-apoptotic members of the BCL-2 family, directly modulate Ca^2+^ fluxes to enhance survival [5–8].

Moreover, anti-apoptotic BCL-2-family members, including BCL-2, BCL-XL and MCL-1, inhibit the mitochondrial apoptotic pathway [9]. In healthy cells, cell stress including oncogenic stress results in the activation of BAX/BAK and subsequent BAX/BAK-pore formation [10]. These BAX/BAK pores mediate mitochondrial outer membrane permeabilization, resulting in the release of cytochrome *c*, SMAC/DIABLO release, OMI and other proteins [11]. In the cytosol, cytochrome *c* forms a complex with apoptotic peptidase activating factor 1 (APAF1), called the apoptosome, while SMAC/DIABLO and OMI block caspase inhibitor XIAP [12]. These events activate caspase 9 and the executioner caspases, caspase 3/7, and induction of apoptosis. Increased abundance of anti-apoptotic BCL-2, BCL-XL or MCL-1 is found in several B-cell malignancies, including diffuse large B-cell lymphoma (DLBCL), thereby counteracting pro-apoptotic signaling induced by oncogenic stress [13]. This “primed to death” status represents an Achilles’ heel in cancers that can be exploited by BH3-mimetic drugs (such as venetoclax), which antagonize anti-apoptotic BCL-2 proteins. However, venetoclax resistance in cancer cells has already emerged due to acquired mutations in BCL-2 and upregulation of BCL-XL or MCL-1 [14]. Selective BH3-mimetic antagonists have been developed for BCL-XL (including A-1331852, A-1155463 and WEHI-539) and MCL-1 (S63845), thereby driving cell death in BCL-XL- and MCL-1-dependent cancer cells [15, 16].

We previously demonstrated that intracellular BAPTA (BAPTA_i_), a widely used, high-affinity chelator of cytosolic Ca^2+^ loaded into cells as BAPTA-acetoxymethyl ester (BAPTA-AM), enhanced venetoclax-induced cell death in OCI-LY-1 and SU-DHL-4 cells, two DLBCL cell lines [17]. Extracellularly added BAPTA-AM can pass across the plasma membrane and thus enter the cell, where it is hydrolyzed into the free acid form of BAPTA that chelates cytosolic Ca^2+^. As such, BAPTA is trapped in the cells. BAPTA-AM is typically applied in the extracellular environment at about 10 μM. However, due to the accumulation of the hydrolyzed free acid form, which is cell membrane-impermeant, intracellular BAPTA concentrations can reach 1 to 2 mM [15].

Given the fast Ca^2+^-chelating properties enabling BAPTA_i_ to buffer Ca^2+^ in close proximity to Ca^2+^ channels and microdomains, our results suggested an unprecedented interplay between constitutive Ca^2+^ signaling and the anti-apoptotic function of BCL-2 proteins in B-cell cancer cells. However, intracellularly loaded BAPTA, and its derivatives, may also interfere with cell physiological processes independently of its Ca^2+^-chelating properties by directly targeting proteins such as the Na^+^/K^+^ ATPase [18, 19].

Hence, we aimed to uncover the molecular mechanisms by which BAPTA_i_ impacted cancer cell survival. BAPTA_i_ alone induced cell death especially in MCL-1-dependent hematological cancer cells, but not in those cells not addicted to MCL-1. To distinguish between Ca^2+^-dependent versus Ca^2+^-independent effects of BAPTA_i_, we used various BAPTA derivatives (Supplementary Table 1), including tetrafluoro-BAPTA_i_ (TF-BAPTA_i_) with 400-fold lower affinity for Ca^2+^ (*K*_D_ ~ 65 µM) compared to BAPTA (*K*_D_ ~ 160 nM). Thus, the *K*_D_ of TF-BAPTA_i_ for Ca^2+^ is about 600-fold higher than the basal cytosolic Ca^2+^ concentration of 100 nM. Despite these differences, BAPTA_i_ and TF-BAPTA_i_ were equally potent in inducing the death of MCL-1-addicted cancer cells. Employing a diverse range of methodologies, we unveiled a novel Ca^2+^-independent effect of BAPTA_i_, involving the inhibition of glycolysis, thereby swiftly suppressing mTORC1 activity and consequently abrogating mTORC1-driven processes such as *Mcl-1* translation. Due to its short-lived nature, MCL-1-protein levels rapidly decline in BAPTA_i_-loaded cells. In cancer cells or genetically engineered cells dependent on MCL-1 for cell survival, BAPTA_i_ (and TF-BAPTA_i_) induced significant cell death, regardless of intracellular Ca^2+^ buffering. We further identified the direct target of BAPTA_i_ as 6-phosphofructo-2-kinase/fructose-2,6-bisphosphatase-3 (PFKFB3), demonstrating that BAPTA_i_ directly inhibited recombinantly produced and purified PFKFB3 enzyme. In addition, direct PFKFB3 inhibition using AZ PFKFB3 67 mimicked the inhibitory effect of BAPTA_i_ on mTORC1 activity and MCL-1-protein levels. Collectively, our findings shed new light on the cellular effects and direct targets of BAPTA_i_, establishing a novel link between PFKFB3, mTORC1 and MCL-1, independently of Ca^2+^ chelation.

## Materials and methods

### Cell culture and transfections

SU-DHL-4 and OCI-LY-1 DLBCL cell lines were kindly provided by Dr. Anthony Letai (Dana-Farber Cancer Institute, Boston, Massachusetts, USA). OCI-AML-2 and OCI-AML-3 acute myeloid leukemia cell lines were previously obtained from Dr. Jean-Emmanuel Sarry (Université de Toulouse, France). H929 multiple myeloma cell line was purchased from DSMZ (Braunschweig, Germany). WEHI-231 B lymphocyte cell line (CRL-1702™) was purchased from ATCC. OCI-LY-1 cells were cultured in suspension in Iscove modified Dulbecco’s medium (IMDM, Life Technologies, Brussels, Belgium). SU-DHL-4 and H929 cells were cultured in suspension in Roswell Park Memorial Institute (RPMI-1640) medium (Life Technologies, Brussels, Belgium). OCI-AML-2 and OCI-AML3 were cultured in MEM alpha medium (Life Technologies, Brussels, Belgium). Mito-primed seminal vesicle epithelial cells (SVECs) were kindly obtained from Prof. Stephen Tait [20]. All media were supplemented with 10% fetal bovine serum (FBS, Life Technologies), 2% GlutaMAX^TM^ Supplement (Life Technologies, Brussels, Belgium) and 2% penicillin/streptomycin (Life Technologies, Brussels, Belgium). Cultures were incubated at 37 °C and 5% CO_2,_ and sterile conditions were always maintained. Cells were validated through STR profiling and were cultured in mycoplasma-free conditions, whereby cell cultures were monitored once every two weeks for mycoplasma infection. Research with human cell lines was approved by ethical committee UZ Leuven (S63808).

### Transfection

Twenty-four hours after seeding, OCI-LY-1 cells were transfected using the Amaxa® Cell Line Nucleofector® Kit L (Lonza, Basel, Switzerland), program C-05 as previously described [21]. Cells were briefly transfected with the constructs described below and collected at 18 h posttransfection to use in experiments and to confirm transfection via western blot.

### Reagents, antibodies and constructs

The following reagents were used in this study. EGTA (Acros Organics, Geel, Belgium, 409910250), dimethyl sulfoxide (DMSO, Sigma-Aldrich, Overijse, Belgium), Fura-2-AM (Life Technologies, Carlsbad, CA, USA, F1221), 2-DG (Sigma-Aldrich, Overijse, Belgium; purity ≥98%), 1,2-Bis-(o-Aminophenoxy)-ethane-N,N,N’,N’-tetraacetic acid, tetraacetoxymethyl ester (BAPTA-AM, Life Technologies, Brussels, Belgium), 5,5′,6,6′-Tetrafluoro-BAPTA-AM (Interchim, Montluçon, France), 5,5′-difluoro-BAPTA-AM and 5,5′-dimethyl-BAPTA-AM (Sigma-Aldrich, Overijse, Belgium), S63845 (Gentaur, Kampenhout, Belgium), venetoclax (ABT-199, Active Biochem, Kowloon, Hong Kong), A1155463 (Selleck Chemicals, Houston, USA), Z-Val-Ala-DL-Asp(OMe)-fluoromethylketone (ZVAD-(OMe)-FMK, ABCAM, Cambridge, UK) cycloheximide (C7698, Sigma-Aldrich, Overijse, Belgium), AZ PFKFB3 67 (Bio-techne, Abingdon, UK).

In addition to BAPTA itself, three different BAPTA analogs were used in this study: tetrafluoro-BAPTA (TF-BAPTA), difluoro-BAPTA (DF-BAPTA) and dimethyl-BAPTA (DM-BAPTA) (Supplementary Table 1). The value of EGTA reported is obtained at pH 7.4 and 20 °C. Whereas placing fluor groups on the benzene ring of BAPTA (para- position, or meta- and para- positions, for DF-BAPTA and TF-BAPTA, respectively) severely reduces the affinity for binding Ca^2+^, methyl groups (para position) augment the Ca^2+^-chelating properties of BAPTA. All Ca^2+^ chelators used in this study were introduced into the cells as acetoxymethyl esters using standard loading procedures.

The following primary antibodies were used in this study: anti-MCL-1 (4572, Cell Signaling Technology), anti-BCL-XL (MA5-15142, Invitrogen), anti-BCL-2 HRP (sc-7382, Santa Cruz), anti-vinculin (V9131, Sigma-Aldrich), anti-P-p70S6K (9234S, Cell Signaling Technology), anti-p70S6K (9202S, Cell Signaling Technology), anti-GFP, anti-PARP (9542S, Cell Signaling Technology), and anti-β actin (A5441, Sigma-Aldrich). The pcDNA3.1 vector bearing the sequence of the nondegradable human gene *MCL1* with mutated ubiquitination sites, referred to as MCL-1^K/R^ was kindly provided by Professor Marc Diederich [22]. Corresponding empty control plasmids were used in parallel. pcDNA3.1-hMCL-1 was a gift from Roger Davis (Addgene plasmid 25375, Cambridge, MA, USA) and is indicated in the text as WT MCL-1. BCL-XL overexpression was achieved using a pcDNA3.1(+) vector encoding human BCL-XL. The MCL-1 5′UTR sequence inserted into the pcDNA3.1 plasmid to generate a GFP reporter construct was GCGGCCGCGCAACCCTCCGGAAGCTGCCGCCCCTTTCCCCTTTTATCGGAATACTTTTTTTAAAAAAAAAGAGTTCGCTGGCGCCACCCCGTAGGACTGGCCGCCCTAAAAGTGATAAAGGAGCTGCTCGCCACTTCTCACTTCCGCTTCCTTCCAGTAAGGAGTCGGGGTCTTCCCCAGTTTTCTCAGCCAGGCGGCGGACTGGCAGAATTC. A scrambled MCL-1 5′UTR sequence served as a negative control: GCGGCCGCAGTTTTTAGTACAGCAGCCCCCCATAACGGCGCCGCCTAGCGCTCAGTAGTCTTTTAGGCGTTGGGAACAGTGCTGCACGATAGGGTCGTCTCCAGCGGGCCATTGTTGCATACACCATACCGCTGGCGTTATCCCTGTGCATCCGGGCTCATCCGCCAACCTGGTACCACTAGCATCTTATCCCAAAGGGCCGACCATTTCCCACGGAATTC.

OCI-LY-1 BAX/BAK knockdown cells were transfected with siBAX and siBAK using the Amaxa® Cell Line Nucleofector® as described above. Briefly, 3 × 10^6^ cells were transfected as indicated with 500 nM siCTRL (ON-TARGET plus, Non-targeting Control pool, from Dharmacon), 500 nM siBak (hs.Ri.BAK 13.1, from IDT), and 500 nM siBax (hs.Ri.BAX 13.2, from IDT).

### Apoptosis assay

Cells (5 × 10^5^ cells/ml) were treated as indicated in the Results, pelleted by centrifugation, and incubated with annexin V-FITC/7-AAD or annexin V-APC in the presence of annexin V binding buffer. Cell suspensions were analyzed with an Attune® Acoustic Focusing Flow Cytometer (Applied Biosystems). Cell death by apoptosis was scored by quantifying the population of annexin V-FITC-positive cells (blue laser; BL-1) or annexin V-APC-positive cells (red laser; RL-1). Flow cytometric data were plotted and analyzed using FlowJo software (version 10).

### Caspase activity assay

Cells (5 × 10^5^ cells/ml) were harvested and washed with pre-warmed PBS. Cells were resuspended in modified Krebs solution and loaded with the NucView 488 dye (final concentration of 5 µM) for 15 min at room temperature, protected from light. The sample was treated as indicated in the Results. Fluorescence was measured in the green detection channel (excitation/emission: 485/515 nm). Flow cytometric data were plotted and analyzed using FlowJo software (version 10).

### RNA extraction and RT-µPCR analysis

Cells were harvested and centrifuged for 5 min at 500 × *g*. RNA was extracted using the HighPure RNA Isolation kit (Roche, Mannheim, Germany; # 11828665001) according to the manufacturer’s protocol. cDNA was prepared using the High-Capacity cDNA Reverse Transcription kit (Applied Biosystems, Brussels, Belgium; # 4368814) according to the manufacturer’s protocol. mRNA was amplified using GoTaq Green master mix (Promega, Leiden, The Netherlands; # M7112) and specific primers for the mRNA of interest. For qPCR, forward and reverse primers for the genes of interest were mixed with FastStart Universal SYBR Green Master (Rox; Roche). For *Mcl-1* (forward 5′-CATTCCTGATGCCACCTTCT-3′, reverse 5′-TCGTAAGGACAAAACGGGAC-3′) and housekeeping mRNA GAPDH (forward 5′-TCAAGAAGGTGGTGAAGCAGG-3′, reverse 5′- ACCAGGAAATGAGCTTGACAAA-3′) (IDT, Leuven, Belgium). β-actin primers were ordered at Thermo Fisher (4333762F). All primers were coupled to a FAM reporter dye. Five µl of this mixture was pipetted in duplicate per condition in a 384-well plate after which 5 µl of a 1:10 dilution of the prepared cDNAs was added. Reactions were performed using a ViiA7 Real-Time PCR System (Thermo Fisher Scientific). For analysis, ΔΔCt values were determined for each condition using GAPDH and β-actin as reference genes before normalizing to the untreated condition.

### Western blot analysis

Cells were washed with phosphate-buffered saline and incubated at 4 °C with lysis buffer (20 mM Tris−HCl (pH 7.5), 150 mM NaCl, 1.5 mM MgCl_2_, 0.5 mM dithiothreitol, 1% Triton X-100, and one tablet of complete EDTA-free protease inhibitor (Thermo Scientific, Brussels, Belgium)) for 30 min. Cell lysates were centrifuged for 5 min at 12,000 × *g* and analyzed by Western blotting as previously described [23]. Western blot quantification was performed using Image Lab 5.2 software.

### Cytosolic Ca^2+^ measurements

OCI-LY-1 cells were seeded in poly-L-lysine-coated 96-well plates (Greiner Bio One, Vilvoorde, Belgium) at a density of 5 × 10^5^ cells/ml. The cells were loaded for 30 min with 1.25 µM Fura-2-AM at 25 °C in modified Krebs solution, followed by a 30-min treatment with the compounds of interest. Fluorescence was monitored on a FlexStation 3 microplate reader (Molecular Devices, Sunnyvale, CA, USA) by alternately exciting the Ca^2+^ indicator at 340 and 380 nm and collecting emitted fluorescence above 510 nm, as described previously [24].

### Live cell imaging

OCI-LY-1 cells (5 × 10^6^) were transfected with a pcDNA3.1 plasmid bearing either the CMV 5′ UTR, a scrambled MCL-1 5′ UTR or the original 5′ UTR. All vectors expressed GFP, and both cell confluence and GFP intensity were measured. Following transfection, the cells were incubated in the IncuCyte® Live Cell Analyzer, and microscopic pictures (Nikon 10x objective) were taken every 2 h. After 4 h, the cells were treated with 10 µM pan-caspase inhibitor ZVAD-OMe-FMK and 30 min later with 10 µM vehicle, TF-BAPTA-AM or BAPTA-AM. The cell plate was subsequently placed in IncuCyte® for another 20 h.

### Metabolic flux analysis

Glycolysis was measured with the Seahorse Glycolysis Stress Test on a Seahorse XFe24 Analyzer (Agilent Technologies, Heverlee, Belgium), which determines the extracellular acidification rate (ECAR) as a measure of glycolytic activity. OCI-LY-1 cells (5 × 10^5^ cells/ml) were pretreated for 1 h in Seahorse Seahorse XF base medium supplemented with glutamine (103334-100, Agilent Technologies, Heverlee, Belgium) in a CO_2_-free incubator. After pretreatment, the ECAR was measured after the subsequent addition of glucose (10 mM final concentration), oligomycin (1 µM final concentration) and 2-deoxyglucose (2-DG, 50 mM final concentration) to assess normal glycolytic activity, maximal glycolytic activity and the nonglycolytic acidification level, respectively. Afterwards, protein concentrations in each well were measured using the BCA assay and used for normalization.

### Extracellular lactate assay

OCI-LY-1 cells (5 × 10^5^ cells/ml) were washed twice in pre-warmed PBS and resuspended in Seahorse XF base medium supplemented with glutamine (103334-100, Agilent Technologies, Heverlee, Belgium). Cells were treated with compounds of interest as indicated, and glucose (10 mM) was added 30 min after treatment. Extracellular lactate was measured according to the manufacturer’s protocol using the Lactate-Glo™ Assay kit (J5021, Promega, Leiden, The Netherlands).

### Tracer metabolomics analysis

OCI-LY-1 cells were seeded at 3 × 10^5^ cells/ml in IMDM without glucose (AL230A, HiMedia, Mumbai, India) and supplemented with 4.5 mg/ml ^13^C or ^12^C glucose, 10% dialyzed FBS (A3382001, Thermo Scientific, Brussels, Belgium) and 2% penicillin/streptomycin (Life Technologies, Brussels, Belgium). After 24 h, the cells were treated with vehicle or the indicated compound. One hour later, the cells were centrifuged at 1500 × *g* for 5 min at 4 °C, washed with 1 ml of ice-cold NaCl (150 mM in H_2_O) and again centrifuged under the same conditions. Subsequently, the washing solution was removed, and 150 µl of extraction buffer (80% MeOH at −80 °C) was added using a precooled pipet tip. Cells were vortexed until the pellet was completely dissolved. The solution was centrifuged at 20,000 × *g* for 15 min at 4 °C, the supernatant was used for further analysis, and the protein pellet was used to measure the protein concentration (BCA assay).

Ten microliters of each supernatant sample were loaded into a Dionex UltiMate 3000 LC System (Thermo Scientific Bremen, Germany) equipped with a C-18 column (Acquity UPLC -HSS T3 1. 8 µm; 2.1 × 150 mm, Waters) coupled to a Q Exactive Orbitrap mass spectrometer (Thermo Scientific) operating in negative ion mode. A step gradient was carried out using solvent A (10 mM TBA and 15 mM acetic acid) and solvent B (100% MeOH). The gradient started with 0% solvent B and 100% solvent A and remained at 0% B until 2 min post injection. A linear gradient to 37% B was carried out until 7 min and increased to 41% until 14 min. Between 14 and 26 min, the gradient increased to 100% B and remained at 100% B for 4 min. At 30 min, the gradient returned to 0% B. The chromatography was stopped at 40 min. The flow was kept constant at 250 µl/min, and the column was placed at 25 °C throughout the analysis. The MS operated in full scan mode (*m*/*z* range: [70–1050]) using a spray voltage of 3.2 kV, capillary temperature of 320 °C, sheath gas at 10.0, and auxiliary gas at 5.0. The AGC target was set at 3e6 using a resolution of 140,000, with a maximum IT fill time of 512 ms. Data collection was performed using Xcalibur software (Thermo Scientific). The data analysis was performed by integrating the peak areas (El-Maven–Polly–Elucidata).

### PFKFB3 kinase activity assay

#### Biochemical assay principle

PFKFB3 enzyme activity was estimated by calculating the amount of ADP generated in a kinase reaction. ADP generation was measured using an ADP Glo kit (Promega, Leiden, The Netherlands) with no deviation from the recommended protocol.

#### Kinase activity assay

PFKFB3 kinase activity was measured according to a published protocol with slight modifications [25]. Briefly, 2X base buffer was prepared as a standard for all kinase reactions. This buffer contained 100 mM HEPES (pH 7.5), 200 mM KCl, 10 mM MgCl_2_, 8 mM dithiothreitol, 0.02% Triton X-100, 0.02% BSA and 4 mM fructose- 6-phosphate. Immediately prior to starting the assay, kinase enzyme was added to the base buffer at a 2X concentration of 40 nM. Each well with PFKFB3 enzyme received 2 µl of the 2X enzyme/base buffer solution. Compounds of interest (1 µl) were added, followed by a 30-min preincubation. Next, 1 µl containing 80 µM ATP was added (giving a final concentration of 20 µM ATP, 2 mM fructose-6-phosphate and 20 mM enzyme) to start the reaction. The kinase reaction was stopped after 2 h by adding 4 µl of ADP-Glo Reagent. One hour later, 8 µl of ADP-Glo Detection Reagent was added. After an additional hour, a PerkinElmer Envision® with an enhanced luminescence module was used to measure the luminescence signal generated in each well. For background subtraction, wells receiving ATP but enzyme-free base buffer and without compound addition were used. To verify the potential effect of compounds on the ADP-Glo kit enzymes (counter assay), wells with compounds received enzyme-free base buffer.

### Molecular docking

The PFKFB3 protein structure in dimeric form was retrieved from the PDB (3qpv) [26] and prepared for docking using the protonate3D functionality implemented in MOE (Molecular Operating Environment (MOE), 2020.09 Chemical Computing Group ULC, 1010 Sherbooke St. West, Suite #910, Montreal, QC, Canada, H3A 2R7, 2022.). The BAPTA (and EGTA) ligand was also modeled in MOE using the mmff94x forcefield as a deprotonated ligand. FTmap was used to identify the potential ligand binding sites both in the monomer and at the dimer interface. Each site was next used for docking of the BAPTA ligand using GOLD with standard parameters [27]. The docking score was calculated using the GOLDfitness score. Induced-fit docking was performed on the top scoring pockets using MOE, and the free energy of binding of the ligand to the receptor was calculated using the GBVI/WSA method (Molecular Operating Environment (MOE), 2020.09 Chemical Computing Group ULC, 1010 Sherbooke St. West, Suite #910, Montreal, QC, Canada, H3A 2R7, 2022).

### Statistical analysis

All statistical tests were performed using Prism 7 (GraphPad, La Jolla, CA, USA). Two-group comparisons were made using Student’s *t* test assuming equal variances. Multiple groups were analyzed by one-way ANOVA with Greenhouse-Geisser corrections and *p* values were included. Unless otherwise indicated, all data are presented as the mean ± S.D. with a significant *p* value (*p < 0.05, **p < 0.01, ***p < 0.001, ****p < 0.0001). Data points were only excluded when they were a significant outlier. Outliers were calculated using the Grubbs’ test (alpha 0.05).

## Results

### BAPTA_i_ induces apoptosis in hematological cell lines

**Fig. 1:**
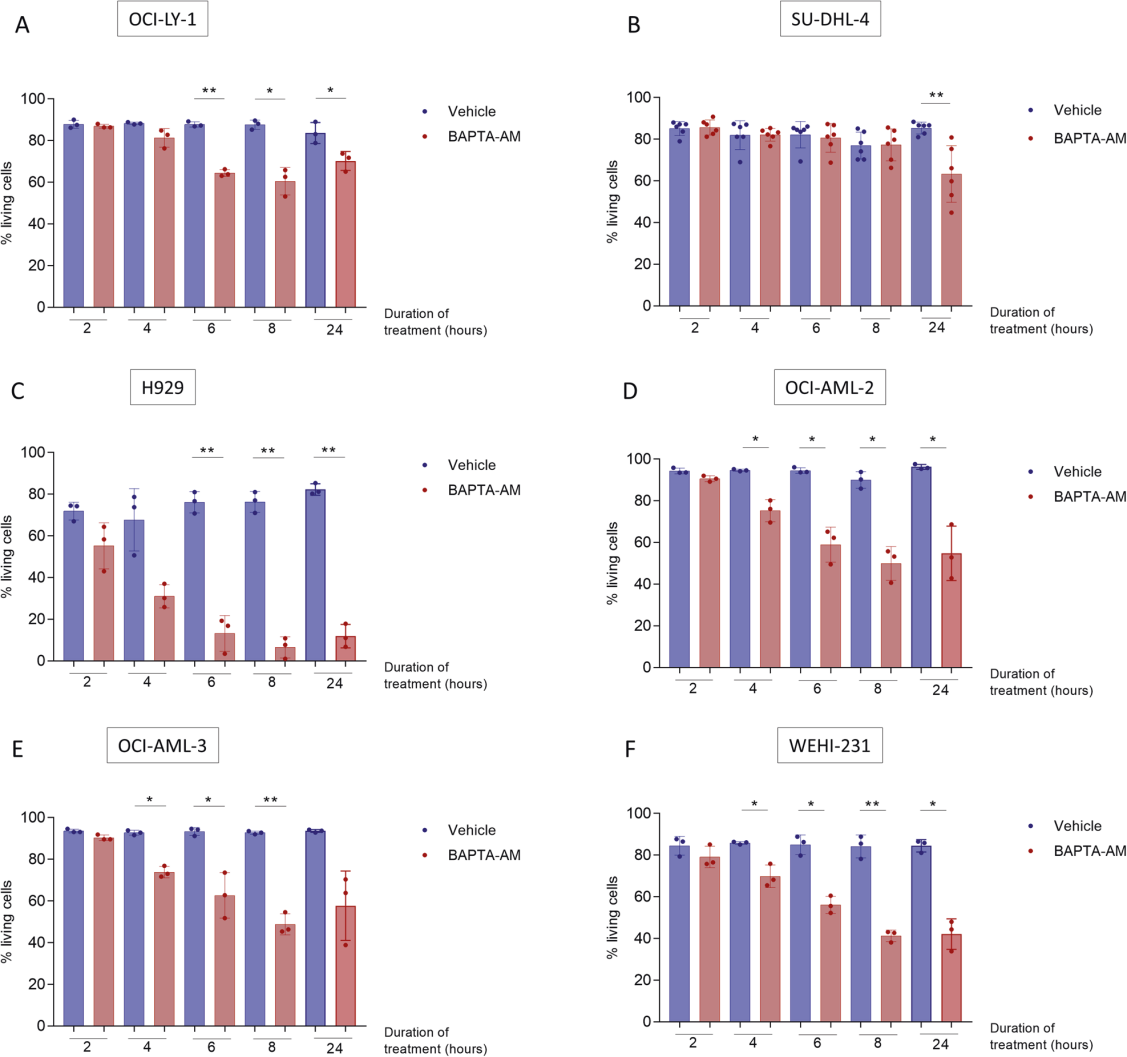
BAPTA_i_ induced apoptosis in multiple hematological lines. Quantitative analysis of apoptosis of OCI-LY-1 (**A**, *N* = 3), SU-DHL-4 (**B**, *N* = 6), H929 (**C**, *N* = 3), OCI-AML-2 (**D**, *N* = 3), OCI-AML-3 (**E**, *N* = 3) and WEHI-231 (**F**, *N* = 3) cells at different time points after the addition of vehicle (DMSO, blue) or 10 µM BAPTA-AM (red). Cells were stained with annexin V-FITC and 7-AAD, and the apoptotic fraction was identified as annexin V-positive cells. Data are represented as the average ± S.D. (*N* = 3). Statistical significance of differences was determined with a paired, two-tailed *t*-test. Differences were considered significant when *p* < 0.05 (**p < 0.01; ***p < 0.001).

We first explored whether hematological cell lines could be addicted to Ca^2+^ signaling for their survival using five different cell lines, i.e., OCI-LY-1 and SU-DHL-4 (DLBCL), H929 (multiple myeloma), OCI-AML-2 and OCI-AML-3 (acute myeloid leukemia). We also included WEHI-231 as a B lymphocyte cell line. We applied 10 μM BAPTA-AM to the extracellular environment to load the cells with BAPTA_i_ and measured cell death using flow cytometry. All cell lines displayed different kinetics and extents of apoptosis in response to BAPTA_i_, with H929 cells being most sensitive and SU-DHL-4 least sensitive (Fig. 1 and Supplementary Fig. 1). To identify the underlying molecular mechanisms, we mainly focused on OCI-LY-1 cells since these cells (1) exhibited a reasonable amount of cell death upon BAPTA_i_ treatment and (2) could be benchmarked against a closely related DLBCL cell line (SU-DHL-4) that is not sensitive to BAPTA_i_.

### BAPTA_i_ elicits decreased abundance of MCL-1 in both SU-DHL-4 and OCI-LY-1 cells

**Fig. 2:**
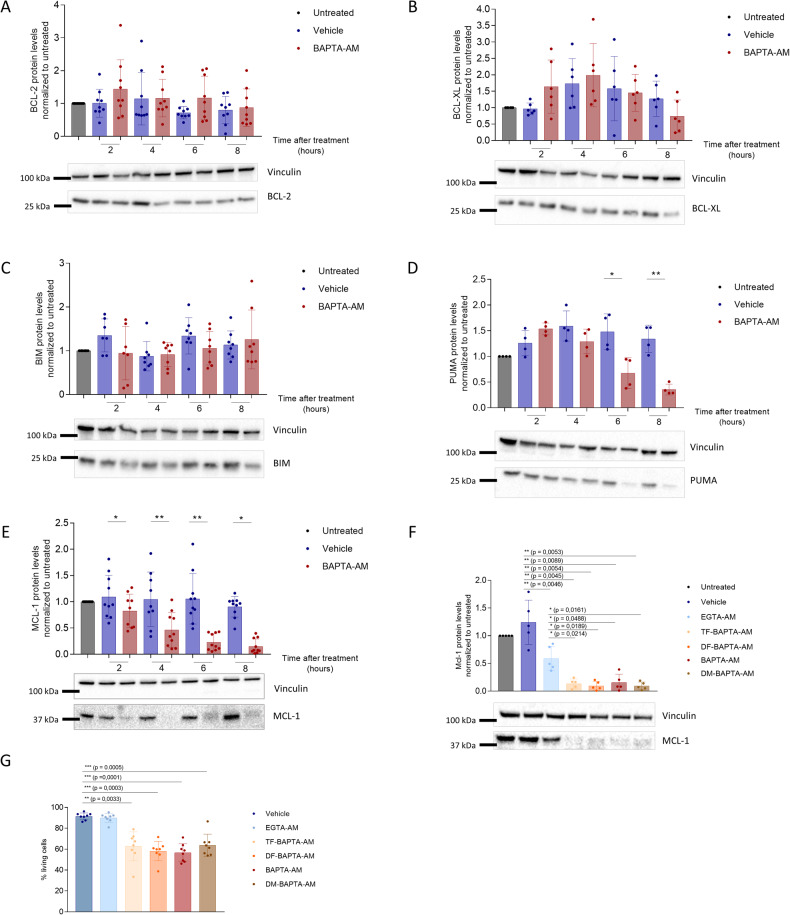
BAPTA_i_ caused MCL-1 downregulation and cell death in a Ca^2+^-independent manner. Representative western blot and statistical analysis of BCL-2 (**A**, *N* = 9), BCL-XL (**B**, *N* = 6), BIM (**C**, *N* = 8), PUMA (**D**, *N* = 4) and MCL-1 (**E**, *N* = 10) levels normalized to untreated in response to vehicle (dark blue) and 10 µM of BAPTA-AM (red) after 2, 4, 6, and 8 h in OCI-LY-1 cells. Vinculin was included as a loading control. Data are represented as the average ± S.D. Statistically significant differences were determined with a paired two-tailed Student’s *t* test. Differences were considered significant when *p* < 0.05 (**p < 0.01; ***p < 0.001). **F** Representative western blot and densitometric analysis of MCL-1 levels in response to a 6 h treatment with of vehicle (dark blue), 10 µM EGTA-AM (light blue), TF-BAPTA-AM (yellow), DF-BAPTA-AM (orange), BAPTA-AM (red) and DM-BAPTA-AM (brown). Vinculin was included as a loading control. Data are represented as the average ± S.D. (*N* = 6). Statistically significant differences were determined with a paired ANOVA test. Differences were considered significant when *p* < 0.05 (**p < 0.01; ***p < 0.001). **G** Quantitative analysis of apoptosis in OCI-LY-1 in response to 6 h treatment with vehicle, 10 µM of EGTA-AM, TF-BAPTA-AM, DF-BAPTA-AM, BAPTA-AM and DM-BAPTA-AM. Cells were stained with annexin V-FITC and 7-AAD and the apoptotic fraction was identified as annexin V-positive cells. Data are represented as the average ± S.D. (*N* = 8). Statistically significant differences were determined with a paired ANOVA test. Differences were considered significant when *p* < 0.05 (**p < 0.01; ***p < 0.001).

Since DLBCL cells are highly dependent on the presence of anti-apoptotic BCL-2-family members to counteract apoptosis, we investigated whether BAPTA_i_ could differentially impact their protein levels in OCI-LY-1 versus SU-DHL-4 cells. Immunoblotting analysis of OCI-LY-1 and SU-DHL-4 cells treated with BAPTA_i_ for different durations revealed no significant differences in BCL-2-, BCL-XL- and BIM-protein levels compared to vehicle-treated cells (Fig. 2A–C for OCI-LY-1; Supplementary Fig. 2A–C for SU-DHL-4). However, MCL-1-protein levels rapidly decreased upon BAPTA_i_ treatment in both OCI-LY-1 and SU-DHL-4 cells, starting from 4 h (Fig. 2E and Supplementary Fig. 2E). Also PUMA-protein levels decreased in both DLBCL cell lines, albeit from 6 h onwards (Fig. 2D and Supplementary Fig. 2D), which aligns with its half-life of about 4 h [28].

To ensure that decreased MCL-1-protein levels were caused by intracellular BAPTA and not extracellular BAPTA, we applied BAPTA as a free acid (abbreviated BAPTA_e_ for extracellular BAPTA), which cannot enter the cell. In contrast to BAPTA_i_, BAPTA_e_ failed to provoke a decrease in MCL-1-protein levels, indicating that the latter effect is indeed caused by intracellularly loaded BAPTA (Supplementary Fig. 3).

### BAPTA_i_ decreases MCL-1-protein levels and provokes cell death in a Ca^2+^-independent manner

The above results imply that Ca^2+^ signals, buffered by BAPTA_i_, are essential to maintain adequate MCL-1-protein levels. We therefore determined whether the ability of different BAPTA analogs to chelate intracellular Ca^2+^ correlated with their efficacy in decreasing MCL-1 levels and limit OCI-LY-1 cell survival. We compared the cell death-inducing properties of BAPTA_i_ in OCI-LY-1 cells with three BAPTA_i_ analogs with varying Ca^2+^-binding affinities (Supplementary Table 1). We included EGTA_i_ (loaded as EGTA-AM) as a control, which has a similar Ca^2+^-binding affinity to BAPTA, but slower binding kinetics and a different molecular structure lacking the two benzene rings. Moreover, EGTAi eliminates effects caused by the AM residue after hydrolysis. First, we validated the distinct Ca^2+^-buffering properties of the different BAPTA_i_ derivatives. We performed cytosolic Ca^2+^ measurements in Fura-2-loaded OCI-LY-1 cells. The different BAPTA analogs dampened anti-IgG/IgM-induced and ionomycin-induced cytosolic [Ca^2+^] rises in line with their respective theoretical *K*_D_ values for Ca^2+^. Among the analogs, DM-BAPTA_i_ displayed the highest potency, while TF-BAPTA_i_ had minimal impact (Supplementary Fig. 4). Therefore, we employed TF-BAPTA_i_ as a control in subsequent experiments to investigate the role of Ca^2+^ buffering in the cellular effects of BAPTA_i_. Next, we analyzed the effects of the different BAPTA_i_ anologs on MCL-1-protein levels and cell death in OCI-LY-1 cells (Fig. 2F, G). Regardless of their different Ca^2+^-buffering capacity, all BAPTA_i_ analogs similarly decreased MCL-1-protein levels and evoked cell death in OCI-LY-1 cells. Compared to BAPTA_i_, EGTA_i_ appeared less effective in reducing MCL-1 abundance and failed to provoke cell death, although EGTA_i_ buffered the Ca^2+^ responses more avidly than TF-BAPTA_i_ and DF-BAPTA_i_. This observation also excludes that the effects of BAPTA_i_ on MCL-1 and cell death were due to the released AM moiety. Finally, we validated that BAPTA_i_ and its derivatives provoked cell death through apoptosis: (1) Cell death induced by BAPTA_i_ and its derivatives could be rescued by ZVAD-OMe-FMK, a pan-caspase inhibitor (Supplementary Fig. 5A), and (2) BAPTA_i_ and its derivatives provoked the cleavage of PARP, a downstream target of caspase 3. Additionally, in these assays, EGTA_i_ did not have major effects (Supplementary Fig. 5B).

### BAPTA_i_-induced cell death is dependent on decreased abundance of MCL-1

**Fig. 3:**
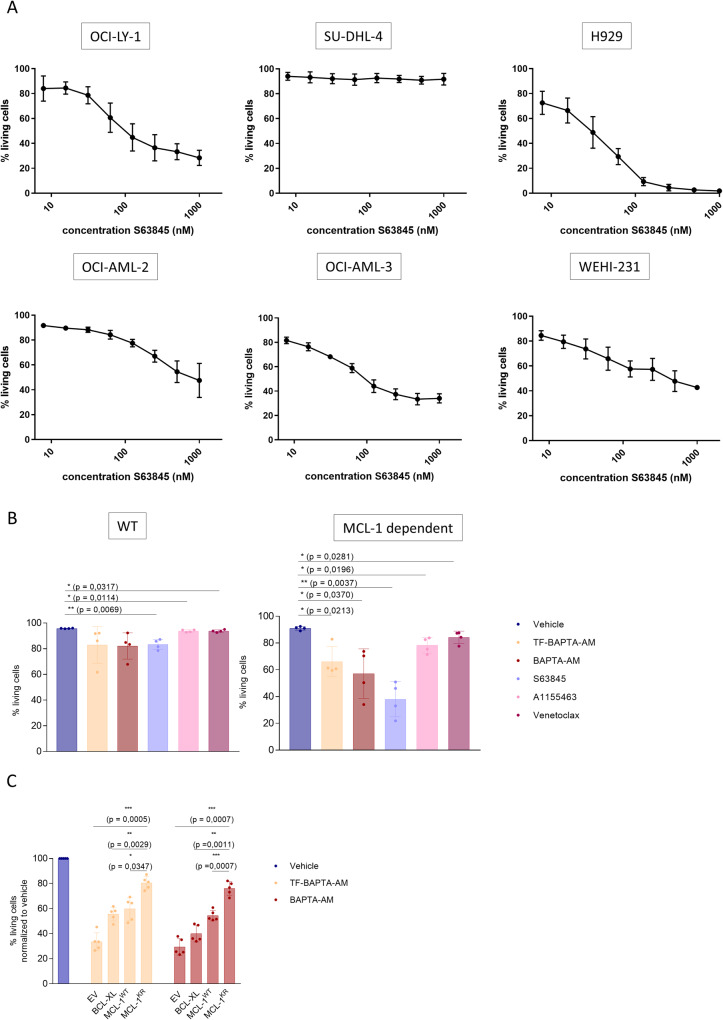
BAPTA_i_-induced cell death is dependent on decreased abundance of MCL-1 proteins and is prevented by reinforcing MCL-1-protein levels. **A** Concentration-response curve of the MCL-1 inhibitor S63845 on cell survival in OCI-LY-1, SU-DHL-4, H929, OCI-AML-2, OCI-AML-3 and WEHI-231 cells (left to right) after 6 h of treatment. Cell death was measured using annexin V-FITC and 7-AAD staining, and the apoptotic fraction was identified as annexin V-positive cells. (*N* = 3). **B** WT (parental control) and MCL-1-dependent SVEC cell lines were treated for 6 h with vehicle (dark blue), 10 µM TF-BAPTA-AM (yellow), BAPTA-AM (red), MCL-1 inhibitor S63845 (purple), 1 µM BCL-XL inhibitor A1155483 (pink) or 10 µM BCL-2 inhibitor venetoclax (burgundy). Cells were stained with annexin V-APC and 7-AAD, and cell death was measured via flow cytometry. Data are represented as the average ± S.D. (*N* = 4). Statistically significant differences were determined with a paired ANOVA test. Differences were considered statistically significant when *p* < 0.05 (**p < 0.01; ***p < 0.001, ****p < 0.0001). **C** OCI-LY-1 cells were transfected with an empty vector (EV), a BCL-XL-overexpressing plasmid, a wild-type (WT) MCL-1-overexpressing plasmid or a plasmid overexpressing a nondegradable KR mutant of MCL-1. Statistical analysis of cell death in transfected OCI-LY-1 cells after 6 h of 10 µM TF-BAPTA-AM or BAPTA-AM treatment normalized to vehicle. Cells were stained with annexin V-APC and 7-AAD, and apoptosis was measured via flow cytometry. Data are represented as the average ± S.D. (*N* = 5). Statistically significant differences were determined with a paired ANOVA test. Differences were considered significant when *p* < 0.05 (**p < 0.01; ***p < 0.001, ****p < 0.0001).

While BAPTA_i_ decreased MCL-1-protein levels in OCI-LY-1 and SU-DHL-4 cells, it primarily induced cell death in OCI-LY-1 cells. We thus asked whether the difference in BAPTA_i_ sensitivity could be due to differences in MCL-1 dependence. We exposed all six cell lines to varying concentrations of S63845, a validated, high-affinity, BH3-mimetic MCL-1 antagonist [29]. As shown in Fig. 3A, S63845 caused a concentration-dependent death of OCI-LY-1, H929, OCI-AML-2, OCI-AML-3 and WEHI-231 cells but had no effect in SU-DHL-4 cells. These results indicate that sensitivity to BAPTA_i_ correlates with sensitivity to MCL-1 inhibition. Furthermore, we evaluated the protein levels of different BCL-2-family members in all six cell lines (Supplementary Fig. 6). H929 cells displayed the highest MCL-1-protein levels correlating with its high sensitivity toward S63845 and BAPTA_i_.

To underpin the involvement of MCL-1 in cell death caused by BAPTA_i_ and BAPTA_i_ analogs, we engaged two independent approaches. To ensure that our findings were not due to heterogeneous cellular genetic backgrounds among the different cell lines, we implemented an isogenic cell system using endothelial cells (SVEC) with genetically engineered dependence toward MCL-1 [20]. “Mito-priming” is based on the equimolar co-expression of pro- and anti-apoptotic BCL-2 proteins to artificially generate an addiction to a specific anti-apoptotic BCL-2 protein. Wild-type (WT, parental control) and MCL-1-dependent SVECs were treated with TF-BAPTA-AM and BAPTA-AM (Fig. 3B). MCL-1 dependence was benchmarked using specific antagonists for MCL-1 (S63845), BCL-2 (venetoclax) and BCL-XL (A1155483). MCL-1-addicted SVECs only died in response to S63845, while the WT parental SVEC control cells were much less sensitive. Both BAPTA_i_ and TF-BAPTA_i_ induced significant cell death in MCL-1-dependent SVEC cells but were only marginally effective in the WT parental SVEC control cells. These results indicate that BAPTA_i_-induced cell death depends on cell addiction to MCL-1 for survival and is not a cell type- or a hematological cancer-related phenomenon.

Since MCL-1 is a short-lived protein, we examined whether enhancing MCL-1-protein levels could alleviate sensitivity to BAPTA_i_. We transfected OCI-LY-1 cells with a ubiquitination-deficient MCL-1 mutant in which the established ubiquitination sites (amino acids 5, 40, 136, 194 and 197) were mutated from lysines into arginines (MCL-1^K/R^) [30]. To evaluate the effectiveness of the nondegradable MCL-1^K/R^ mutant, parallel cultures were transfected with either an empty vector (EV) or a vector overexpressing wild-type MCL-1 (MCL-1^WT^) (Supplementary Fig. 7A). The half-life of MCL-1^K/R^ was substantially delayed compared to endogenous MCL-1 and overexpressed MCL-1^WT^ (Supplementary Fig. 7B). We also overexpressed BCL-XL to confirm the specific mediation of the effects by MCL-1 rather than just any BCL-2-family member. Immunoblot analysis showed that BAPTA_i_ strongly reduced endogenous (EV) and MCL-1^WT^. In contrast, BAPTA_i_ and TF-BAPTA_i_ did not affect BCL-XL- and the nondegradable MCL-1^K/R^-protein levels (Supplementary Fig. 7A). However, only MCL-1^K/R^ mutant, not MCL-1^WT^ or BCL-XL, protected against BAPTA_i_ and TF-BAPTA_i_-induced apoptosis (Fig. 3C). Thus, the decrease in MCL-1-protein levels appears to be the cause for BAPTA_i_-induced cell death in OCI-LY-1 cells.

### BAPTA_i_ reduces MCL-1-protein levels by suppressing mTORC1, an important driver of *Mcl-1* translation

**Fig. 4:**
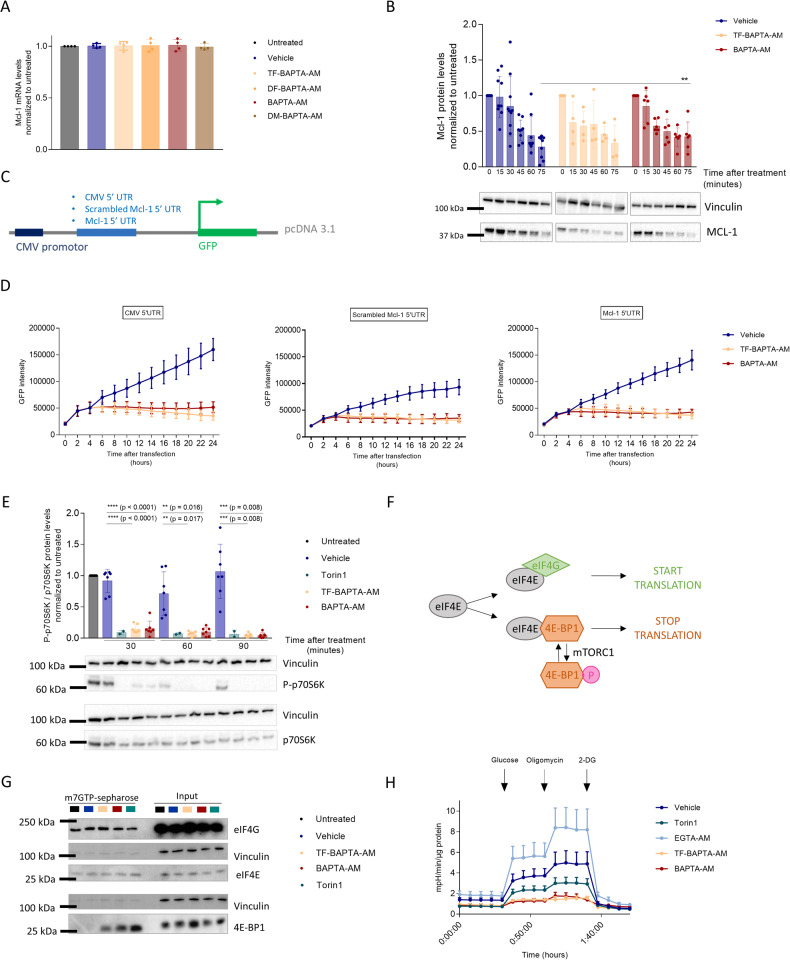
BAPTA_i_ inhibits translational activity by hampering glycolysis. **A** Statistical analysis of *Mcl-1* mRNA levels after 6 h treatment with vehicle (dark blue), 10 µM TF-BAPTA-AM (yellow), DF-BAPTA-AM (orange), BAPTA-AM (red) and DM-BAPTA-AM (brown) normalized to untreated (black) Data are represented as the average ± S.D. (*N* = 4). Statistically significant differences were determined with a paired ANOVA test. Differences were considered significant when *p* < 0.05 (**p < 0.01; ***p < 0.001). **B** Representative western blot and densitometric analysis of MCL-1-protein levels after 0, 15, 30, 45, 60 and 75 min of vehicle (dark blue, *N* = 10), 10 µM TF-BAPTA-AM (yellow, *N* = 4) and BAPTA-AM (red, *N* = 6) treatment. Prior to this, the cells were pretreated with 20 µg/ml cycloheximide. Quantified MCL-1 levels were normalized to the loading control (vinculin) and calculated relative to the untreated condition. **C** Schematic representation of the constructs used in (**D**). A pcDNA3.1 reporter vector was inserted with the original (Mcl-1) or a scrambled (control, CTRL) 5’UTR region of *Mcl-1*. A construct with the original CMV 5’ UTR was included as an additional control. OCI-LY-1 cells were subsequently electroporated with one of the constructs, and GFP intensity was measured. **D** Graphical representation of GFP intensity after transfection with one of the aforementioned constructs. After 4 h, transfected cells were treated with 10 µM of the caspase inhibitor ZVAD-OMe-FMK and 30 min later with vehicle (dark blue), 10 µM TF-BAPTA-AM (yellow) or BAPTA-AM (red). Measurement of GFP intensity was continued for 24 h (*N* = 4). **E** Representative western blot and densitometric analysis of P-p706K/total p70S6K normalized to untreated cells after 30, 60 and 90 min of treatment with vehicle (dark blue, *N* = 7), 2 µM Torin1 (green, *N* = 2), 10 µM TF-BAPTA-AM (yellow, *N* = 7) or BAPTA-AM (red, *N* = 7). Quantified (P-)p70S6K levels were normalized to the loading control (vinculin) and calculated relative to the untreated condition. Data are represented as the average ± S.D. Statistically significant differences were determined with a paired ANOVA test. Differences were considered significant when *p* < 0.05 (**p < 0.01; ***p < 0.001). **F** Translation initiation factor eIF4E forms active complexes with eIF4G (green), leading to mRNA recruitment. eIF4G competes with 4E-BP1 (orange), which forms inactive complexes with eIF4E, leading to inhibition of translation. Phosphorylation of 4E-BP1 by mTORC1 causes its release from eIF4E. **G** Western blot of protein extracts from OCI-LY-1 cells treated for 90 min with vehicle (dark blue), 10 µM TF-BAPTA-AM (yellow), BAPTA-AM (red) and 2 µM Torin1 (green). Lysates were incubated with m7GTP-sepharose beads, and bound proteins were denatured and subjected to western blotting to reveal eIF4E bound to eIF4G (active complexes) or to 4E-BP1 (inactive complexes). Vinculin was included as a loading control (*N* = 3). **H** OCI-LY-1 cells were pretreated for 1 h with vehicle (dark blue, *N* = 8), 1 µM Torin1 (green, *N* = 5), 10 µM EGTA-AM (light blue, *N* = 3), TF-BAPTA-AM (yellow, *N* = 8) or BAPTA-AM (red, *N* = 8) in glucose-free medium. All experimental extracellular pH values were measured after the addition of glucose (normal glycolytic activity), oligomycin (maximal glycolytic activity) and 2-DG (nonglycolytic acidification). In each experiment, data were obtained in three-fold and normalized to the amount of protein. Data are represented as the average ± S.E.M.

Next, we examined the mechanism by which BAPTA_i_ evoked a decline in MCL-1-protein levels. Firstly, BAPTA_i_ and its analogs did not affect *Mcl-1*-mRNA levels, indicating the absence of effects on *MCL-1* transcription (Fig. 4A). To determine whether BAPTA_i_ enhanced MCL-1-protein degradation, we pretreated OCI-LY-1 cells with cycloheximide to inhibit the de novo translation of *Mcl-1* (Fig. 4B). Cycloheximide results in a rapid decline in MCL-1-protein levels owing to its rapid turn-over (Supplementary Fig. 8A, B, blue bars). This decline is prevented by bortezomib (a proteasomal inhibitor) but not by bafilomycin A (a lysosomal inhibitor), indicating that MCL-1 is degraded via the proteasome (Supplementary Fig. 8A pink bars and 8B purple bars). Compared to the vehicle control, neither BAPTA_i_ nor TF-BAPTA_i_ accelerated the cycloheximide-induced decline in MCL-1-protein levels (Fig. 4B, yellow and red bars). Thus, BAPTA_i_ and TF-BAPTA_i_ did not enhance MCL-1 degradation. Moreover, the rapid decline in MCL-1 by BAPTA_i_ is not a consequence of caspase activity since caspase are not activated during the first 75 min (Supplementary Fig. 9).

Since BAPTA_i_ neither inhibited *MCL1* transcription nor accelerated MCL-1 degradation, we assessed whether BAPTA_i_ could suppress *Mcl-1* translation. We monitored translation by cloning the 5′ untranslated region (UTR) of *MCL-1* in front of an open reading frame encoding GFP (Fig. 4C). We also cloned the 5′ UTR of CMV to monitor general translation. These constructs were benchmarked against a control that contained a scrambled version of the 5′ UTR of *MCL1*. The latter provides the background translation potentially due to leaky translational events. First, we validated that the 5′ UTR of *MCL-1* and CMV significantly augmented GFP levels compared to the scrambled control (Supplementary Fig. 10). Then, we assessed the impact of BAPTA_i_ and TF-BAPTA_i_ on both *MCL-1* and CMV 5′ UTRs to determine their effect on *Mcl-1* translation and general translation events. Second, BAPTA_i_ and TF-BAPTA_i_ potently decreased *Mcl-1* 5′ UTR- and CMV 5′ UTR-driven GFP levels, indicating that BAPTA_i_ inhibits *Mcl-1* translation and general translation in a Ca^2+^-independent manner (Fig. 4D). In addition to this, we pretreated OCI-LY-1 cells with bortezomib to inhibit proteasomal degradation and subsequently exposed the cells for 3 h to cycloheximide, TF-BAPTA_i_ or BAPTA_i_ (Supplementary Fig. 8C). TF-BAPTA_i_ and BAPTA_i_ had no additional effects on MCL-1-protein levels compared to cycloheximide, providing evidence for that they act by inhibiting protein translation.

Since *Mcl-1* translation is driven by mammalian target of rapamycin complex 1 (mTORC1), we assessed the effect of BAPTA_i_ on the phosphorylation of p70S6K, a downstream target of mTORC1 signaling (Fig. 4E) [31]. OCI-LY-1 cells were treated with vehicle, TF-BAPTA-AM, BAPTA-AM or Torin1, a selective and high-affinity mTORC1 inhibitor. Samples were taken after 30, 60 and 90 min, and the ratio of P-p70S6K/total p70S6K was analyzed via western blot. All treatments similarly and significantly lowered the phosphorylation of p70S6K, indicating that BAPTA_i_ suppresses mTORC1 activity in a Ca^2+^-independent manner. To demonstrate that mTORC1 inactivation preceded MCL-1 downregulation, we treated OCI-LY-1 cells for 1, 2 and 3 h with BAPTA-AM and TF-BAPTA-AM and analyzed the ratio of P-p70S6K/total p70S6K and MCL-1 via western blot. (Supplementary Fig. 11). We observed that p70S6K dephosphorylation precedes the decline in MCL-1-protein levels. These results indicate that BAPTA_i_ may diminish general protein translation by inhibiting mTORC1 activity.

To strengthen the conclusion that BAPTA_i_ inhibits mTORC1-driven protein translation, we examined eIF4E, the rate-limiting regulator of cap-dependent mRNA translation. eIF4E either forms a complex with eIF4G, thereby recruiting mRNA and driving translation, or forms an inactive complex with 4E-BP1, thereby competing with eIF4G for binding eIF4E and limiting translation. Phosphorylation of 4E-BP1 by mTORC1 causes its release from eIF4E to allow cap-dependent translation to proceed (Fig. 4F). Thus, the eIF4E-binding ratio of eIF4G/4E-BP1 provides a molecular readout for mTORC1-driven mRNA translation activity. Therefore, we pulled-down eIF4E using m7GTP-sepharose beads and checked the binding of eIF4G and 4E-BP1 in lysates of OCI-LY-1 cells treated for 90 min with TF-BAPTA-AM, BAPTA-AM or Torin1 as a control (Fig. 4G). All treatments elicited a prominent increase in 4E-BP1 binding to eIF4E compared to the vehicle control, indicating that BAPTA_i_ indeed inhibits cap-dependent mRNA translation and that this occurs independently of its Ca^2+^-chelating properties.

### BAPTA_i_ rapidly suppresses glycolysis in cells preceding cell death

Since mTORC1 activity is tightly controlled by the metabolic state of the cell, we monitored glycolytic activity in OCI-LY-1 and SU-DHL-4 cells exposed to TF-BAPTA_i_ and BAPTA_i_. To eliminate any effect caused by the AM residue or by mTORC1 inhibition itself, EGTA_i_ and Torin1 were included as controls. Using a Seahorse extracellular flux analyzer, we analyzed the extracellular acidification rate (ECAR), which is a measure of glycolytic activity. Although BAPTA_i_-induced cell death was only observed after 4 h with OCI-LY-1 cells (Fig. 1A), pretreatment with the pan-caspase inhibitor ZVAD-OMe-FMK was performed to avoid any potential adverse effects of cell death initiation. Within 1 h, BAPTA_i_ and TF-BAPTA_i_ strongly lowered ECAR, while Torin1, EGTA_i_ or vehicle did not exert such an effect (Fig. 4H). In addition, we performed extracellular lactate measurements to validate that both BAPTA_i_ and TF-BAPTA_i_ lowered extracellular lactate levels (Supplementary Fig. 12).

**Fig. 5:**
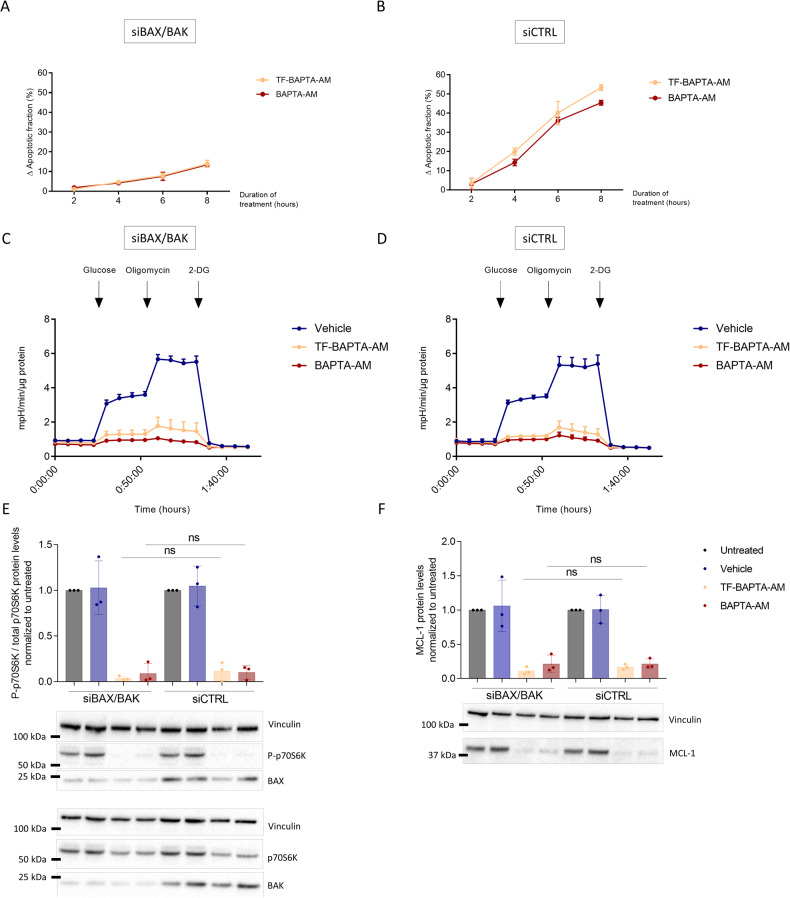
BAPTA_i_-induced inhibition of glycolysis, mTORC1 inactivation and MCL-1 downregulation are the cause and not the consequence of cell death. We generated BAX/BAK-knockdown OCI-LY-1 cells using siBAX and siBAK using siCTRL as a control. BAX/BAK-knockdown (**A**) and control (**B**) OCI-LY-1 cells were treated with vehicle, 10 µM TF-BAPTA-AM or BAPTA-AM for different time periods. Cell death was measured using annexin V-FITC and 7-AAD staining. The apoptotic fraction was identified as annexin V-positive cells normalized to vehicle conditions. Data are represented as the average ± S.D. (*N* = 3). BAX/BAK-knockdown (**C**) and control (**D**) OCI-LY-1 cells were pretreated for 1 h with vehicle (dark blue), 10 µM TF-BAPTA-AM (yellow) or BAPTA-AM (red) in glucose-free medium. All experimental extracellular pH values were measured after the addition of glucose (normal glycolytic activity), oligomycin (maximal glycolytic activity) and 2-DG (nonglycolytic acidification). In each experiment, data were obtained in three-fold and normalized to the amount of protein. Data are represented as the average ± S.E.M (*N* = 3). **E** Representative western blot and densitometric analysis of P-p706K/total p70S6K normalized to untreated cells after 30, 60 and 90 min of treatment with vehicle (dark blue), 10 µM TF-BAPTA-AM (yellow) or BAPTA-AM (red) in BAX/BAK knockdown and control OCI-LY-1 cells. Quantified (P-)p70S6K levels were normalized to the loading control (vinculin) and calculated relative to the untreated condition. Data are represented as the average ± S.D. (*N* = 3). Statistically significant differences were determined with a paired ANOVA test. Differences were considered significant when *p* < 0.05. (**p < 0.01; ***p < 0.001). **F** Representative western blot and densitometric analysis of MCL-1 levels in response to a 6 h treatment with of vehicle (dark blue), 10 µM TF-BAPTA-AM (yellow), or BAPTA-AM (red) in BAX/BAK knockdown and control OCI-LY-1 cells. Vinculin was included as a loading control. Data are represented as the average ± S.D. (*N* = 3). Statistically significant differences were determined with a paired ANOVA test. Differences were considered significant when *p* < 0.05 (**p < 0.01; ***p < 0.001).

To validate that the effects evoked by BAPTA_i_ are the cause and not the consequence of cell death, we silenced BAX/BAK using siRNA in the BAPTA_i_-sensitive OCI-LY-1 cells. BAX/BAK-protein levels were reduced by 60–70% (Fig. 5E, F for blot and Supplementary Fig. 13 for quantification). Next, we validated that BAPTA_i_/TF-BAPTA_i_-induced apoptosis was strongly reduced in siBAX/BAK-treated cells compared to siCTRL-treated cells (Fig. 5A, B). Importantly, the BAPTA_i_/TF-BAPTA_i_-induced decline in glycolytic activity (Fig. 5C, D), mTORC1 activity (Fig. 5E) and MCL-1-protein levels (Fig. 5F) was equally effective in siBAX/BAK- and siCTRL cells, ruling out that these effects were a consequence of apoptotic cell death.

### BAPTA_i_ impairs glycolysis by abrogating the conversion of fructose-6-phosphate into fructose-1,6-bisphosphate

**Fig. 6:**
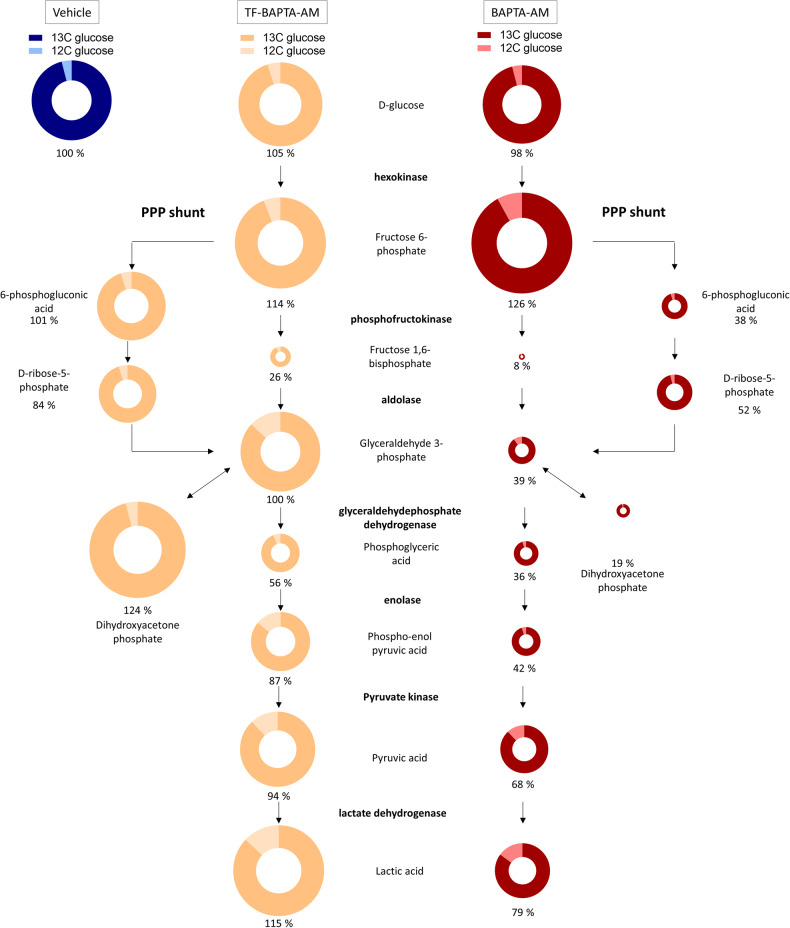
BAPTA_i_ and TF-BAPTA_i_ potently inhibit the conversion of fructose-1-phosphate into fructose-1,6-bisphosphate. Twenty-four hours prior to treatment, OCI-LY-1 cells were cultured in glucose-free IMDM supplemented with ^13^C glucose. Afterwards, the cells were subsequently treated for 1 h with vehicle (dark blue), 10 µM TF-BAPTA-AM (yellow) or BAPTA-AM (red). Abundances and fractional contributions of ^12^C vs. ^13^C for each glycolytic metabolite were measured using mass spectrometry. The size of the doughnut represents the abundance of each metabolite normalized to the vehicle condition; % changes in abundance are indicated beneath each doughnut. The color of the doughnut represents the relative contributions of ^13^C (dark) and ^12^C (light) (*N* = 3).

To unravel the mechanism by which BAPTA_i_ impacts glycolysis, we set up a metabolomics approach using nonradioactive ^13^C glucose (Fig. 6). We cultured OCI-LY-1 cells in the presence of ^13^C-labeled glucose to incorporate the stable ^13^C isotopes into the cells’ metabolic pathways. With mass spectrometry, the abundance and labeling patterns (isotopologs and fractional contribution) of metabolites were detected, thereby assessing the activity and connectivity of metabolic pathways. The cells were treated for 1 h with vehicle, BAPTA-AM or TF-BAPTA-AM. BAPTA_i_ caused a significant decrease in fructose-1,6-bisphosphate levels, which also occurred in response to TF-BAPTA_i_. Downstream metabolites, but not upstream metabolites, were also reduced. This suggests that BAPTA_i_ induces Ca^2+^-independent inhibition of glycolysis at the level of fructose-6-phosphate conversion into fructose-1,6-bisphosphate. Glycolytic metabolites downstream of fructose-1,6-bisphosphate were less reduced by TF-BAPTA_i_ than BAPTA_i_. This could be caused by better maintained activity of the pentose phosphate pathway (PPP), which converts fructose-6-phosphate into glyceraldehyde 3-phosphate that can re-enter the glycolytic pathway, by-passing the block.

### BAPTA directly inhibits the activity of purified, recombinantly expressed PFKFB3

**Fig. 7:**
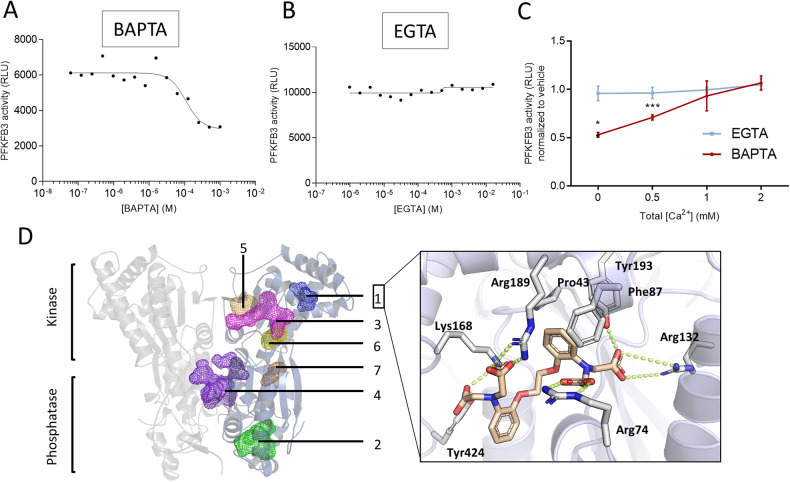
BAPTA directly interacts with PFKFB3, leading to inhibition of its kinase activity. In vitro measurement of PFKFB3 activity in the presence of increasing concentrations of EGTA (**A**) and BAPTA (**B**). **C** In vitro measurement of PFKFB3 activity in the presence of 1 mM BAPTA (red) or 1 mM EGTA (blue) normalized to vehicle (H_2_O) conditions, measured with increasing Ca^2+^ concentrations (0 to 2 mM CaCl_2_) in the assay buffer. **D** The PFKFB3 dimer is represented in two different shades of cartoon. FTmap was used to identify seven putative pockets, which have been mapped on one monomer, including the fructose-6-phosphate site (1), the fructose-2,6-bisphosphate site (2), the ATP-binding site (3), a dimerization site (4) and other minor cavities (5–7). BAPTA was docked to each of the binding pockets leading to seven Goldscores: 83.9, 94.8, 67.3, 74.1, 64.8, 58.4, and 63.7 from pockets 1 to 7, respectively. The highest scoring pockets were further investigated using induced-fit docking yielding a putative BAPTA binding mode to pocket 1 of the X site. BAPTA makes four salt bridges between its four carboxylate functional groups, and Arg 74, Arg 132, Lys 168, and Arg189 are further stabilized by hydrogen bonds with Tyr193 and Tyr424. The aromatic ring is buried between the hydrophobic side chains of Pro43, Phe87 and Tyr193.

The production of fructose-1,6-bisphosphate is catalyzed by the phosphofructokinase 1 (PFK1) enzyme, whose activity depends on fructose-2,6-bisphosphate. The levels of this metabolite are regulated by the activity of the bi-functional 6-phosphofructo-2-kinase/fructose-2,6-bisphosphatase (PFKFB) enzymes. Four different isoforms of PFKFB exist, which display different kinase/phosphatase activity ratios [32]. PFKFB3 has the highest kinase/phosphatase activity ratio, which sustains high glycolytic rates. We therefore asked whether BAPTA could directly impact the master regulator PFKFB3. We measured the kinase activity of the recombinant, purified human PFKFB3 enzyme using a cell-free biochemical assay based on measuring the production of ADP in the presence of different concentrations of BAPTA (ranging from 1 µM to 1 mM). This concentration range was chosen since previous studies indicated that extracellular application of10 μM BAPTA-AM accumulates to cytosolic concentrations of 1–2 mM [18]. In this in vitro kinase assay, BAPTA directly inhibited PFKFB3 activity (Fig. 7A). Of note, PFKFB3 activity was only partially inhibited by BAPTA with a relative IC_50_ of 110 µM (determined by the point halfway between the top and bottom plateaus of the curve). The absolute IC_50_ would be in the mM range. Appropriate controls (counter assay, see “Material and methods”) confirmed that BAPTA did not affect the ATP-depleting and luciferase detection enzymes included in the ADP-Glo system (not shown). We also validated that PFKFB3 activity was inhibited by AZ PFKFB3 67, showing that the assay reports bona fide PFKFB3 activity. (Supplementary Fig. 14). EGTA did not affect PFKFB3 kinase activity (Fig. 7B). Next, we asked whether the inhibition of PFKFB3 by BAPTA was influenced by Ca^2+^. We found that increasing the Ca^2+^ concentrations in the assay buffer alleviated the inhibitory effect of BAPTA (applied at 1 mM) on PFKFB3. Increasing the Ca^2+^ concentration had no impact on PFKFB3 activity in the presence of EGTA (Fig. 7C). It is interesting to note that adding 0.5 mM Ca^2+^ to 1 mM BAPTA thus anticipating ~50% Ca^2+^-free BAPTA and ~50% Ca^2+^-bound BAPTA reduced BAPTA’s inhibitory effect to about 50%. Saturating BAPTA with Ca^2+^ by adding 1 or 2 mM Ca^2+^ to 1 mM BAPTA completely alleviated the inhibitory effect of BAPTA on PFKFB3. This shows that BAPTA inhibits PFKFB3 activity in its Ca^2+^-free form, but not in its Ca^2+^-complexed form.

To further substantiate that BAPTA directly inhibits PFKFB3, we performed a molecular modeling approach by docking BAPTA into the different putative binding sites (Fig. 7D). We identified seven different sites, including the ATP-binding site and the fructose-6-phosphate sites in the kinase domain and the fructose- 2,6-bisphosphate site in the hydrolase domain and a site at the dimerization interface. The docking simulations preferred a binding mode to both fructose-6-phosphate binding sites bound in the kinase and hydrolase domains (with scores of 94.8 and 83.9 over scoring 74.1 to 58.4 in the other domains). This is not surprising, as both domains are hallmarked by a series of positively charged residues to coordinate multiple phosphates. To obtain a more detailed view, induced-fit docking was performed at both sites, hypothesizing that the acid functionalities of BAPTA mimic the fructose-phosphate groups. This resulted in a preferred binding mode of BAPTA to the fructose- 6-phosphate sites within the kinase domain (−10.8 kcal/mol) over the hydrolase site (−9.3 kcal/mol). With EGTA, this dropped to −9.0 kcal/mol. The docking simulations suggest specific binding of BAPTA to the fructose-6-phosphate substrate-binding site in the kinase domain competing with the substrate and ATP-binding site via phosphate-mimicking interactions.

### PFKFB3 inhibition suppresses mTORC1 activity, thereby evoking a decline in MCL-1-protein levels

**Fig. 8:**
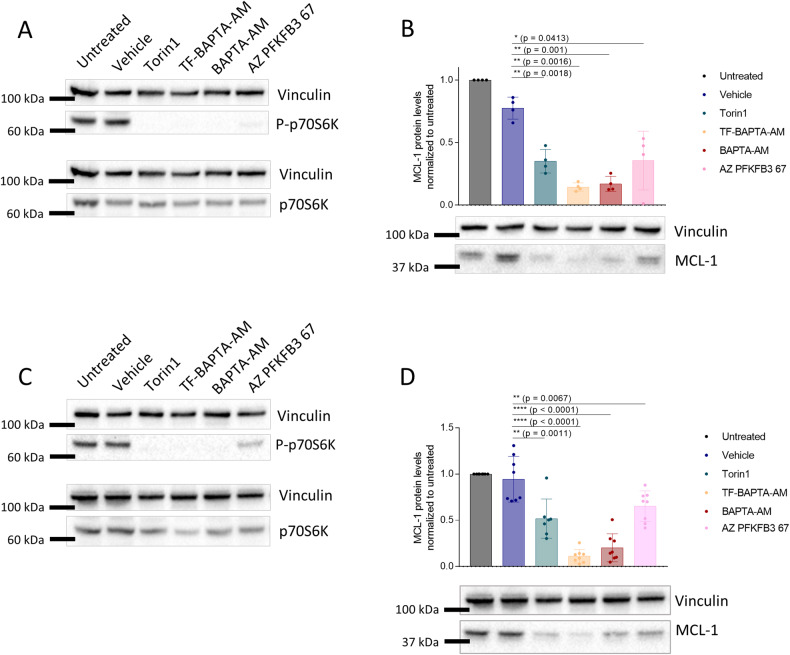
PFKFB3 inhibition evokes mTORC1 inhibition and MCL-1 downregulation in OCI-LY-1 and SU-DHL-4 cells. SU-DHL-4 (**A**, **B**) and OCI-LY-1 (**C**, **D**) cells were treated with vehicle, 2 µM Torin1, 10 µM TF-BAPTA-AM, 10 µM BAPTA-AM or 25 µM PFKFB3 inhibitor (AZ PFKFB3 67). **A**, **C** Representative western blots of P-p70s6K and p70S6K. (*N* = 3). **B**, **D** Representative western blot and densitometric analysis of MCL-1 levels in response to 6 h of treatment with vehicle (dark blue), 2 µM Torin1 (dark green), 10 µM TF-BAPTA-AM (yellow), 10 µM BAPTA-AM (red) or 25 µM PFKFB3 inhibitor (pink). Quantified MCL-1 levels were normalized to the loading control (vinculin) and calculated relative to the untreated condition. Data are represented as the average ± S.D. (*N* = 4 (**B**), *N* = 8 (**D**)). Statistically significant differences were determined with a paired ANOVA test. Differences were considered significant when *p* < 0.05 (**p < 0.01; ***p < 0.001).

Finally, we sought to establish a causal link between the inhibition of PFKFB3 activity, inhibition of mTORC1 activity and a subsequent decrease of MCL-1-protein levels in SU-DHL-4 and OCI-LY-1 cells. We therefore used AZ PFKFB3 67, a PFKFB3 inhibitor [25]. We exposed the cells to 25 µM AZ PFKFB3 67, a concentration limiting PFKFB3 activity in cells [33]. Similarly to Torin1 and BAPTA_i_, AZ PFKFB3 67 decreased the phospho-p70S6K/total p70S6K ratio and reduced MCL-1-protein levels in both SU-DHL-4 (Fig. 8A, B) and OCI-LY-1 cells (Fig. 8C, D); though AZ PFKFB3 67 appeared less potent than BAPTA_i_ and TF-BAPTA_i_. These results indicate that pharmacological inhibition of PFKFB3 is sufficient to suppress mTORC1 activity and downstream signaling events, thereby resulting in a decline in MCL-1-protein levels. Yet, AZ PFKFB3 67 by itself did not induce cell death in any of the MCL-1-dependent hematological cell lines (Supplementary Fig. 15).

## Discussion

Our results reveal an unprecedented and Ca^2+^-independent effect of BAPTA_i_, a high-affinity, rapid Ca^2+^-chelating agent. We demonstrate that BAPTA_i_ hampers the conversion of fructose-6-phosphate into fructose-1,6-bisphosphate by directly inhibiting the activity of PFKFB3, a major regulatory enzyme of this step. The inhibition of PFKFB3 by BAPTA occurred via its Ca^2+^-free form. Consequently, BAPTA_i_ strongly suppressed mTORC1 activity, thereby abrogating downstream mTORC1-controlled protein translation. BAPTA_i_ particularly affects the protein levels of short-lived proteins such as MCL-1. Consequently, BAPTA_i_ evokes cell death in MCL-1-dependent cell lines. This implies that BAPTA_i_ does not elicit a general, off-target toxicity but induces cell death via a specific Ca^2+^-independent inhibition of glycolysis. Thus, our work revealed novel intracellular crosstalk where PFKFB3 inhibition reduces MCL-1-protein levels by impeding mTORC1 activity.

Using an unbiased ^13^C tracer metabolomics approach, we found that BAPTA_i_ suppresses the conversion of fructose-6-phosphate into fructose-1,6-bisphosphate, a proximal step in glycolysis. We postulate that BAPTA_i_ evokes a direct inhibition of PFKFB3, as BAPTA inhibits the activity of purified PFKFB3 enzyme in an in vitro activity assay. The inhibition of PFKFB3 by BAPTA was characterized by an IC_50_ of 110 μM. Although this might seem a high concentration, previous work indicated that extracellular application of 10 μM BAPTA-AM results in cytosolic BAPTA concentrations of 1 to 2 mM [18], thereby enabling in cellulo inhibition of PFKFB3 by BAPTA_i_. Because of PFKFB3 inhibition, downstream pathways such as mTORC1 signaling, a major driver of protein translation, were impaired. Indeed, pharmacological PFKFB3 inhibition could mimic BAPTA_i_ and inhibit mTORC1 activity. Upon inhibition of translation, MCL-1-protein levels rapidly decline through proteasomal, not lysosomal, degradation. Moreover, similarly to BAPTA_i_, direct inhibition of PFKFB3 using AZ PFKFB3 67 too induced MCL-1 downregulation.

However, it is unlikely that BAPTA_i_ exerts such a robust blockade on the conversion of fructose-6-phosphate into fructose-1,6-bisphosphate and glycolysis solely by inhibiting PFKFB3 inhibition. This assertion is supported by two key factors: Firstly, in the in vitro kinase assay, BAPTA only partially inhibited PFKFB3 activity. Secondly, studies have demonstrated that cells lacking PFKFB3 exhibited only a partial reduction (~40–50%) in glycolytic activity [34]. These findings strongly indicate that BAPTA_i_ can impede PFK activity through multiple mechanisms beyond PFKFB3 inhibition. It is plausible that BAPTAi inhibits PFKFB1, 2 or 4, or even directly inhibits PFK1 itself. These conclusions align with our own data. On the one hand, although BAPTA only partially inhibited in vitro PFKB3 activity, BAPTAi almost completely blocked the production of fructose-1,6-bisphosphate and strongly reduced MCL-1-protein levels in cells. On the other hand, despite AZ PFKFB3 67 being a potent inhibitor of PFKFB3 (with an IC_50_ of 40 nM) and capable of completely depleting cellular fructose-2,6-bisphosphate levels (reported IC_50_ of 500 nM in A549 cells; [35]), it only partially decreased MCL-1-protein levels. These observations therefore suggest that BAPTAi may also affect the mTORC1/MCL-1 axis independently of PFKFB3. Furthermore, we speculate that the reason for AZ PFKFB 67 does not induce cell death by itself, even in MCL-1-addicted cancer cells, is that the remaining MCL-1-protein levels following AZ PFKFB3 treatment may be sufficient to sustain cell survival.

The cells’ sensitivity to BAPTA_i_ is dictated by their addiction to MCL-1. The strongest evidence comes from SVEC endothelial cells that are inherently resistant to BAPTA_i_ but become sensitive upon engineered MCL-1 addiction. In addition, not only hematological cancer cell lines but also normal B lymphocytes are sensitive to BAPTA_i_, which correlates with their sensitivity toward MCL-1 inhibition. These findings are fully in line with the requirement for MCL-1 for B lymphocyte development, maintenance and survival [36]. Of note, while AZ PFKFB3 67 did evoke a decline in MCL-1-protein levels, it was less effective than BAPTA_i_ and TF-BAPTA_i_.

MCL-1 is an important pharmacological target since it is an emergent resistance factor in tumor cells treated with BH3 mimetics, such as ABT-199 (venetoclax), ABT-263 (navitoclax) and ABT-737. The need for therapeutic agents targeting MCL-1 is therefore acute [14]. Accordingly, specific MCL-1 inhibitors have been developed, showing promising results as single agents as well as in combination with other BH3 mimetics [29, 37–40]. However, increased abundance of nontargeted BCL-2-family members is not the only resistance mechanism in tumor cells. Following prolonged treatment with venetoclax, several studies reported mutations in the hydrophobic groove of BCL-2, decreasing the affinity of venetoclax for BCL-2 and limiting its therapeutic effect [41, 42]. Although few studies have addressed this concern so far, it is plausible that a similar mechanism would occur for MCL-1 when treating tumor cells with a BH3-mimetic MCL-1 inhibitor. It could therefore be beneficial to target MCL-1 at the level of its transcription, translation, or degradation. Indeed, it has been shown that inhibition of the mTORC1 complex, and by extension *Mcl-1* translation, shows antitumor effects in lymphoma as a single agent and in combination with venetoclax [43, 44]. This is supported by our own results demonstrating that BAPTA_i_-induced inhibition of mTORC1 and consequent reduction of MCL-1-protein levels leads to cell death in MCL-1-dependent hematological cell lines. While mTORC1 aligns energy supplies with anabolic and catabolic activities in physiological conditions, in cancer cells it lies at the core of metabolic reprogramming to increase proliferation. Accordingly, mTORC1 inhibitors, so-called rapalogs, have been extensively evaluated as potential therapeutics in cancer treatments [45].

Our findings are in line with other studies demonstrating a link between glycolysis, mTORC1 activity and mRNA translation. Indeed, the team of Adams and coworkers revealed that 2-deoxyglucose (2-DG), which inhibits the first step in the glycolytic pathway, caused a decrease in p70S6K phosphorylation and an increase in 4E-BP1 activity, indicating that inhibition of glycolysis using 2-DG provoked a decrease in general translation activity [46]. In the same study, 2-DG rapidly affected steady-state MCL-1 levels, sensitizing human hematopoietic tumor cell lines to ABT-737, a nonselective BCL-2/BCL-XL-antagonizing BH3 mimetic.

PFKFB3 controls the translocation of mTORC1 to lysosomes and subsequent mTORC1 activity [47], which is in line with our data. These findings open up future research avenues since increased abundances of PFKFB3 are found in several cancers, and has been associated with cancer hallmarks such as carcinogenesis, cancer cell proliferation, drug resistance and the tumor microenvironment [32, 34].

Our work builds on a growing body of literature pointing to off-target effects of BAPTA by directly binding and impacting cellular proteins [18, 19, 48–51]. Furthermore, by inhibiting the PFKFB3/mTORC1 axis, we anticipate that BAPTA_i_ will affect several other short-lived proteins and thus will have profound effects on the cellular proteome, aspects that require further research.

Finally, whilst the bulk of data presented in this study highlights Ca^2+^-independent actions of BAPTA, we are not suggesting that Ca^2+^ chelation has no impact on MCL-1-protein levels. Indeed, EGTA_i_ modestly lowered MCL-1-protein levels (Fig. 2F) while it did not inhibit PFKFKB3 activity (Fig. 7B). Moreover, it was evident that direct inhibition PFKFB3 using AZ PFKFB3 67 compound was less potent in decreasing MCL-1-protein levels than BAPTA_i_ (Fig. 8B, D), potentially indicating a contribution of Ca^2+^ chelation to reduced MCL-1 expression independently of PFKFB3. A common argument for different biological effects being observed when comparing EGTA_i_ and BAPTA_i_ is that EGTA_i_, as a slow Ca^2+^ buffer, poorly buffers Ca^2+^ in nanodomains. Whereas BAPTA_i_, as a fast Ca^2+^ buffer, can effectively buffer Ca^2+^ in nanodomains. However, we hypothesize that this is unlikely to account for the results observed in this study since BAPTA_i_ (K_D_ of 160 nM) and TF-BAPTA_i_ (K_D_ of 65 μM) always displayed similar potencies in impeding mTORC1 and downregulating MCL-1 (Figs. 2F, 4E and 8A–D).

In conclusion, BAPTA elicits important cellular effects independent of its ability to chelate Ca^2+^. We found that BAPTA inhibited the kinase activity of the glycolytic stimulator PFKFB3, leading to impaired glycolysis. This resulted in decreased mTORC1 activity leading to dysfunctional translation. Consequently, cellular expression of MCL-1 was reduced, promoting cell death in MCL-1-dependent cancer cells.

### Reporting summary

Further information on research design is available in the Nature Research Reporting Summary linked to this article.

## Supplementary information

Reporting Summary

Adapted author list - approved by all authors

Supplementary Figures

uncropped blots

## Data Availability

All relevant data are presented in the manuscript. The full-length immunoblots are included in the Supplementary Material. The underlying data are available via KU Leuven RDR (research data repository) https://rdr.kuleuven.be/ with 10.48804/DUUFVS.
