# Supplementary material for: Intracellular BAPTA directly inhibits PFKFB3, thereby impeding mTORC1-driven Mcl-1 translation and killing MCL-1-addicted cancer cells
Source: Cell Death Dis. 2023 Sep 8;14(9):600. doi: 10.1038/s41419-023-06120-4 (PMC10491774; doi:10.1038/s41419-023-06120-4)
Supplement: Supplementary file 2 — Adapted author list - approved by all authors [file 41419_2023_6120_MOESM2_ESM.pdf]

Acceptance adapted list of authors

I fully accept the addition of Rita La Rovere to the author list.

Flore Sneyers

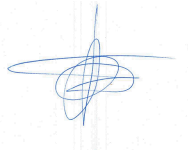

Geert Bultynck

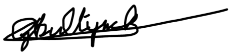

Arnout Voet
Thu 8/17/2023 10:33 AM

To: ☐ Flore Sneyers;

i accept the addition of Rita La Rovere to the authorlist

Kindest Regards,
Arnout Voet

Associate-Professor
Laboratory of biomolecular modelling and design
Celestijnenlaan 200G
3001 Heverlee
Belgium

Tel: +3216324496
https://www.chem.kuleuven.be/lbmd/index.html
@kullbmd

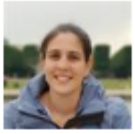

Kirsten Welkenhuyzen
Thu 8/17/2023 10:36 AM

Hi Flore,

I accept that Rita La Rovere is added to the list of authors!

Kind regards
Kirsten Welkenhuyzen

Bart Ghesquière
Thu 8/17/2023 10:44 AM

To: ☐ Flore Sneyers;

Cc: ☐ Geert Bultynck;

Hi Flore,

I accept the addition of Rita La Rovere to the list of authors of the BAPTA paper,

Best,

Bart

Prof Dr Bart Ghesquiere

Head of Metabolomics Expertise Center

KU Leuven Full Professor
Laboratory of Applied Mass Spectrometry
Department of Cellular and Molecular Medicine

VIB Senior Expert Technologist
VIB Center for Cancer Biology

Gasthuisberg O&N4
Herestraat 49 bus 912
B-3000 Leuven
Belgium

+3216322733
email: Bart.Ghesquiere@kuleuven.be

Mieke Dewerchin
Thu 8/17/2023 10:49 AM

To: ☐ Flore Sneyers;

Cc: ☐ Geert Bultynck;

Dear Flore, dear Geert,

Congratulations with the acceptance of the paper.

I hereby confirm that I accept the addition of Rita La Rovere to the list of authors.

Kind regards,
Mieke Dewerhin

****
Mieke Dewerchin, PhD
Professor
Laboratory of Angiogenesis and Vascular Metabolism
VIB-KU Leuven Center for Cancer Biology (CCB)
Department of Oncology, KU Leuven
Campus Gasthuisberg O&N4, Bldg 404-24
Herestraat 49 - box 912
B-3000 Leuven
Belgium
Phone: +32-16-37.31.97
E-mail: mieke.dewerchin@kuleuven.be
****

Guy Eelen <guy.eelen@droialabs.com>
Thu 8/17/2023 11:27 AM

To: ☐ Flore Sneyers;

Dear Flore,

Good to hear that your paper was accepted !

I hereby confirm that I agree with the addition of Rita La Rovere to the list of authors.

Best regards,

Guy

____

Guy Eelen, PhD
Director Biology
Metaptys NV / Droia Labs
Arenberg Accelerator - Gaston Geenslaan 3, 1st floor, Unit 01.200 – 3001 Leuven, Belgium
Office phone: +32 16 88 29 00

Martin.Bootman <martin.bootman@open.ac.uk>
Thu 8/17/2023 11:21 AM

Show all 11 recipients

To: ☐ Flore Sneyers; Martijn Kerkhofs; Femke Speelman-Rooms; Rita La Rovere; ☐ Kirsten Welkenh
Ahmed Shemy; ☐ Arnout Voet; ...

Hi Flore,

I completely accept the inclusion of **Rita La Rovere** as a co-author on the paper.

Best wishes,

Martin

[illegible]

Stephen Tait <Stephen.Tait@glasgow.ac.uk>

Thu 8/17/2023 12:27 PM

To: Flore Sneyers;

Dear Flore

I accept the addition of Rita La Rovere.

Best wishes
Stephen

Femke Speelman-Rooms
Thu 8/17/2023 2:47 PM

To: ☐ Flore Sneyers;

Hi Flore,

I completely accept the inclusion of Rita La Rovere as a co-author on the paper.

Best wishes,

Femke

REPLY

KERKKOFS MARTIJN <martijn.kerkhofs@univ-lyon1.fr>
Fri 8/18/2023 9:34 PM

To: ☐ Flore Sneyers;

Dear Flore,

I accept the addition of Rita La Rovere to the author list.

Kind regards,

Martijn

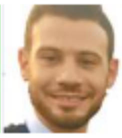

Ahmed Shemy
Fri 8/18/2023 2:39 PM

To: ☐ Flore Sneyers;

Hi Flore,

I completely accept the inclusion of **Rita La Rovere** as a co-author on the paper.

Best wishes,

Ahmed.
