# Supplementary material for: Intracellular BAPTA directly inhibits PFKFB3, thereby impeding mTORC1-driven Mcl-1 translation and killing MCL-1-addicted cancer cells
Source: Cell Death Dis. 2023 Sep 8;14(9):600. doi: 10.1038/s41419-023-06120-4 (PMC10491774; doi:10.1038/s41419-023-06120-4)
Supplement: Supplementary file 3 — Supplementary Figures [file 41419_2023_6120_MOESM3_ESM.pdf]

| **Compound** | **Molecular structure** | **Dissociation constant ($K_D$) for $Ca^{2+}$ binding to the free acid form** |
|---|---|---|
| EGTA-AM | 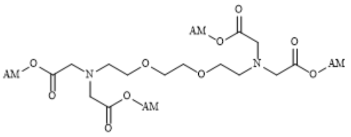 | 60.5 nM<br>At pH 7.4 and 20°C |
| Tetrafluoro-BAPTA-AM<br>(TF-BAPTA-AM) | 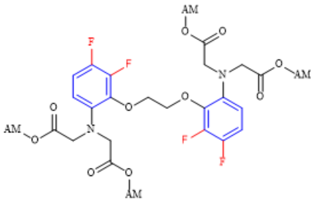 | 65 µM |
| Difluoro-BAPTA-AM<br>(DF-BAPTA-AM) | 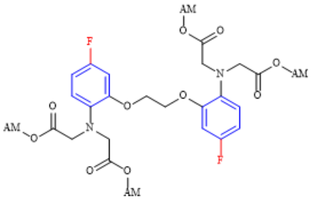 | 635 nM |
| BAPTA-AM | 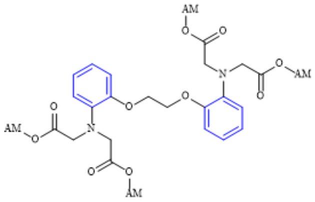 | 160 nM |
| Dimethyl-BAPTA-AM<br>(DM-BAPTA-AM) | 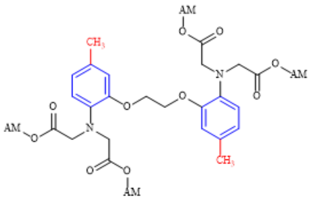 | 40 nM |

**Supplementary table 1: $Ca^{2+}$-chelating compounds and their corresponding $K_D$ values for $Ca^{2+}$ binding of the free-acid form.**

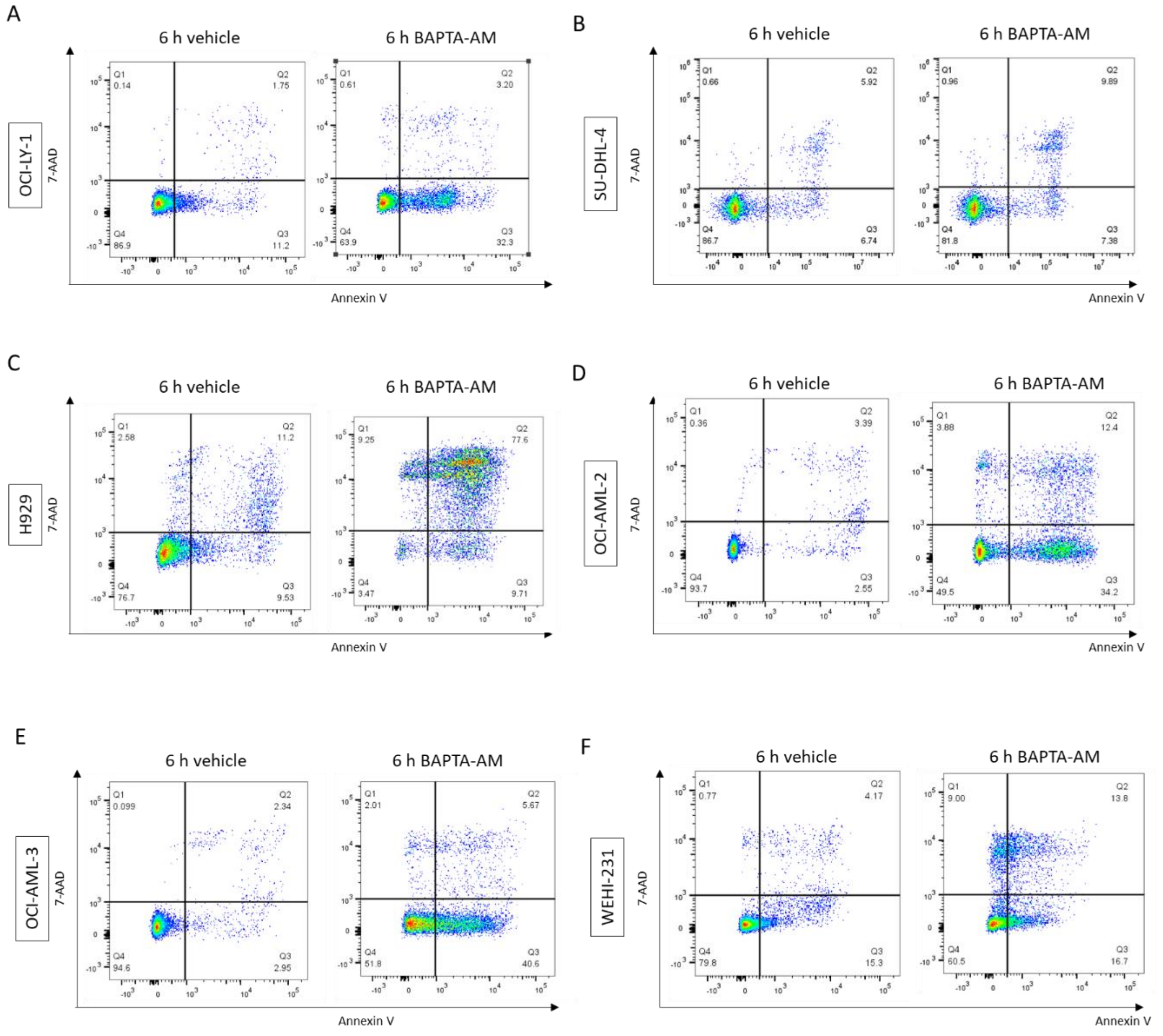


**Supplementary figure 1**

Representative graphs of apoptosis of OCI-LY-1 (A), SU-DHL-4 (B), H929 (C), OCI-AML-2 (D), OCI-AML-3 (E) and WEHI-231 (F) cells at different time points after the addition of vehicle (DMSO) or 10 µM BAPTA-AM. Cells were stained with annexin V-FITC and 7-AAD, and the apoptotic fraction was identified as annexin V-positive cells (N = 3).

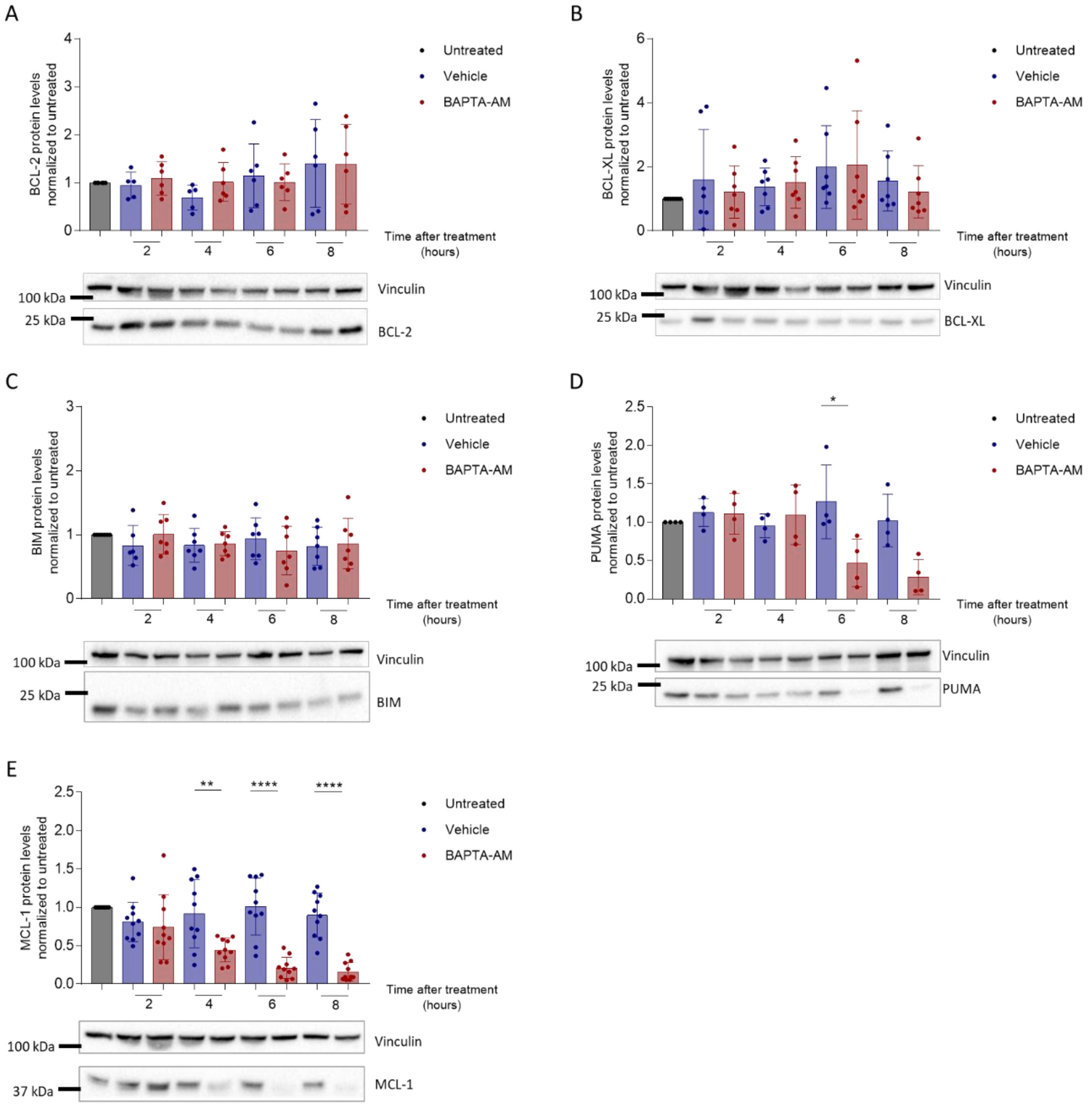


**Supplementary figure 2**

Representative western blot and statistical analysis of BCL-2 (A, N =6), BCL-XL (B, N =7), BIM (C, N =7), PUMA (D, N =3) and MCL-1 (E, N =10) levels normalized to untreated in response to 10 µM of vehicle (dark blue) and BAPTA-AM (red) after 2, 4, 6, and 8 hours in SU-DHL-4 cells. Vinculin was included as a loading control. Data are represented as the average ± S.D. Statistically significant differences were determined with a paired two-tailed Student's t-test. Differences were considered significant when $p < 0.05$. (** $p < 0.01$; *** $p < 0.001$).

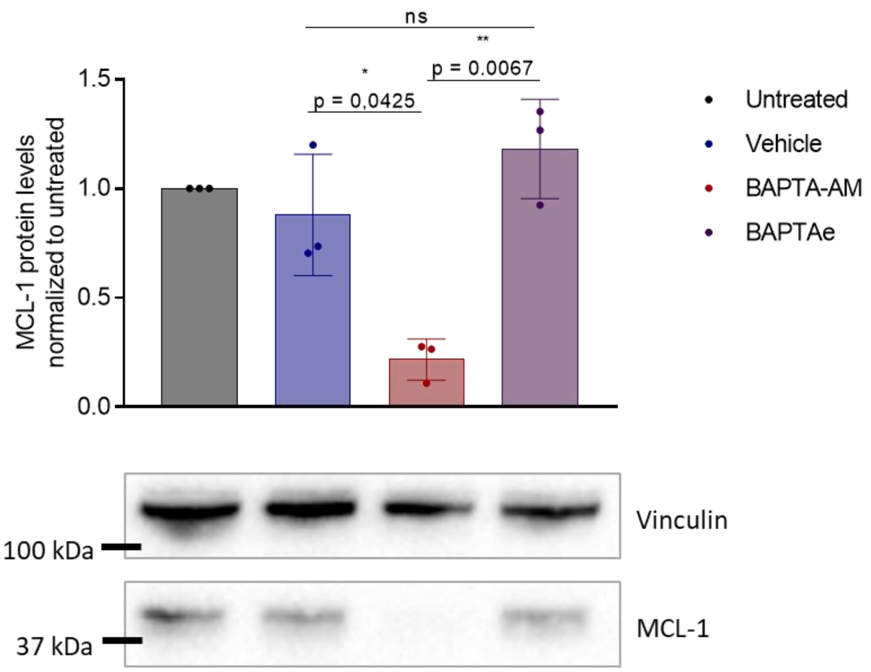


**Supplementary figure 3**

Representative western blot and densitometric analysis of MCL-1-protein levels normalized to untreated in response to a 6 h treatment with of vehicle (dark blue), 10 µM BAPTA-AM (red) or extracellular BAPTA (purple) in OCI-LY-1 cells. Data are represented as the average ± S.D. (N = 3). Statistically significant differences were determined with a paired one-way ANOVA test. Differences were considered significant when $p < 0.05$. (** $p < 0.01$; *** $p < 0.001$).

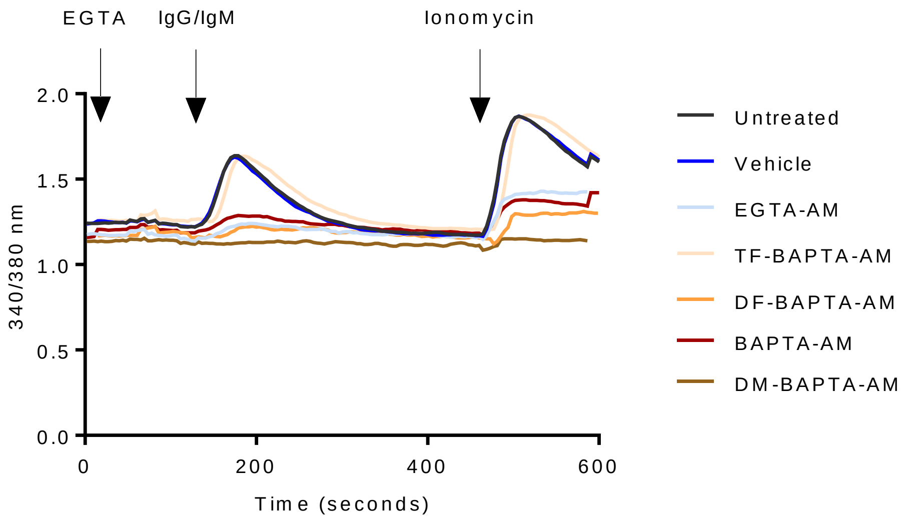


**Supplementary figure 4**

Mean cytosolic $Ca^{2+}$ measurements using Fura-2-AM in OCI-LY-1 cells after 30 minutes of pretreatment with 10 µM of vehicle (dark blue, N = 8), EGTA-AM (light blue, N = 4), TF-BAPTA-AM (yellow, N = 4), DF-BAPTA-AM (orange, N = 4), BAPTA-AM (red, N = 4) and DM-BAPTA-AM (brown, N = 4). Extracellular EGTA was used to buffer extracellular $Ca^{2+}$, IgG/IgM and ionomycin trigger $Ca^{2+}$ release from the ER and the extracellular space, respectively.

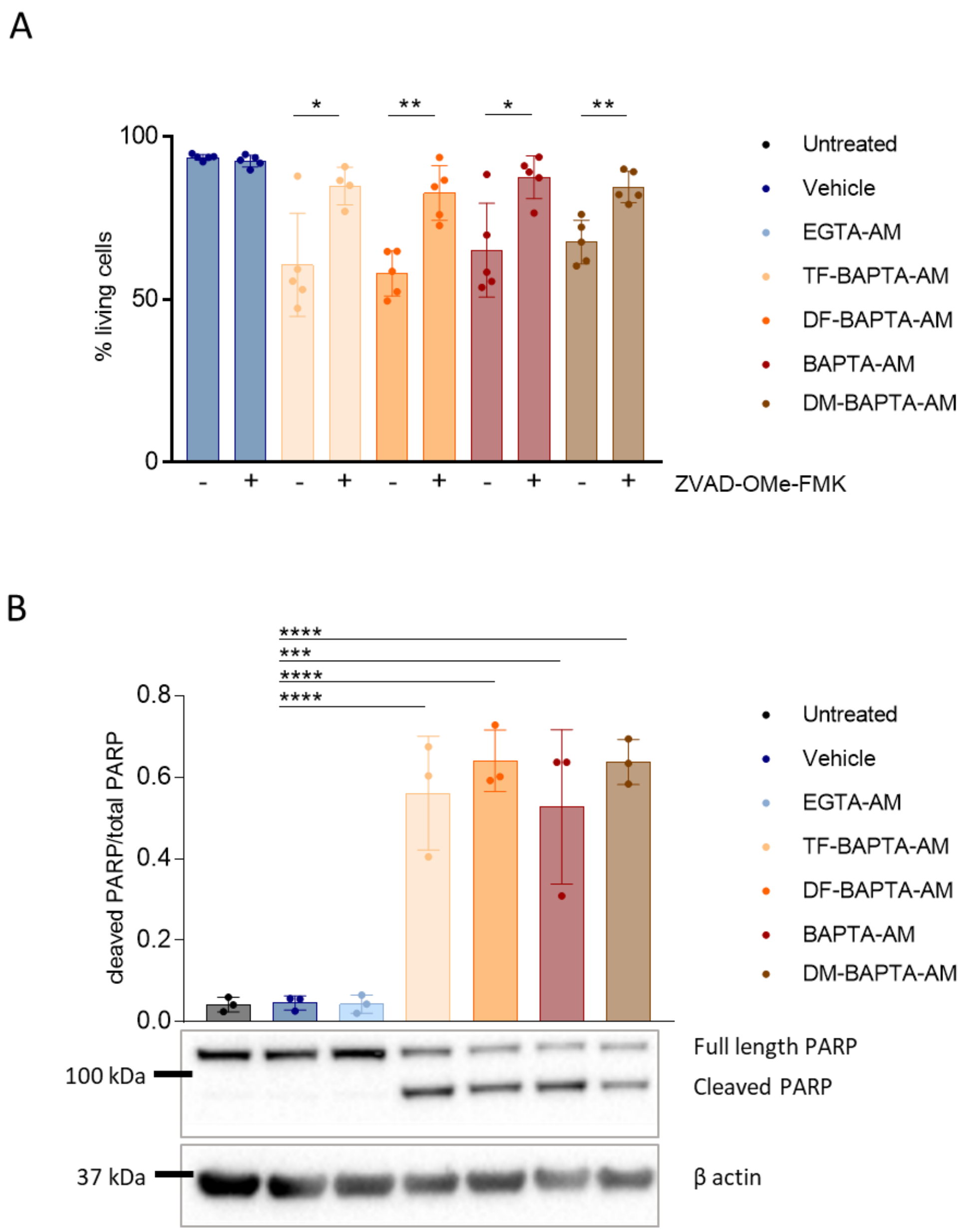


**Supplementary figure 5**

(A) Quantitative analysis of apoptosis in OCI-LY-1 in response to 6 h treatment with vehicle (dark blue), 10 µM of TF-BAPTA-AM (yellow), DF-BAPTA-AM (orange), BAPTA-AM (red) and DM-BAPTA-AM (brown) and co-treatment for 6 h with 10 µM ZVAD-OMe-FMK. Cells were stained with annexin V-FITC and 7-AAD and the apoptotic fraction was identified as annexin V positive cells. Data are represented as the average ± S.D.; N = 5. Statistically significant differences were determined with a paired two-tailed Student's t-test. Differences were considered significant when $p < 0.05$. (** $p < 0.01$; *** $p < 0.001$). (B) Densitometric analysis and representative western blot of PARP cleavage/total PARP protein levels normalized to untreated in response to vehicle (dark blue), 10 µM of EGTA-AM (light blue), TF-BAPTA-AM (yellow), DF-BAPTA-AM (orange), BAPTA-AM (red) and DM-BAPTA-AM (brown). Β actin was used as a loading control. Data are represented as the average ± S.D.; N = 3. Statistically significant differences were determined with a one-way ANOVA. Differences were considered significant when $p < 0.05$. (** $p < 0.01$; *** $p < 0.001$).

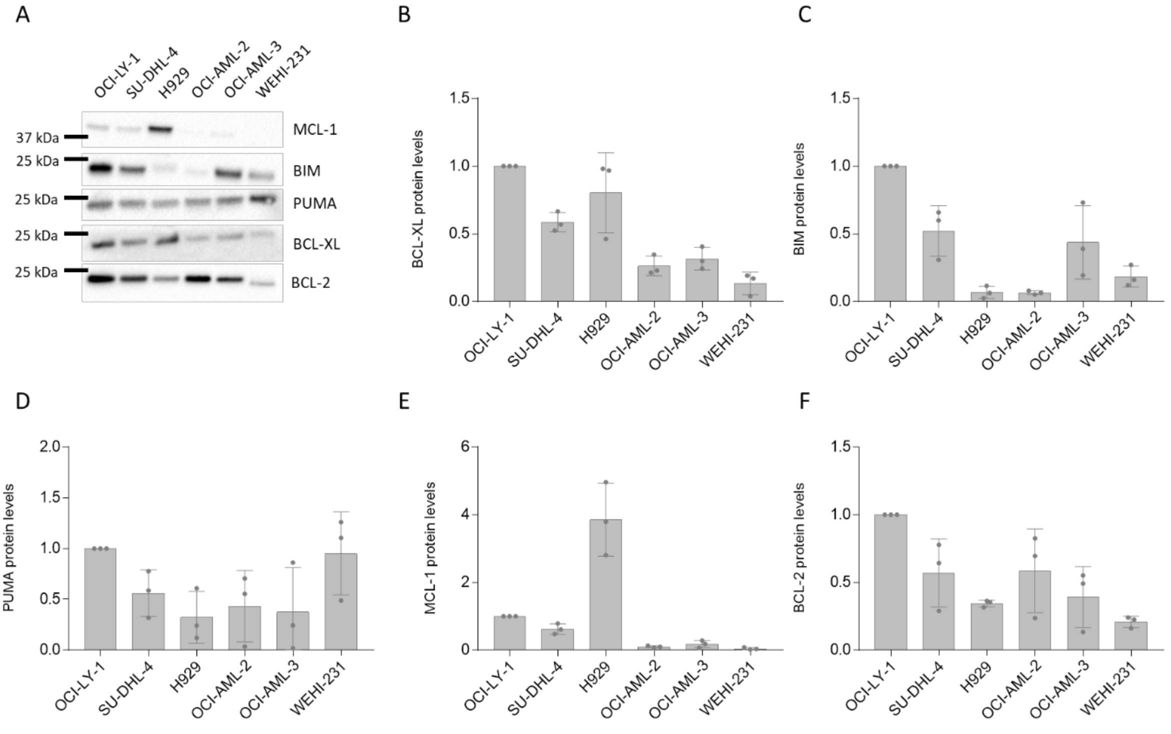


**Supplementary figure 6**

Representative western blots (A) and densitometric analysis of BCL-XL- (B), BIM- (C), PUMA- (D), MCL-1- (E) and BCL-2- (F) protein levels normalized to OCI-LY-1 expression levels of each protein under untreated conditions. Data are represented as the average ± S.D. (N = 3).

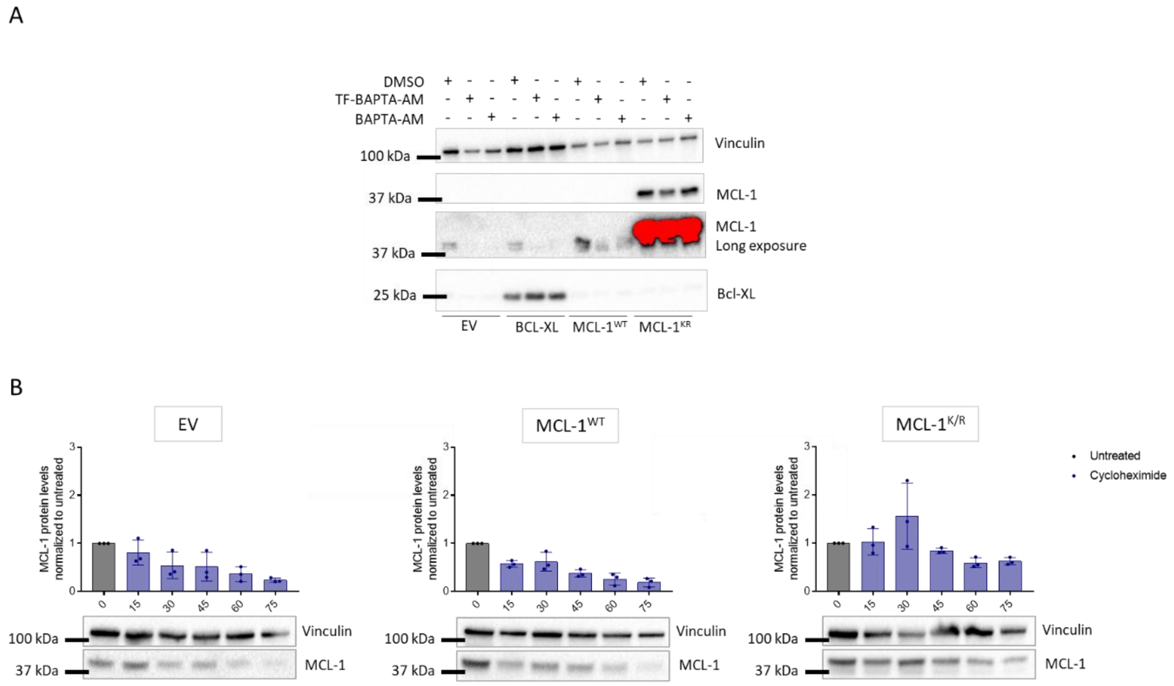


**Supplementary figure 7**

(A) OCI-LY-1 cells were transfected with an empty vector (EV), a BCL-XL-overexpressing plasmid, a wild-type (WT) MCL-1-overexpressing plasmid or a plasmid overexpressing a nondegradable KR mutant of MCL-1. Representative western blot of MCL-1 and BCL-XL levels 24 hours after transfection of OCI-LY-1 cells treated for 6 hours with vehicle (dark blue), 10 µM TF-BAPTA-AM (yellow) or BAPTA-AM (red) (N = 5). (B) OCI-LY-1 cells were transfected with an empty vector (EV), a wild-type (WT) MCL-1-overexpressing plasmid or a plasmid overexpressing a nondegradable KR mutant of MCL-1. Twenty-four hours post transfection, OCI-LY-1 cells were treated with 20 ng/mL cycloheximide for 15-30-45-60 and 75 minutes. Representative western blot and densitometric analysis of MCL-1 levels are shown for each transfection condition. MCL-1-protein levels were normalized to loading control (vinculin) and to untreated transfected conditions. Data are represented as the average ± S.D. (N = 3).

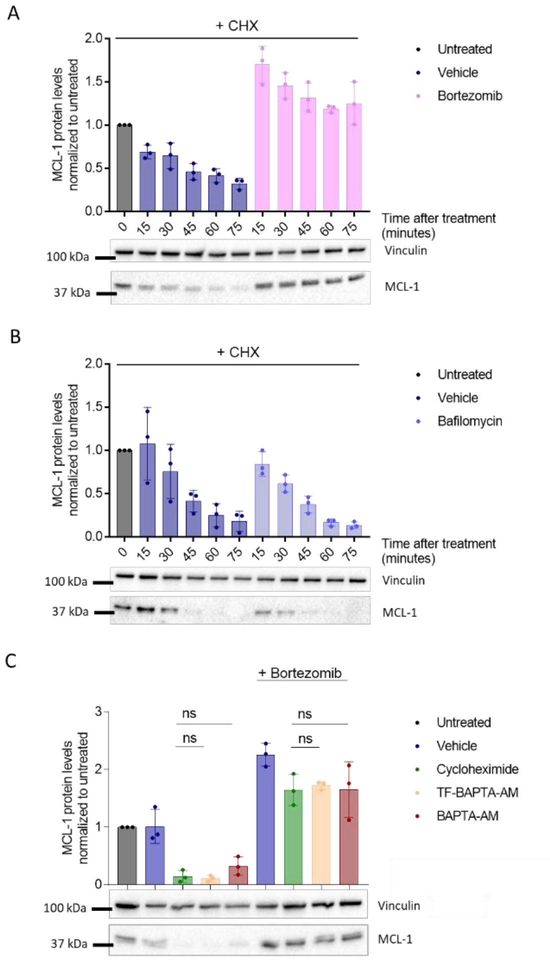


**Supplementary figure 8**

(A) Representative western blot and densitometric analysis of MCL-1-protein levels after 0, 15, 30, 45, 60 and 75 minutes of vehicle (dark blue) or 5 µM bortezomib (pink) in OCI-LY-1 cells. Prior to this (15 minutes), the cells were pretreated with 20 µg/mL cycloheximide. Quantified MCL-1-protein levels were normalized to the loading control (vinculin) and calculated relative to the untreated condition. Data are represented as the average ± S.D. (N = 3). Statistically significant differences were determined with a paired two-tailed Student's t-test. Differences were considered significant when $p < 0.05$. (** $p < 0.01$; *** $p < 0.001$). (B) Representative western blot and densitometric analysis of MCL-1-protein levels after 0, 15, 30, 45, 60 and 75 minutes of vehicle (dark blue) or 100 nM bafilomycin (purple) in OCI-LY-1 cells. Prior to this, the cells were pretreated with 20 µg/mL cycloheximide. Quantified MCL-1 levels were normalized to the loading control (vinculin) and calculated relative to the untreated condition. Data are represented as the average ± S.D. (N = 3). Statistically significant differences were determined with a paired two-tailed Student's t-test. Differences were considered significant when $p < 0.05$. (** $p < 0.01$; *** $p < 0.001$).(C) Representative western blot and densitometric analysis of MCL-1-protein levels after 3 hours of treatment with vehicle (dark blue), 20 µg/mL cycloheximide (green), 10 µM TF-BAPTA-AM or 10 µM BAPTA-AM in OCI-LY-1 cells. Prior to this, cells either received no pretreatment or were pretreated with 5 µM bortezomib. Quantified MCL-1-protein levels were normalized to the loading control (vinculin) and calculated relative to the untreated condition. Data are represented as the average ± S.D. (N = 3). Statistically significant differences were determined with a paired ANOVA test. Differences were considered significant when $p < 0.05$. (** $p < 0.01$; *** $p < 0.001$).

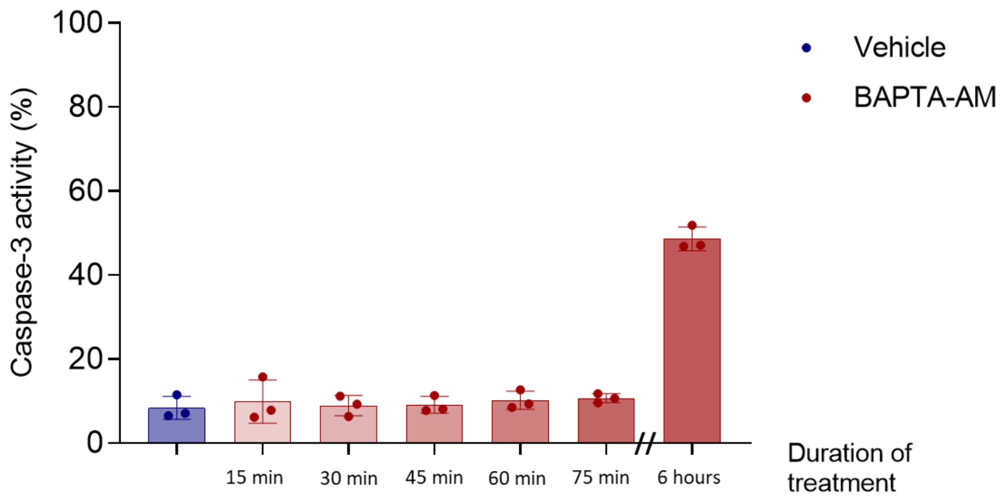


**Supplementary figure 9**

Data are represented as the average ± S.D. (N = 3). Statistically significant differences were determined with a paired ANOVA test. Differences were considered significant when $p < 0.05$. (** $p < 0.01$; *** $p < 0.001$).

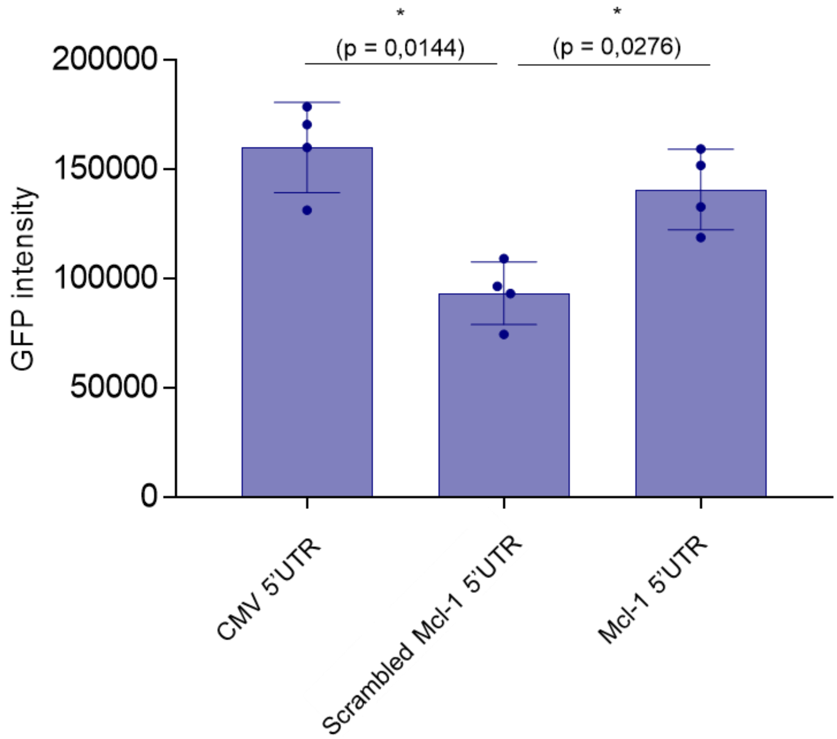


**Supplementary figure 10**

A pcDNA3.1 reporter vector was inserted with the original (*Mcl-1*) or a scrambled (control, CTRL) 5'UTR region of *Mcl-1*. A construct with the original CMV 5' UTR was included as an additional control. OCI-LY-1 cells were subsequently electroporated with one of the constructs. GFP intensity levels were measured after 24 hours of transfection with the aforementioned constructs and vehicle treatment. Data are represented as the average ± S.D. (N = 4) Statistically significant differences were determined with a paired one-way ANOVA test. Differences were considered significant when $p < 0.05$. (** $p < 0.01$; *** $p < 0.001$).

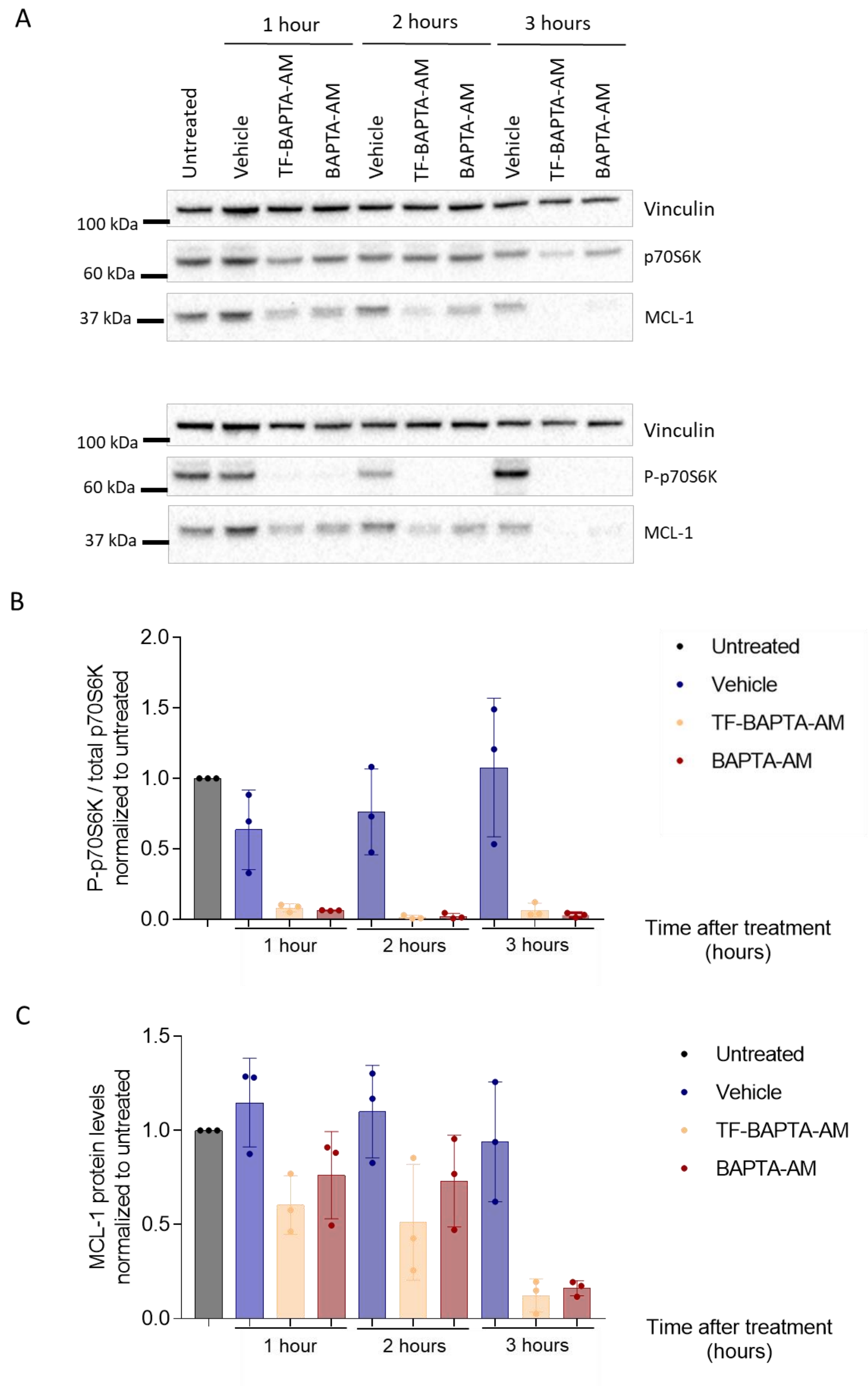


**Supplementary figure 11**

Representative western (A) blot and densitometric analysis (B and C) of P-p706K/total p70S6K (B) and MCL-1-(C) protein levels after 1, 2 and 3 hours of vehicle (dark blue), 10 uM TF-BAPTA-AM (yellow) or BAPTA-AM (red) treatment. Quantified P-p706K/total p70S6K and MCL-1 levels were normalized to the loading control (vinculin) and calculated relative to the untreated condition. Data are represented as the average ± S.D. (N = 3). Statistically significant differences were determined with a paired one-way ANOVA test. Differences were considered significant when $p < 0.05$. (** $p < 0.01$; *** $p < 0.001$).

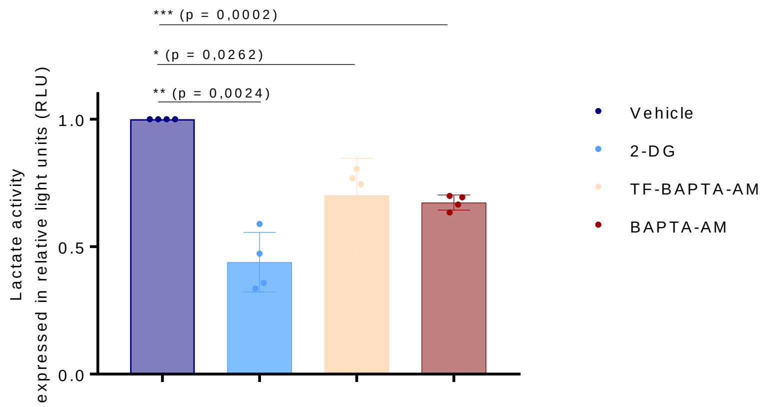


**Supplementary figure 12**

OCI-LY-1 cells were pretreated for 1 h with 10 µM of vehicle (dark blue), 10 mM 2-DG (bright blue), 10 µM TF-BAPTA-AM (yellow) and 10 µM BAPTA-AM (red). Extracellular lactate was measured and the amount of extracellular lactate correlates with absorption levels expressed in relative light units (RLU) normalized to vehicle treatment. Each condition, was measured in threefold. Data are presented as the average ± S.E.M. (N = 4). Statistically significant differences were determined with a paired one-way ANOVA test. Differences were considered significant when $p < 0.05$. (** $p < 0.01$; *** $p < 0.001$).

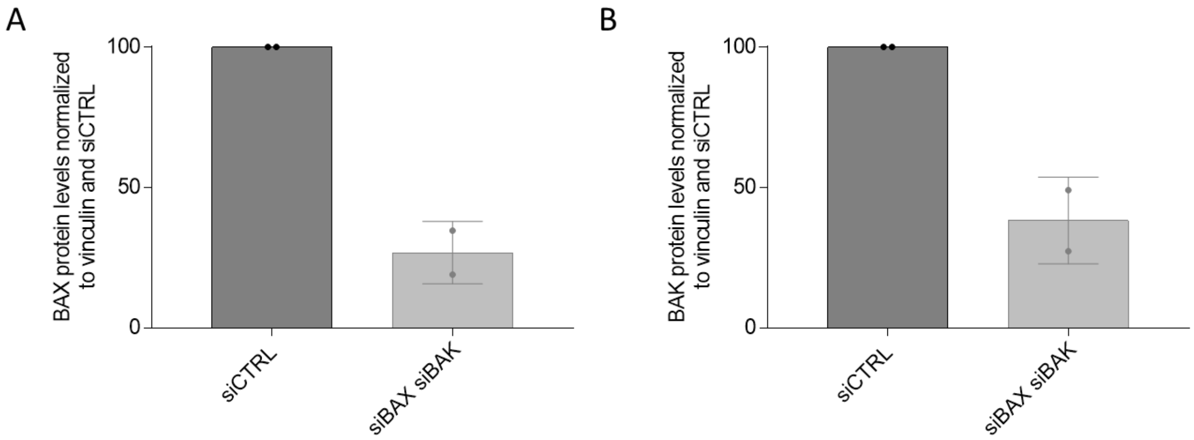


**Supplementary figure 13**

Densitometric analysis of BAX- (A) and BAK- (B) protein levels after transfection with siCTRL (dark grey) or siBAK + siBAX (light grey).

AZ PFKFB3 67

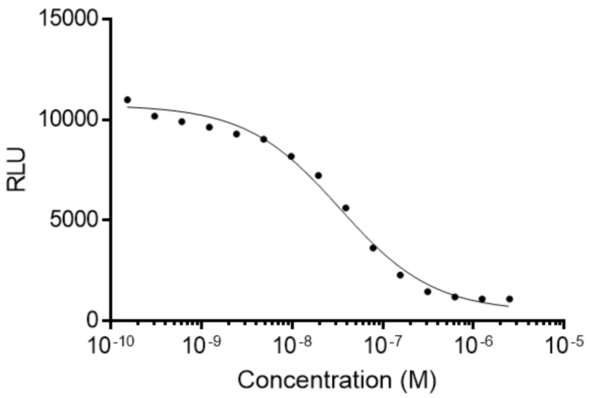


**Supplementary figure 14**

*In vitro* measurement of PFKFB3 activity in the presence of increasing concentrations of AZ PFKFB3 67.

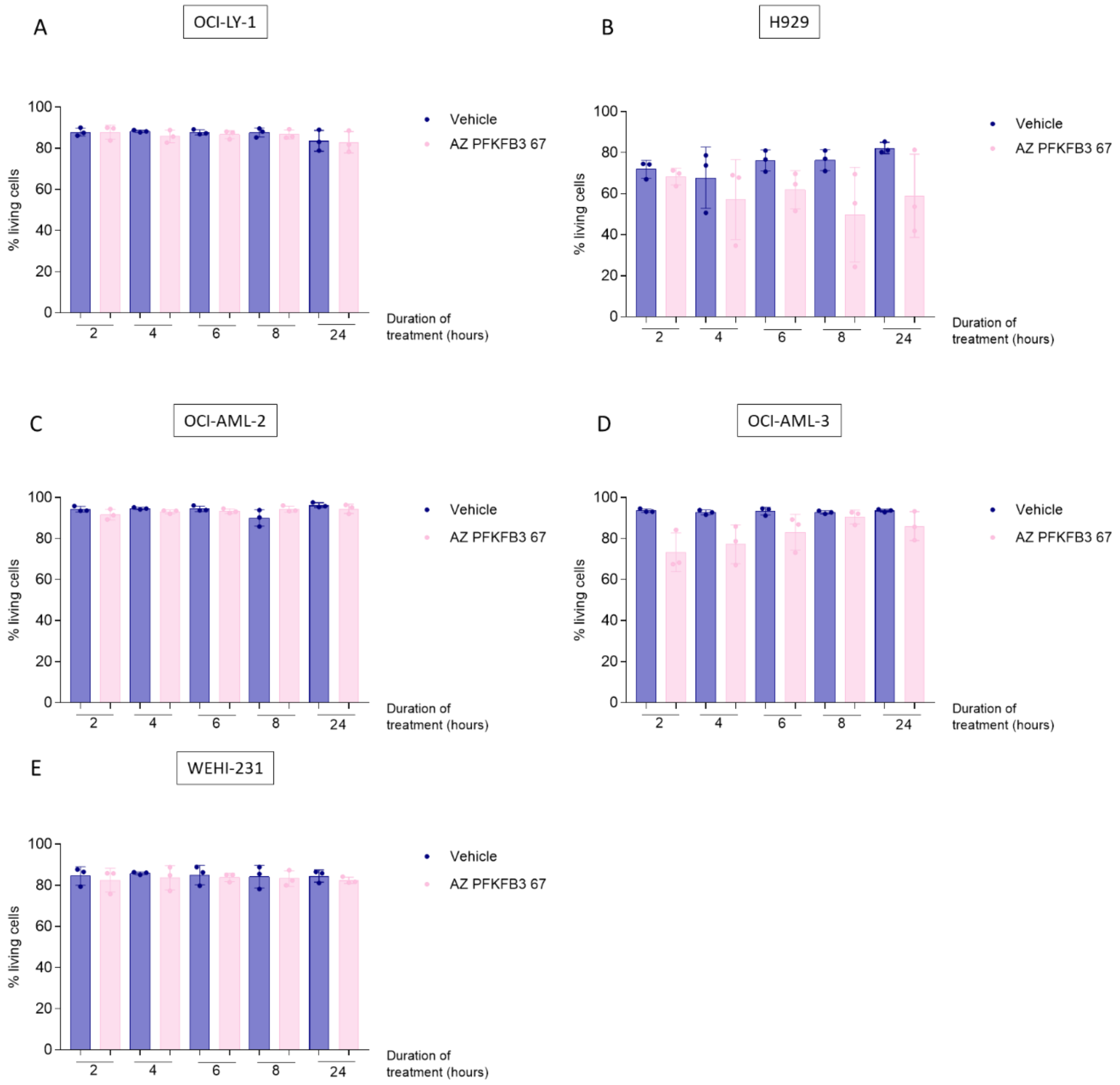


**Supplementary figure 15**

Quantitative analysis of apoptosis of OCI-LY-1 (A), H929 (B), OCI-AML-2 (C), OCI-AML-3 (D) and WEHI-231 (E) cells at different time points after the addition of vehicle (DMSO, blue) or 20 µM AZ PFKFB3 67 (pink). Cells were stained with annexin V-FITC and 7-AAD, and the apoptotic fraction was identified as annexin V-positive cells. Data are represented as the average ± S.D. (N = 3). Statistical significance of differences was determined with a paired t-test. Differences were considered significant when $p < 0.05$. (** $p < 0.01$; *** $p < 0.001$).
