# Supplementary material for: Intracellular BAPTA directly inhibits PFKFB3, thereby impeding mTORC1-driven Mcl-1 translation and killing MCL-1-addicted cancer cells
Source: Cell Death Dis. 2023 Sep 8;14(9):600. doi: 10.1038/s41419-023-06120-4 (PMC10491774; doi:10.1038/s41419-023-06120-4)
Supplement: Supplementary file 4 — uncropped blots [file 41419_2023_6120_MOESM4_ESM.pdf]

Sneyers F et al

Full-length blots

2A

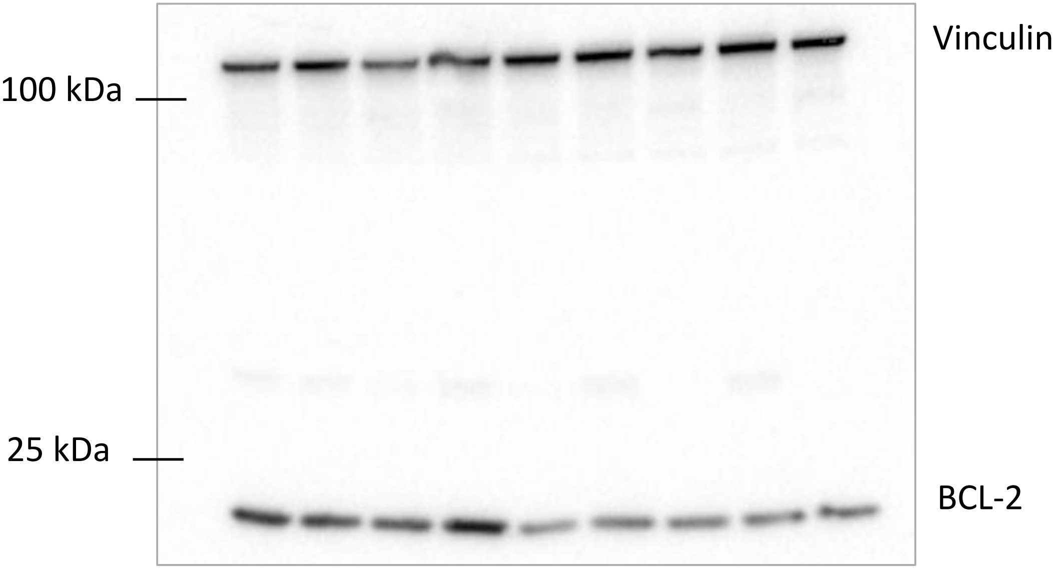

2B

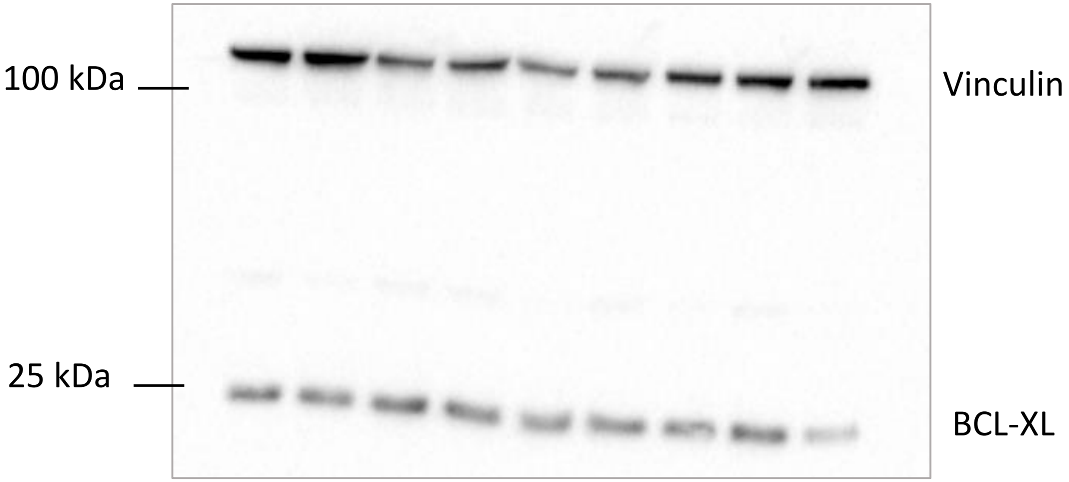

2C

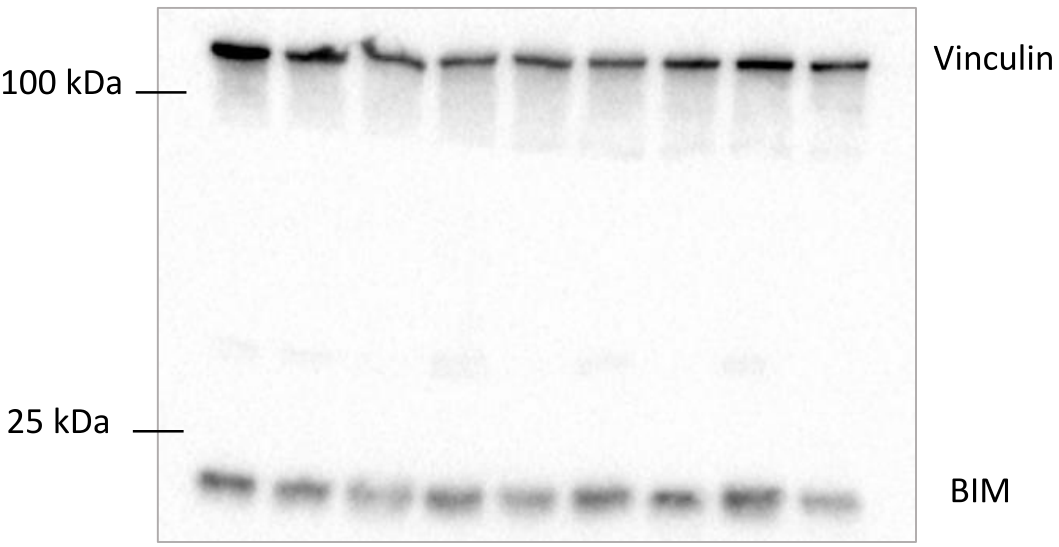

2D

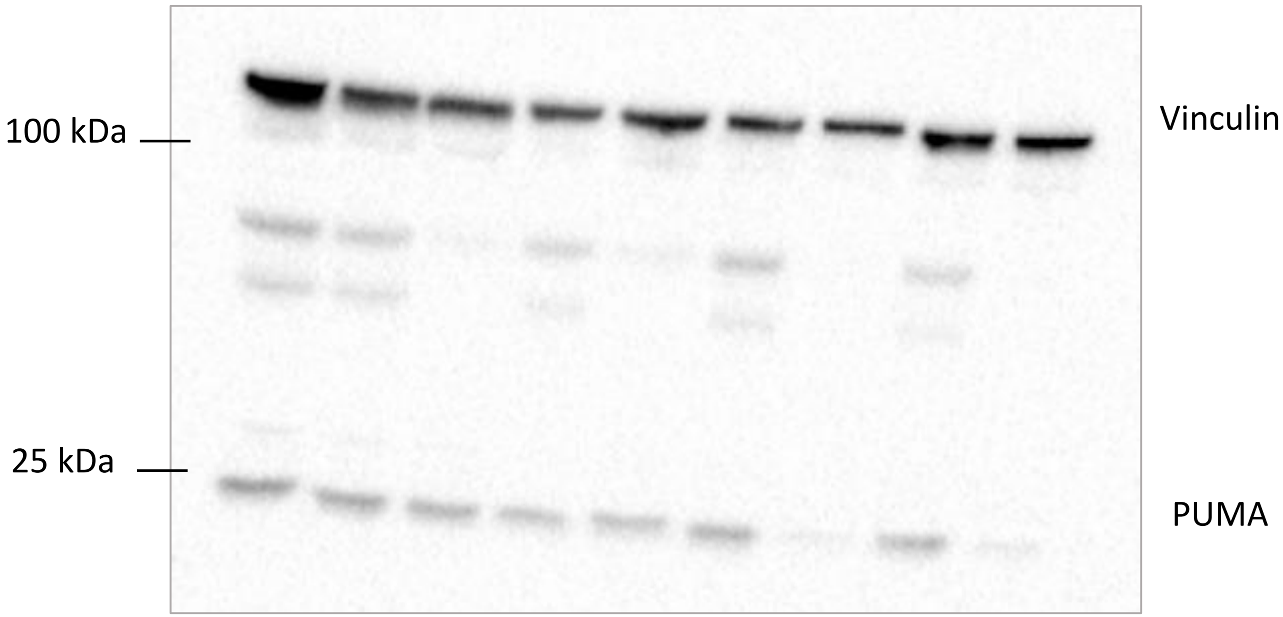

2E

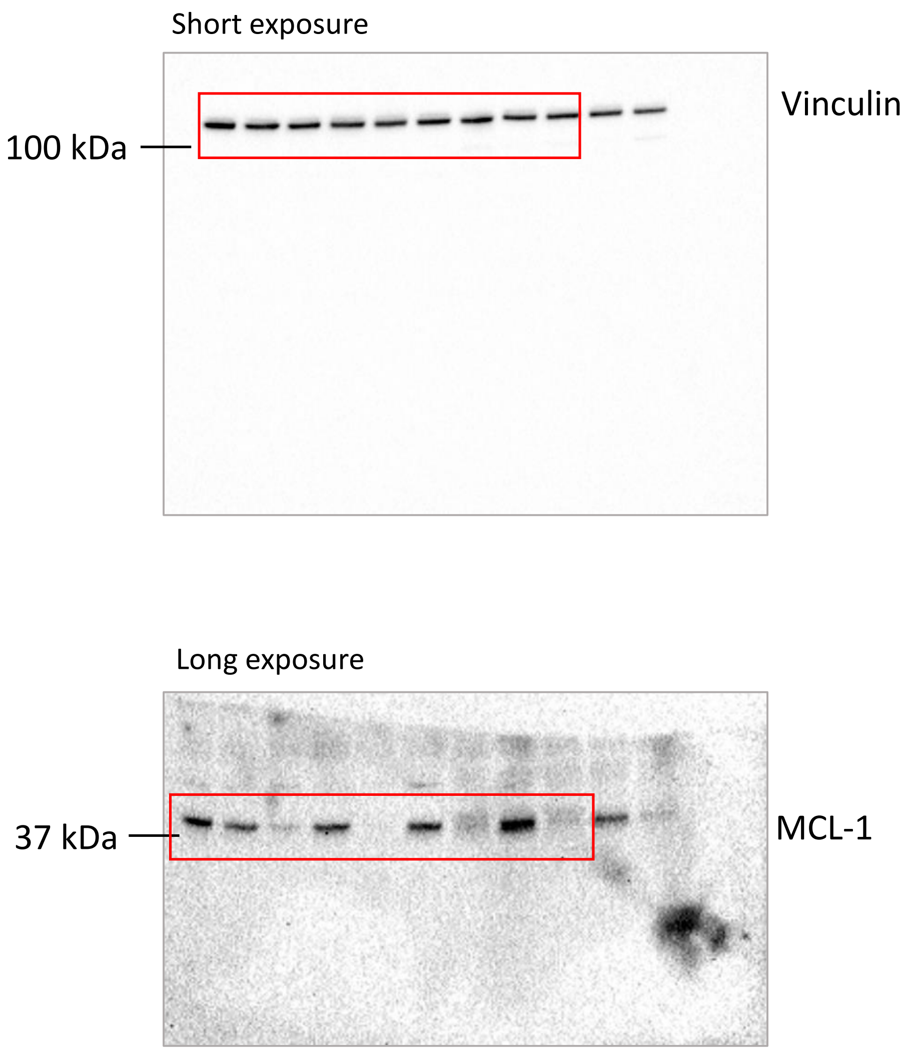

Short exposure
100 kDa
Vinculin
Long exposure
37 kDa
MCL-1

2F

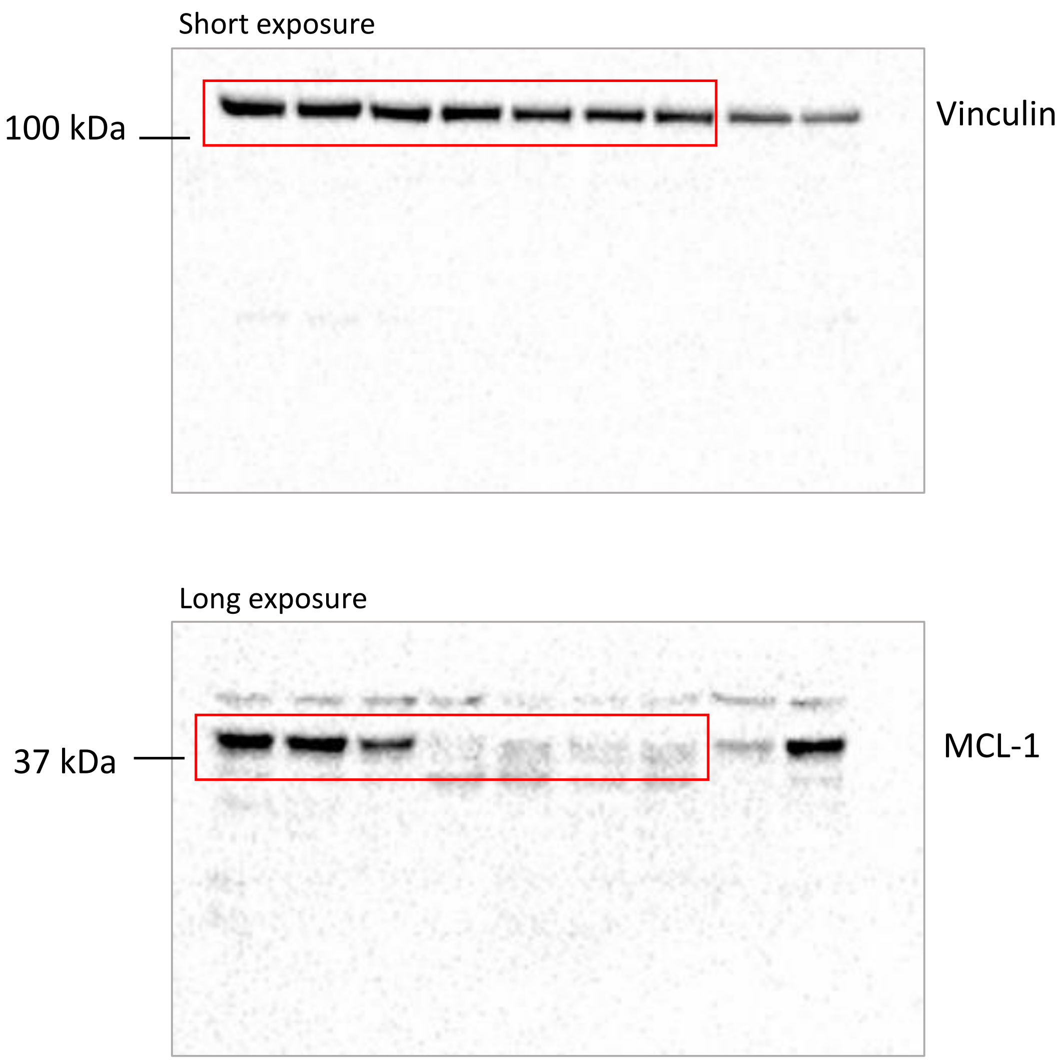

Short exposure
100 kDa
Vinculin
Long exposure
37 kDa
MCL-1

4B

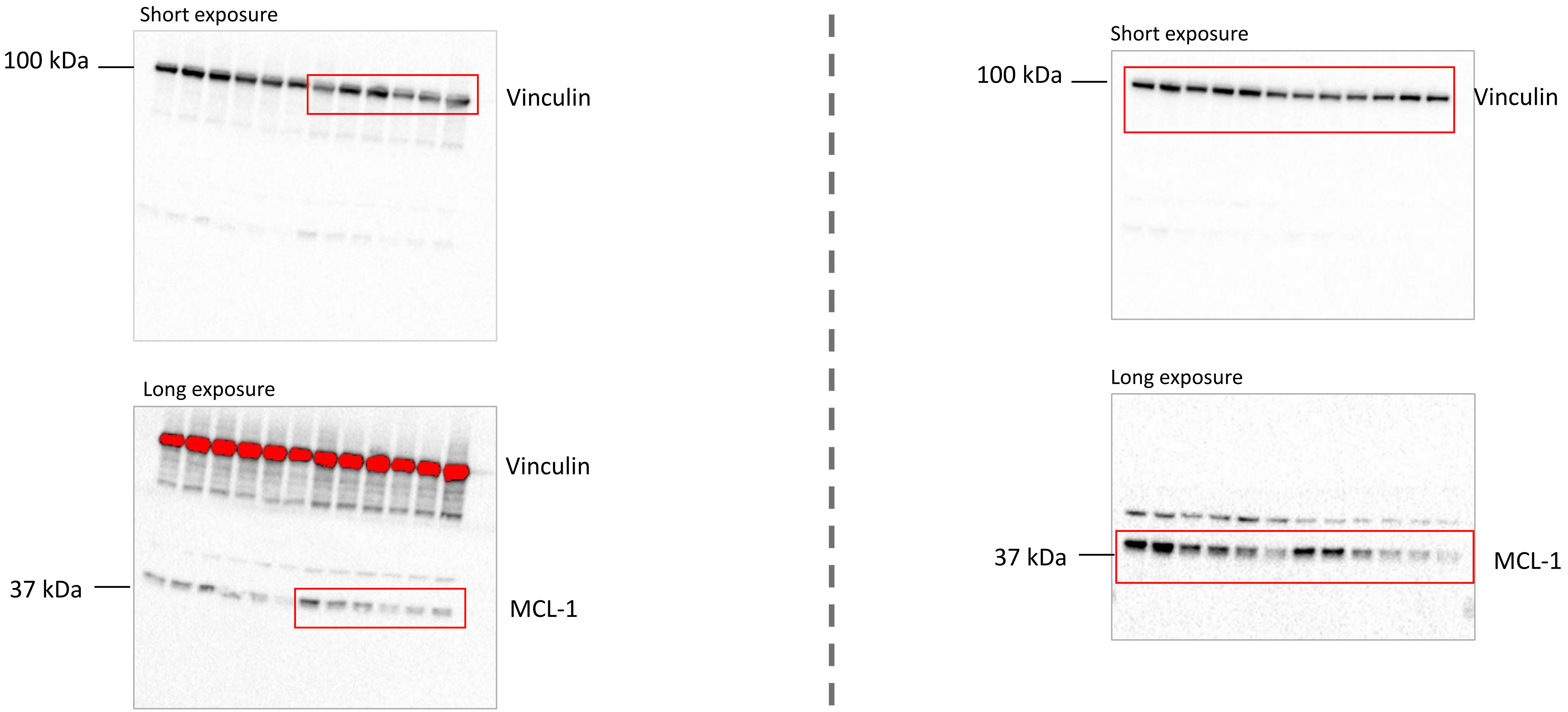

Short exposure
100 kDa
Vinculin
Long exposure
Vinculin
37 kDa
MCL-1
Short exposure
100 kDa
Vinculin
Long exposure
37 kDa
MCL-1

4E

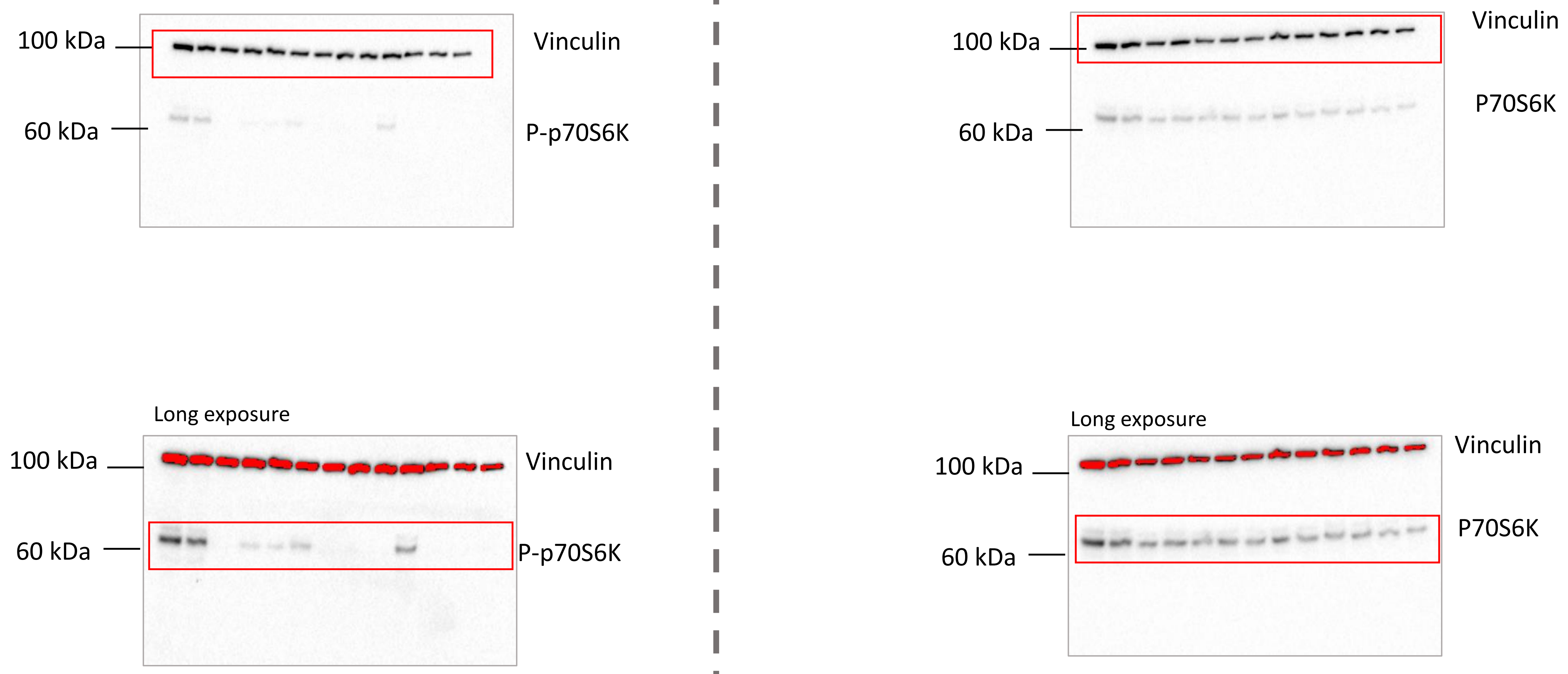
100 kDa
Vinculin
60 kDa
P-p70S6K
Vinculin
100 kDa
P70S6K
60 kDa
Long exposure
100 kDa
Vinculin
60 kDa
P-p70S6K
Long exposure
Vinculin
100 kDa
P70S6K
60 kDa

4G

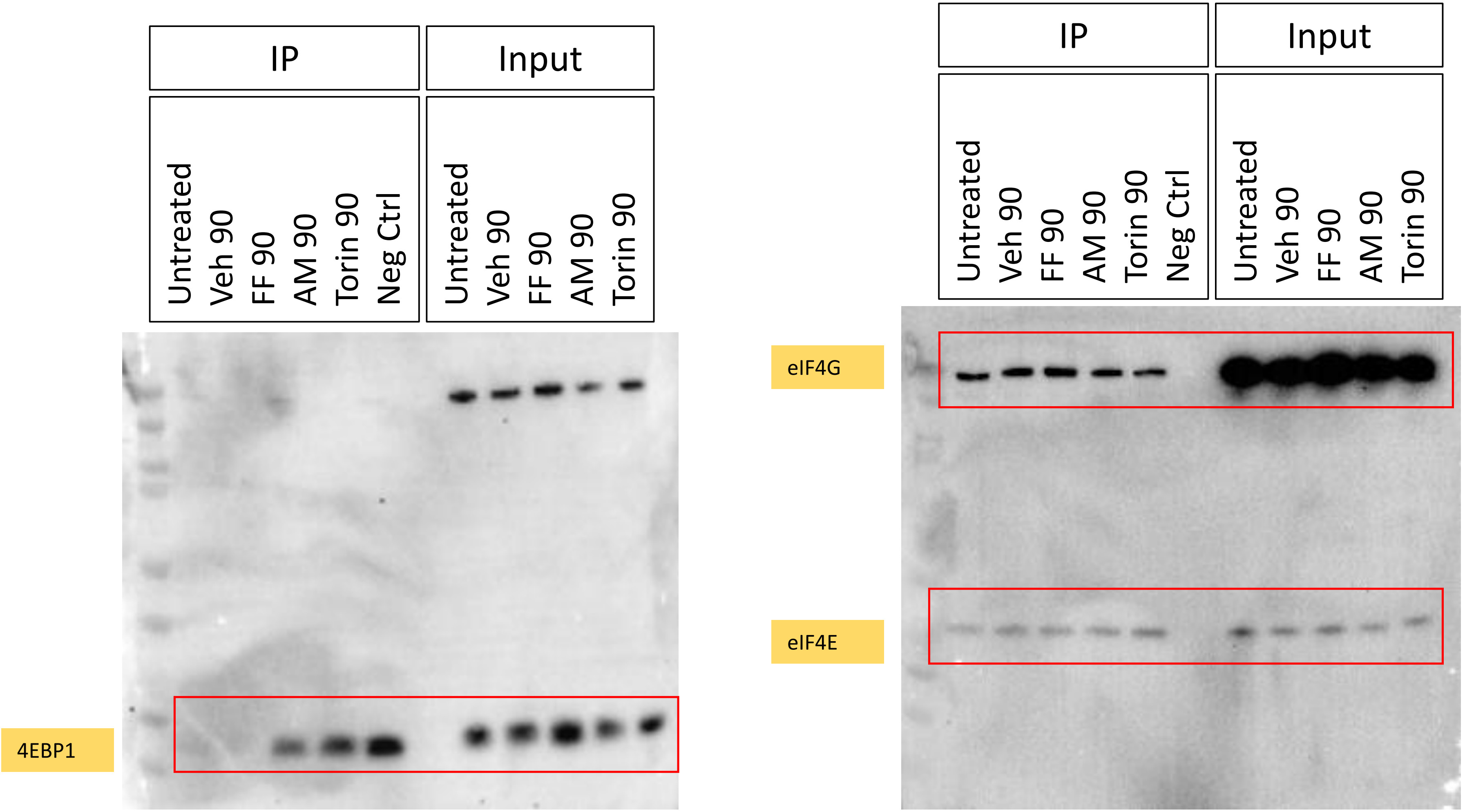
IP
Input
Untreated
Veh 90
FF 90
AM 90
Torin 90
Neg Ctrl
Untreated
Veh 90
FF 90
AM 90
Torin 90
4EBP1
IP
Input
Untreated
Veh 90
FF 90
AM 90
Torin 90
Neg Ctrl
Untreated
Veh 90
FF 90
AM 90
Torin 90
eIF4G
eIF4E

4G

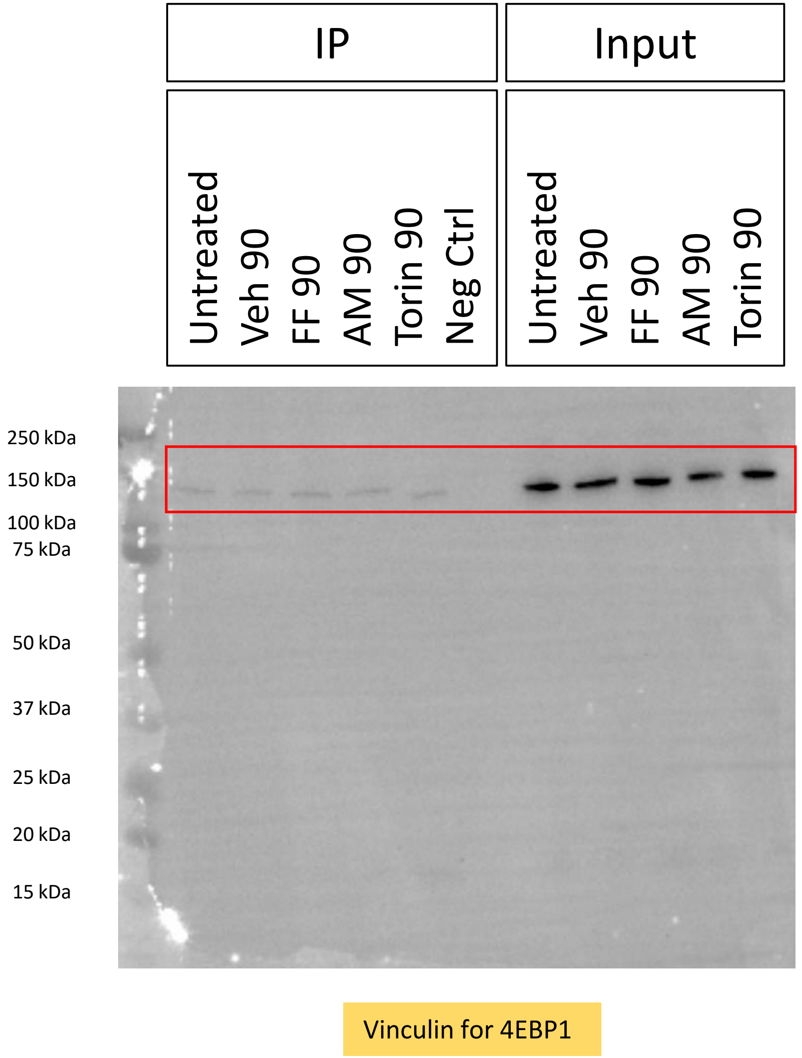
IP
Input
Untreated
Veh 90
FF 90
AM 90
Torin 90
Neg Ctrl
Untreated
Veh 90
FF 90
AM 90
Torin 90
250 kDa
150 kDa
100 kDa
75 kDa
50 kDa
37 kDa
25 kDa
20 kDa
15 kDa
Vinculin for 4EBP1

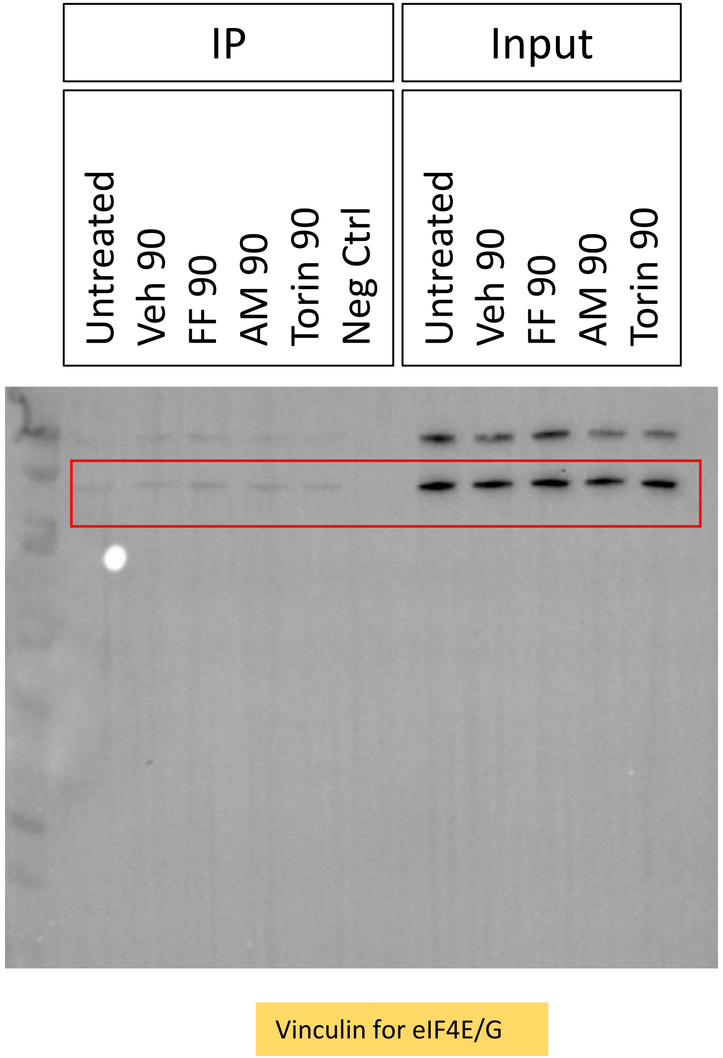
IP
Input
Untreated
Veh 90
FF 90
AM 90
Torin 90
Neg Ctrl
Untreated
Veh 90
FF 90
AM 90
Torin 90
Vinculin for eIF4E/G

6E

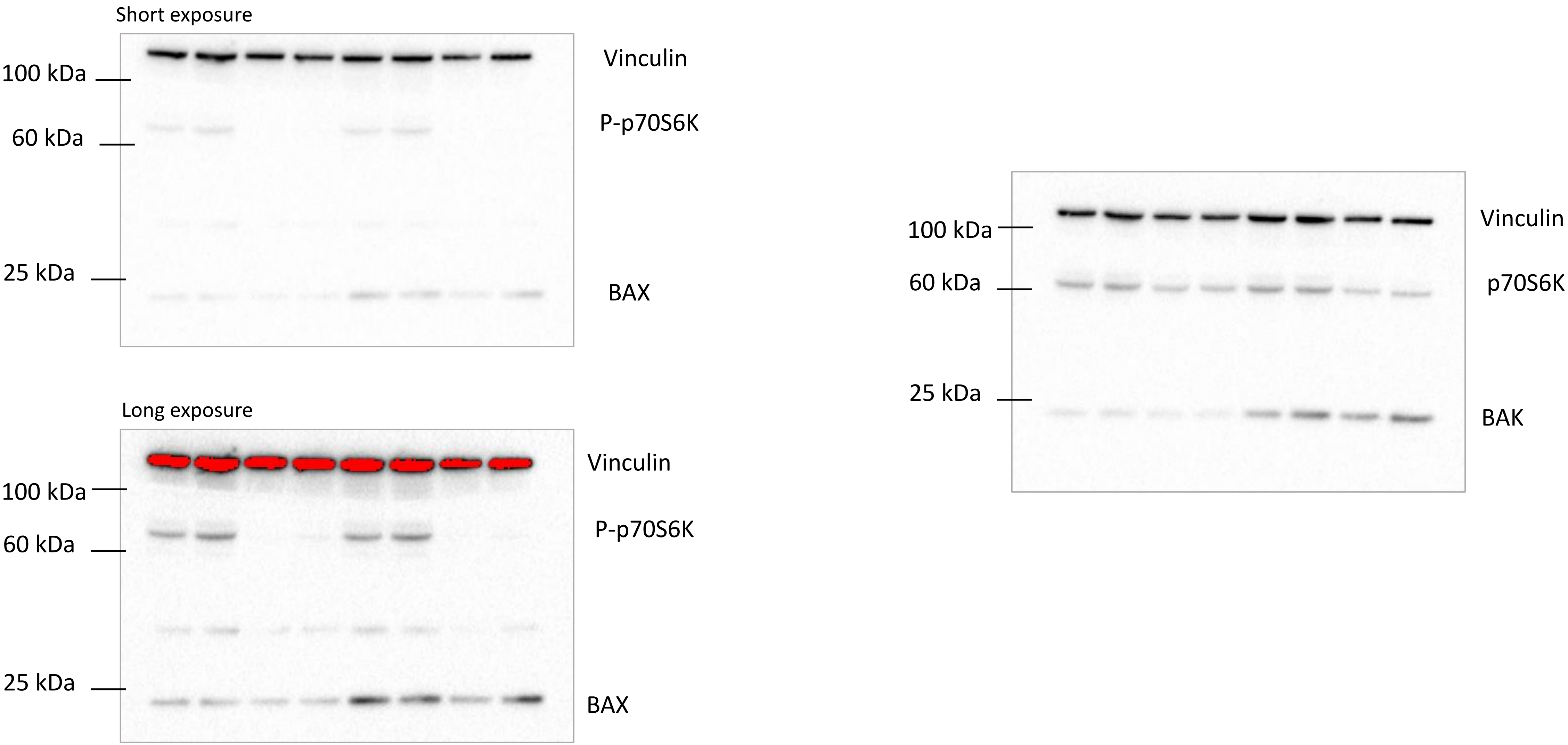
Short exposure
Vinculin
100 kDa
P-p70S6K
60 kDa
25 kDa
BAX
Long exposure
Vinculin
100 kDa
P-p70S6K
60 kDa
25 kDa
BAX
Vinculin
100 kDa
p70S6K
60 kDa
25 kDa
BAK

6F

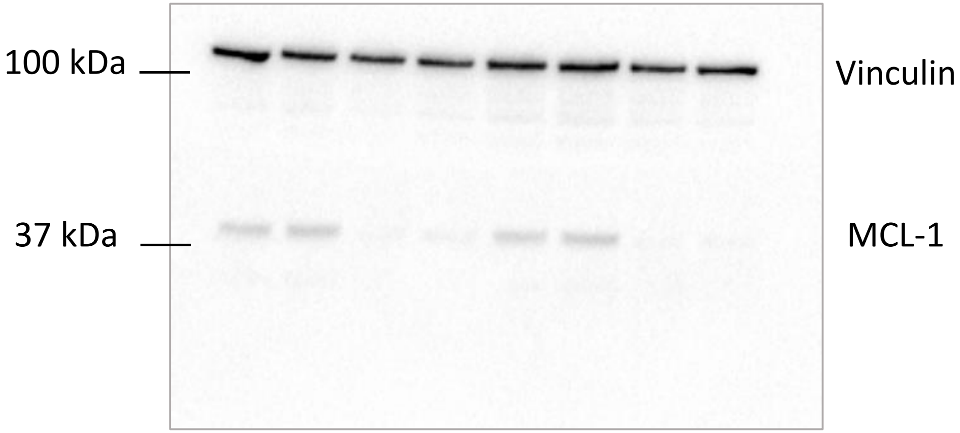

8A

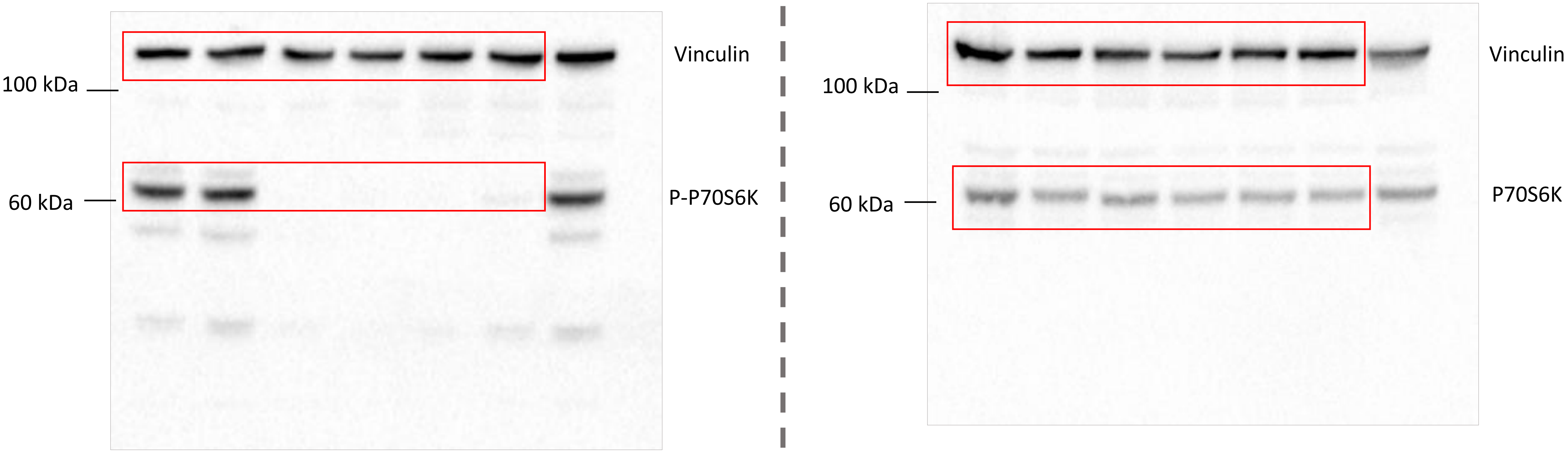

100 kDa
60 kDa
Vinculin
P-P70S6K
100 kDa
60 kDa
Vinculin
P70S6K

8B

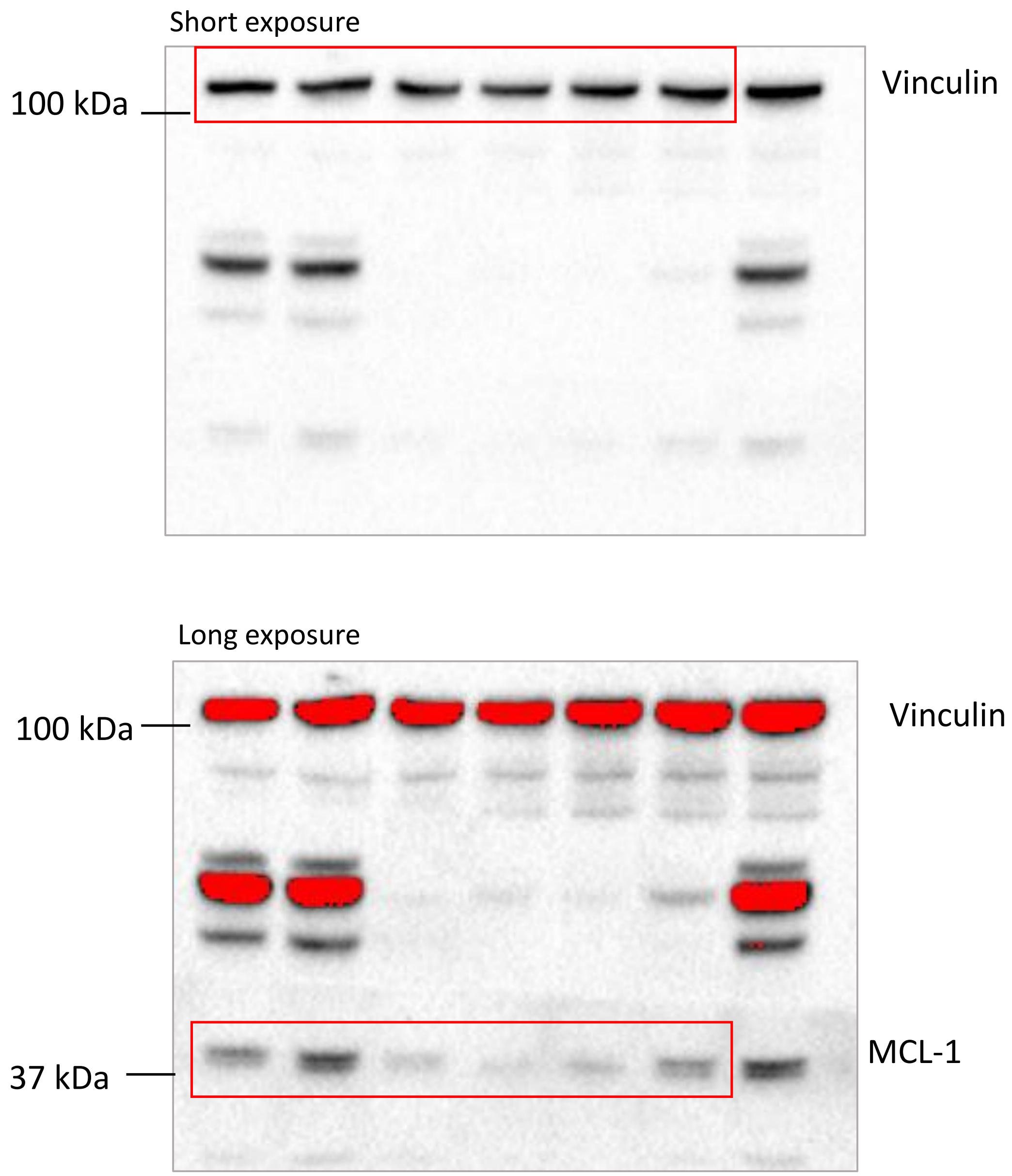
Short exposure
100 kDa
Vinculin
Long exposure
100 kDa
Vinculin
37 kDa
MCL-1

8C

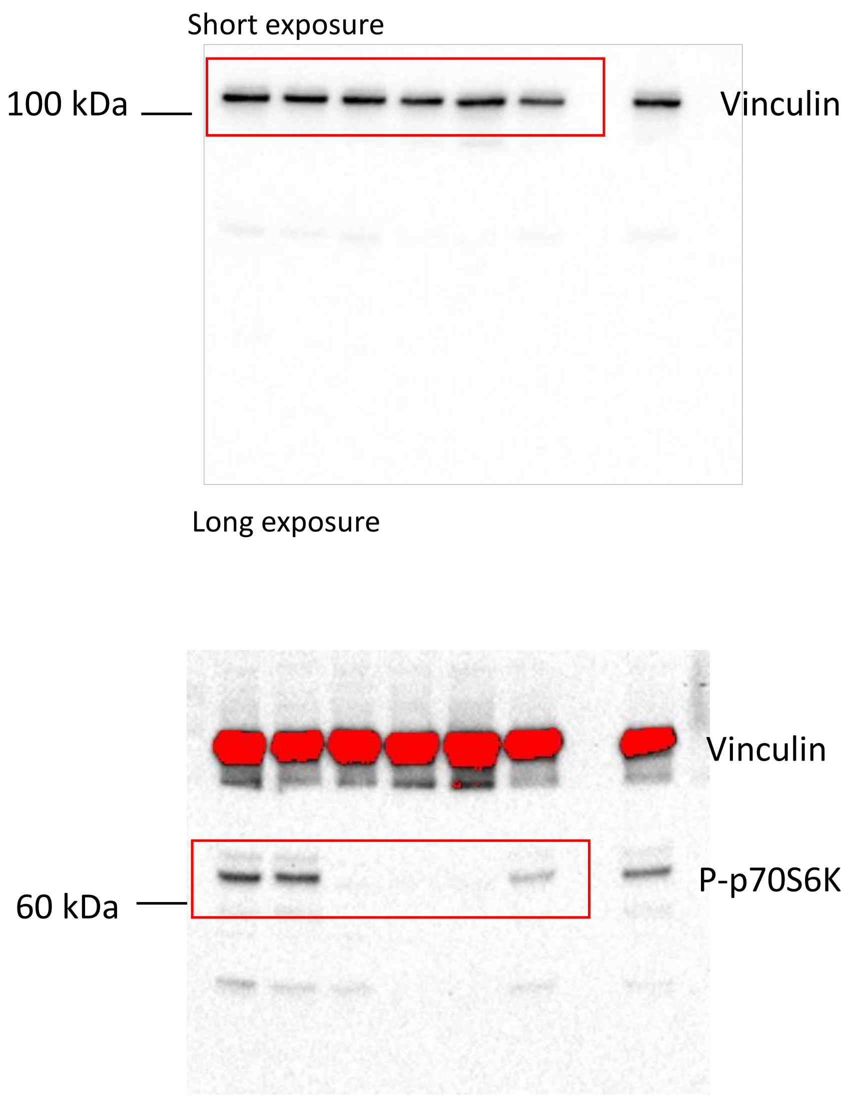

Short exposure
100 kDa
Vinculin
Long exposure
Vinculin
P-p70S6K
60 kDa

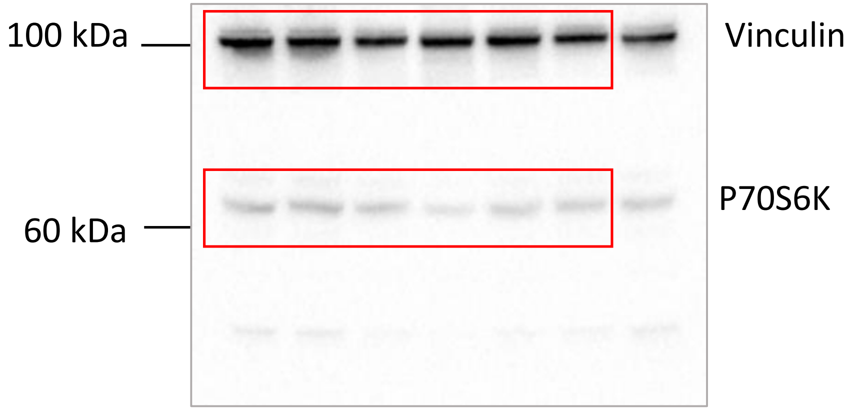

100 kDa
Vinculin
P70S6K
60 kDa

8D

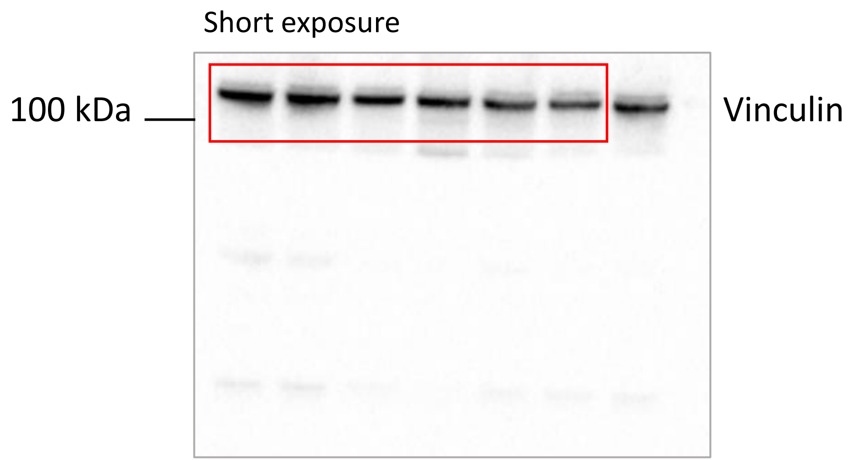
Short exposure
100 kDa
Vinculin

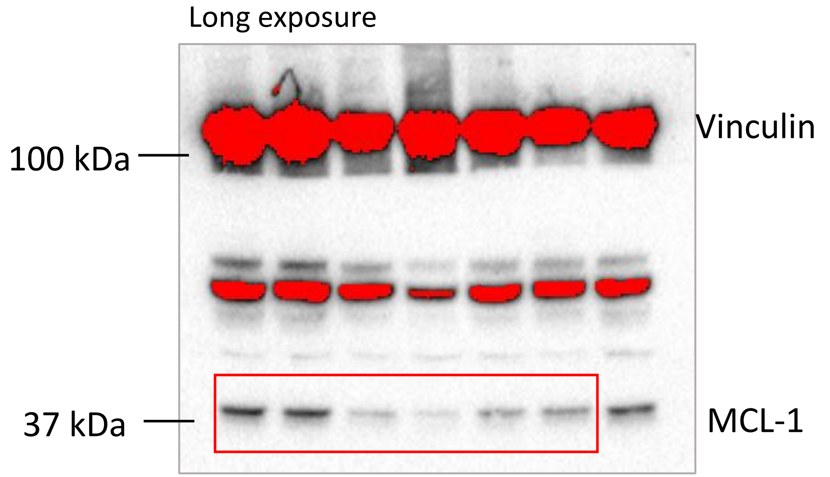
Long exposure
100 kDa
Vinculin
37 kDa
MCL-1

Supplementary Figures

2A

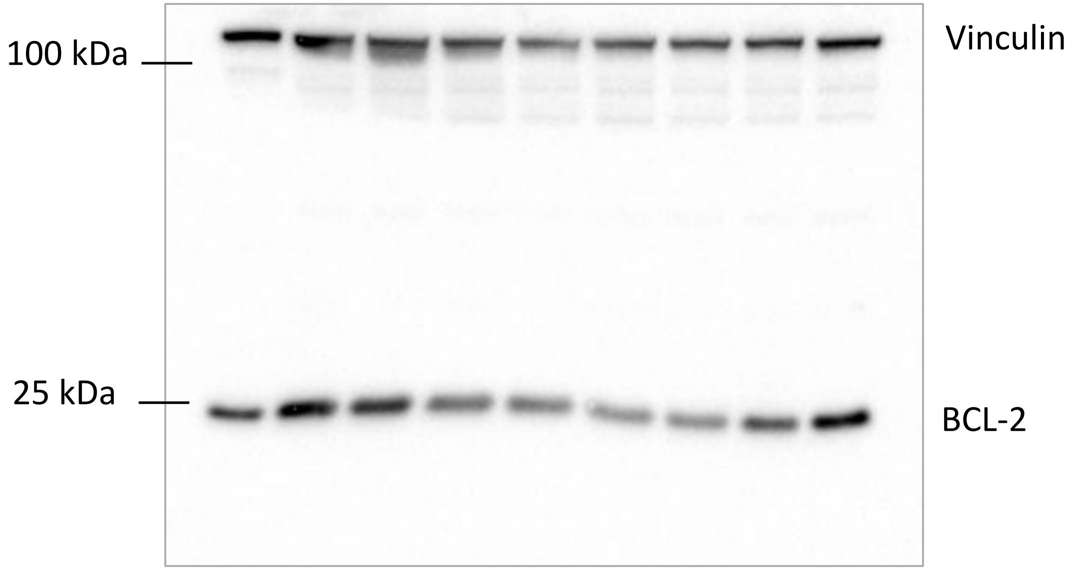

2B

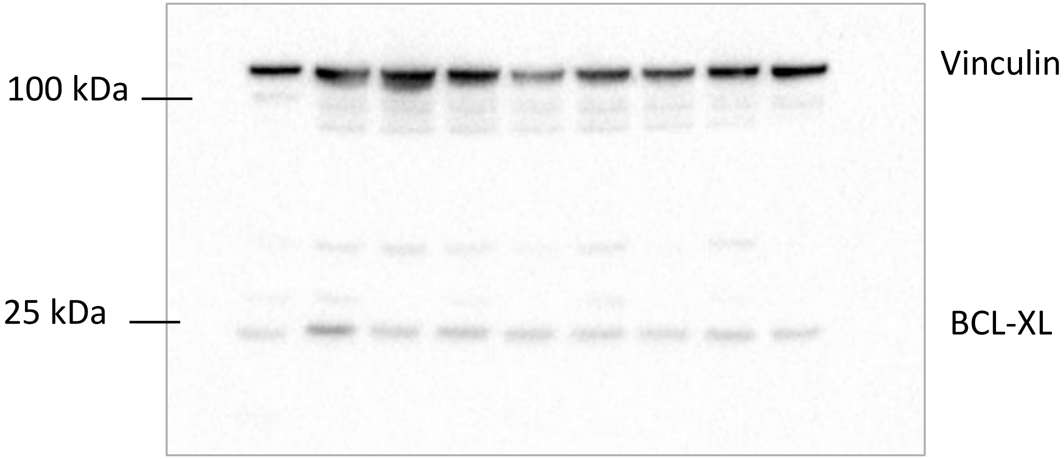

2C

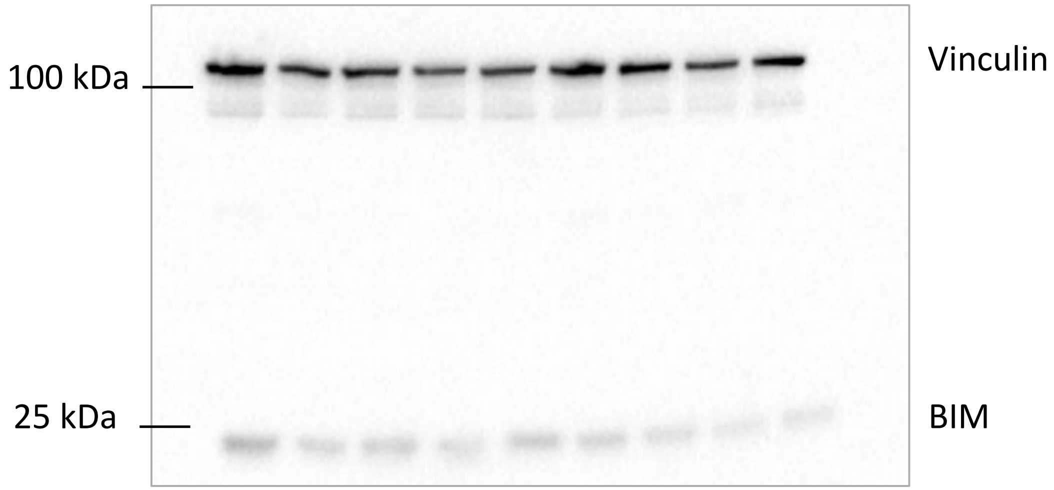

2D

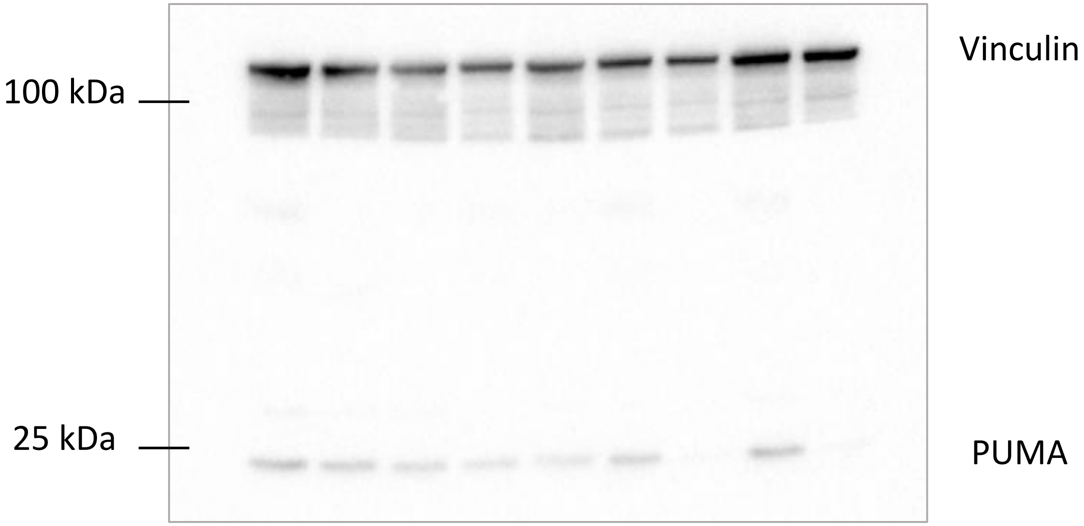

2E

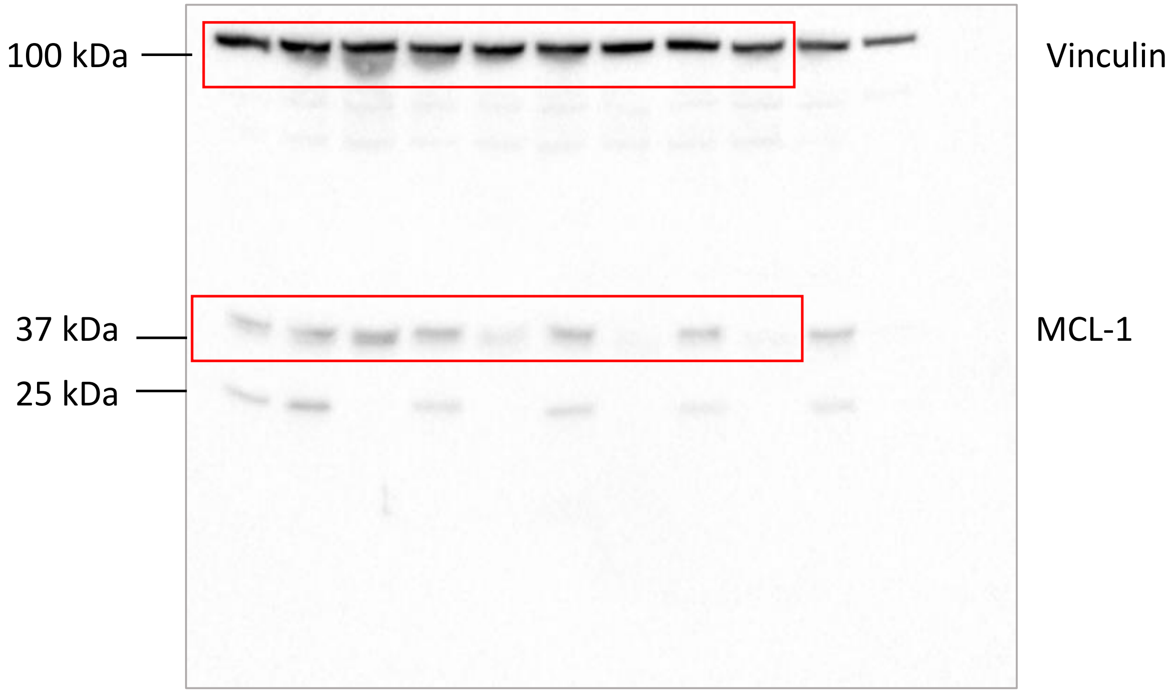

3

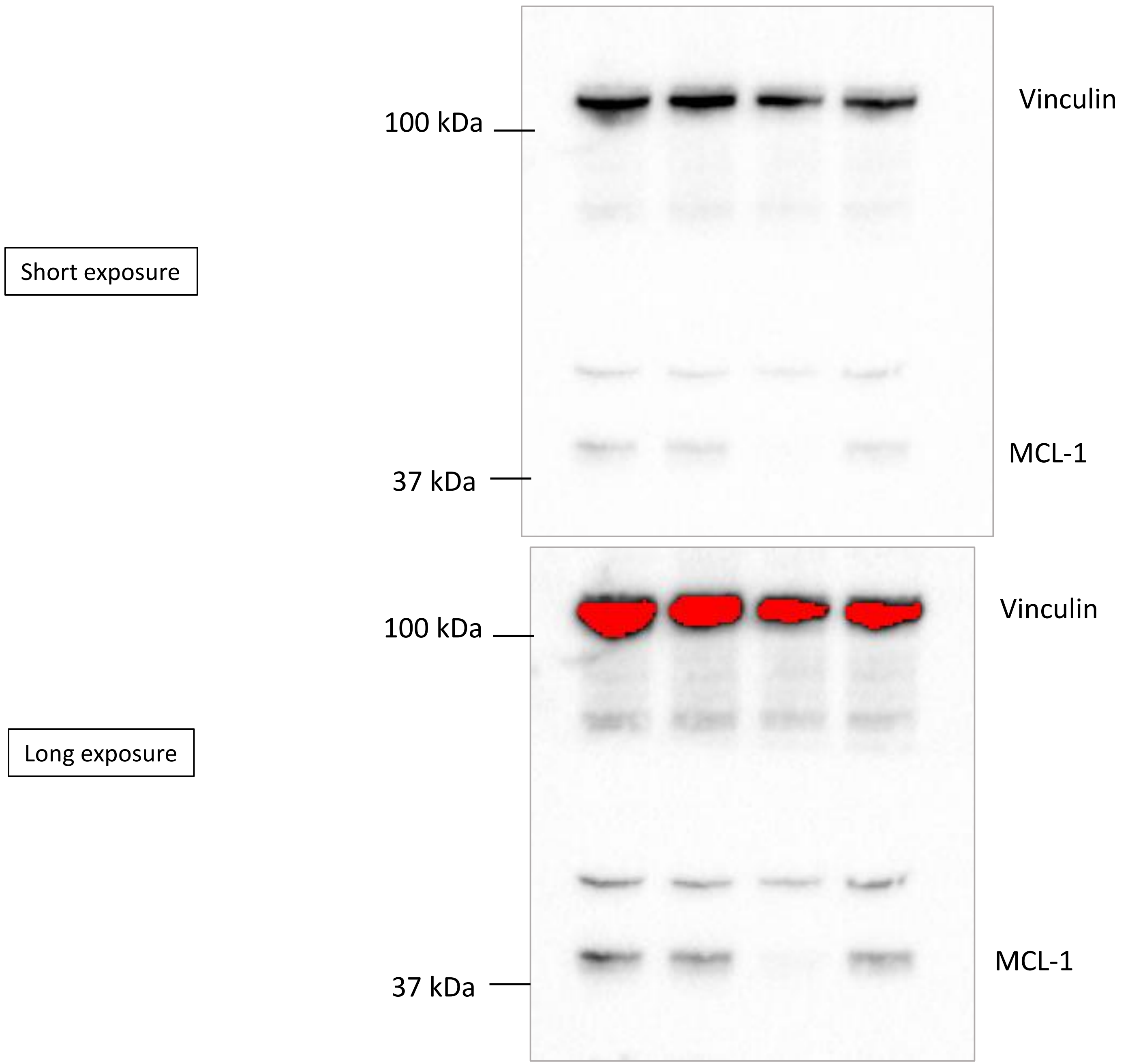
Short exposure
100 kDa
37 kDa
Vinculin
MCL-1
Long exposure
100 kDa
37 kDa
Vinculin
MCL-1

5B

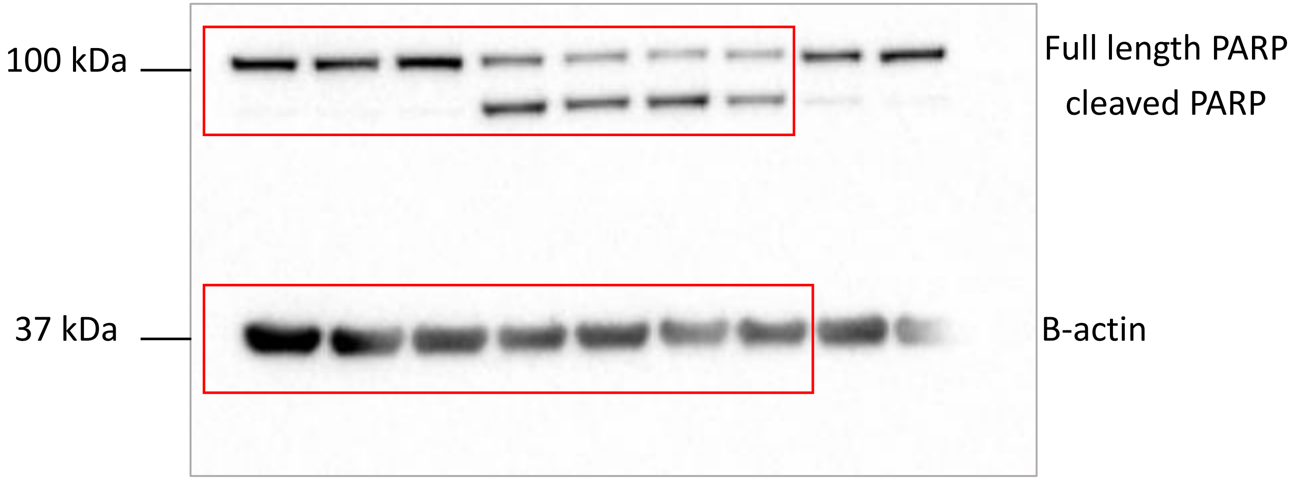

6A

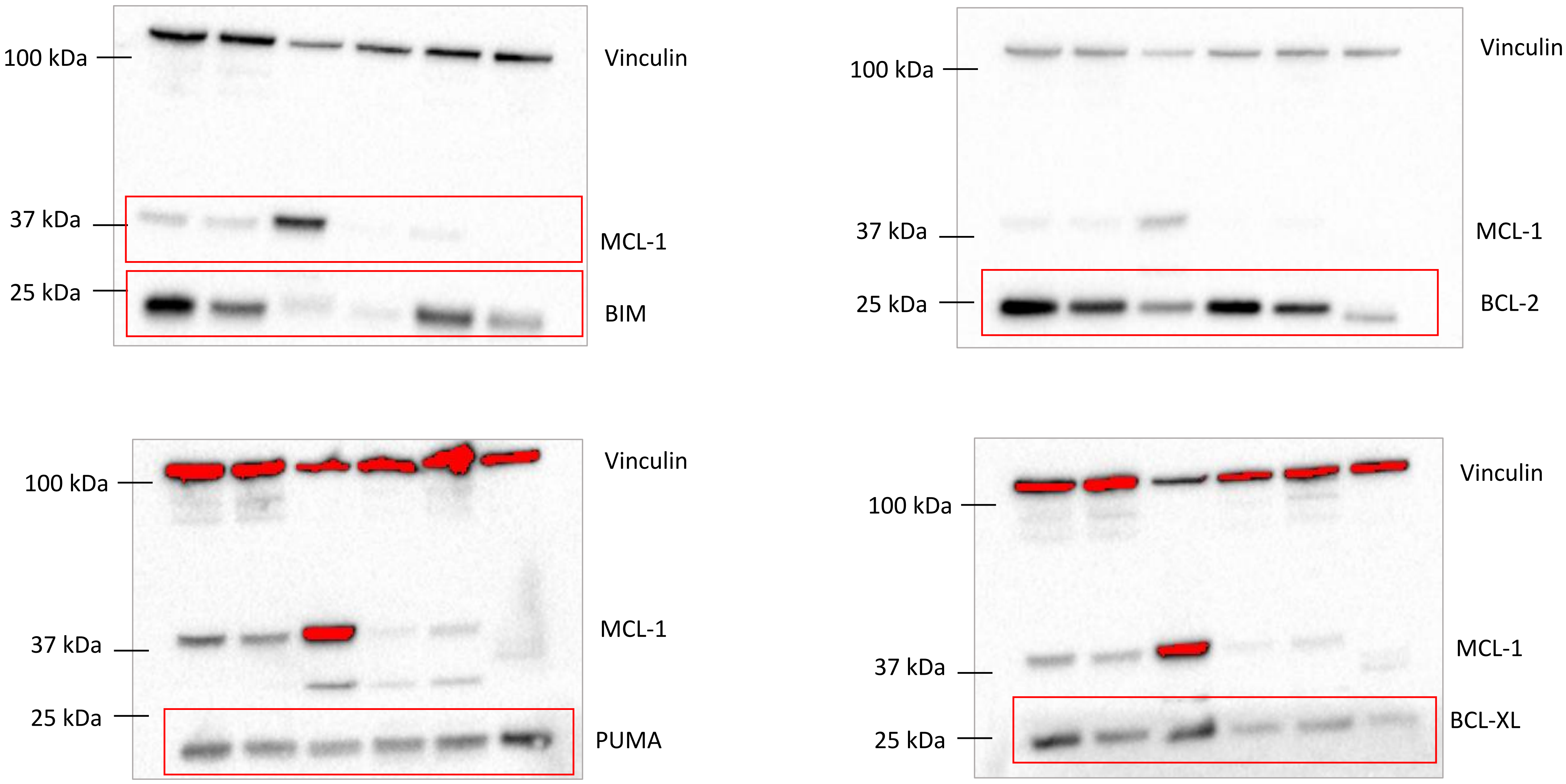
100 kDa
37 kDa
25 kDa
Vinculin
MCL-1
BIM
100 kDa
37 kDa
25 kDa
Vinculin
MCL-1
BCL-2
100 kDa
37 kDa
25 kDa
Vinculin
MCL-1
PUMA
100 kDa
37 kDa
25 kDa
Vinculin
MCL-1
BCL-XL

7A

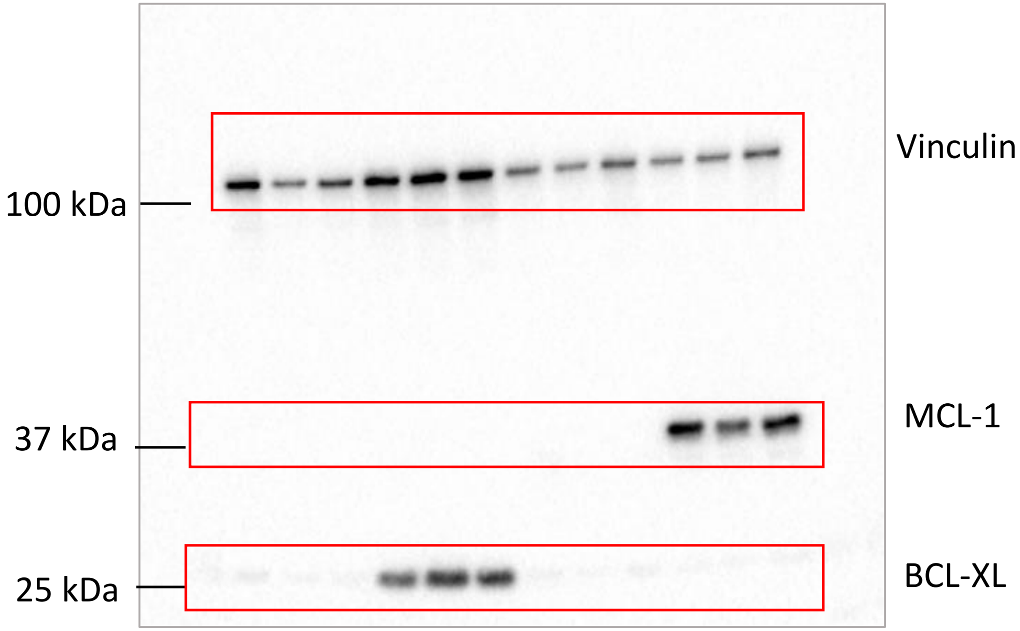

7B

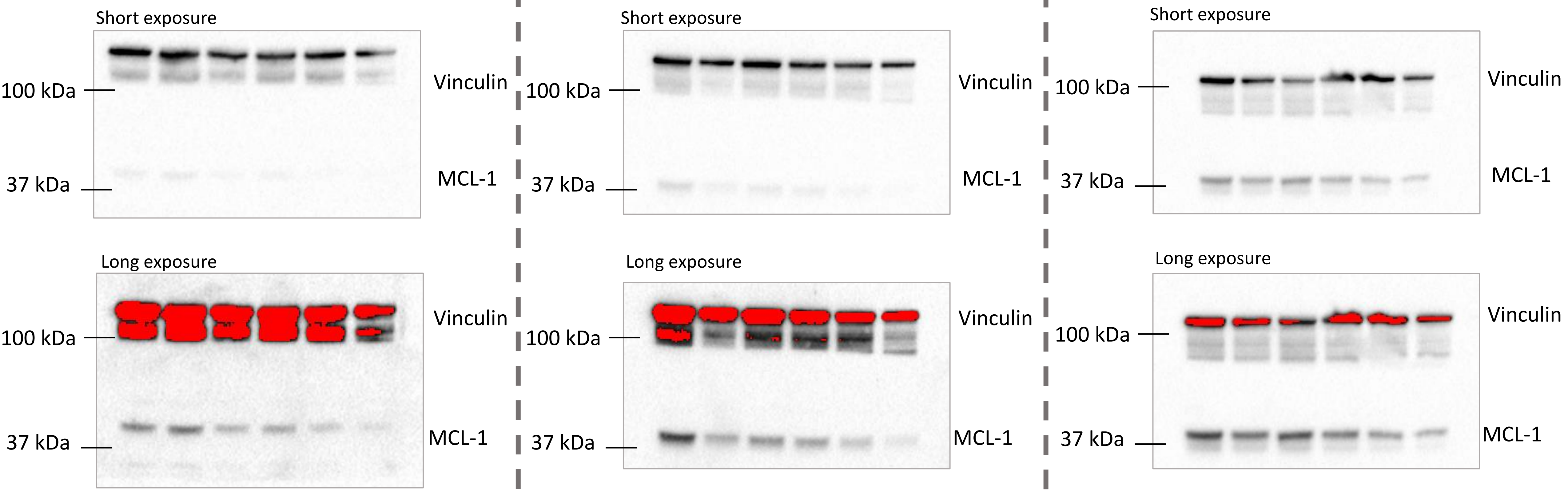

Short exposure
Vinculin
MCL-1
100 kDa
37 kDa
Long exposure
Vinculin
MCL-1
100 kDa
37 kDa
Short exposure
Vinculin
MCL-1
100 kDa
37 kDa
Long exposure
Vinculin
MCL-1
100 kDa
37 kDa
Short exposure
Vinculin
MCL-1
100 kDa
37 kDa
Long exposure
Vinculin
MCL-1
100 kDa
37 kDa

8A

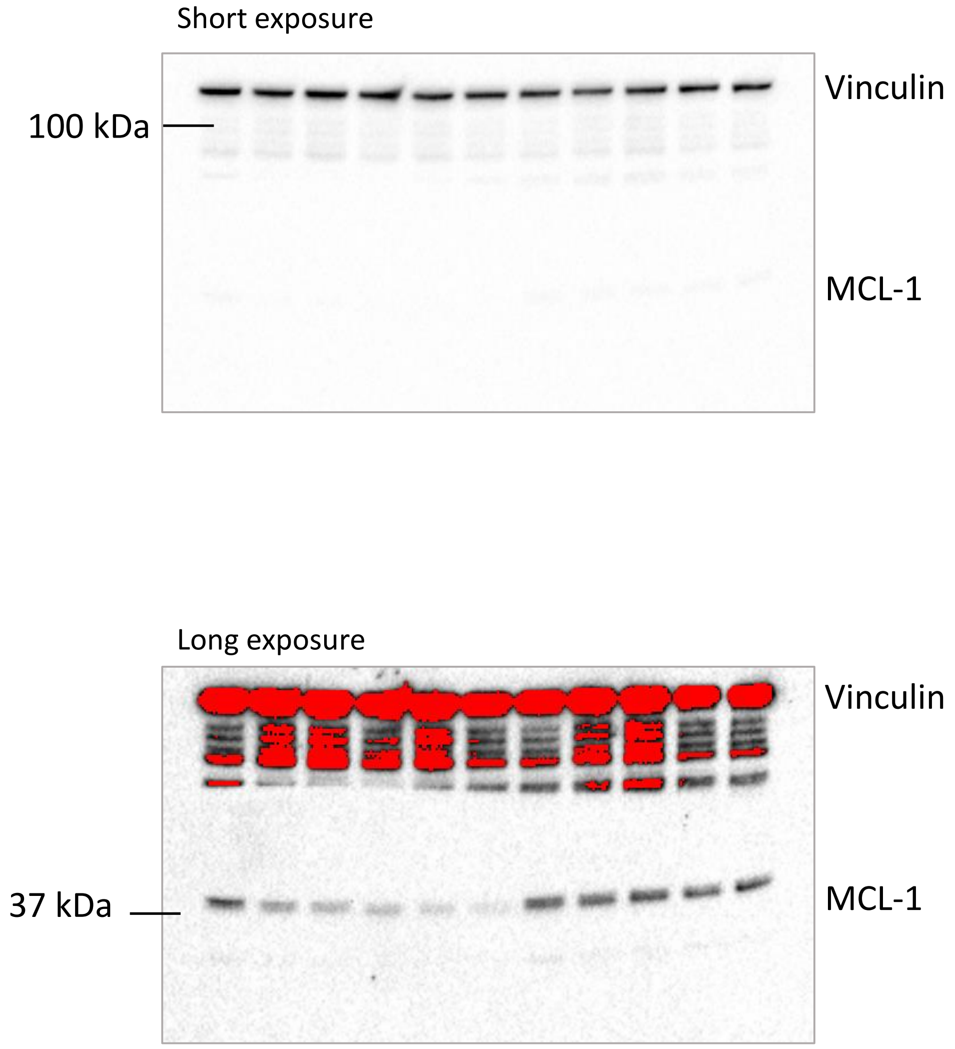

Short exposure
100 kDa
Vinculin
MCL-1
Long exposure
37 kDa
Vinculin
MCL-1

8B

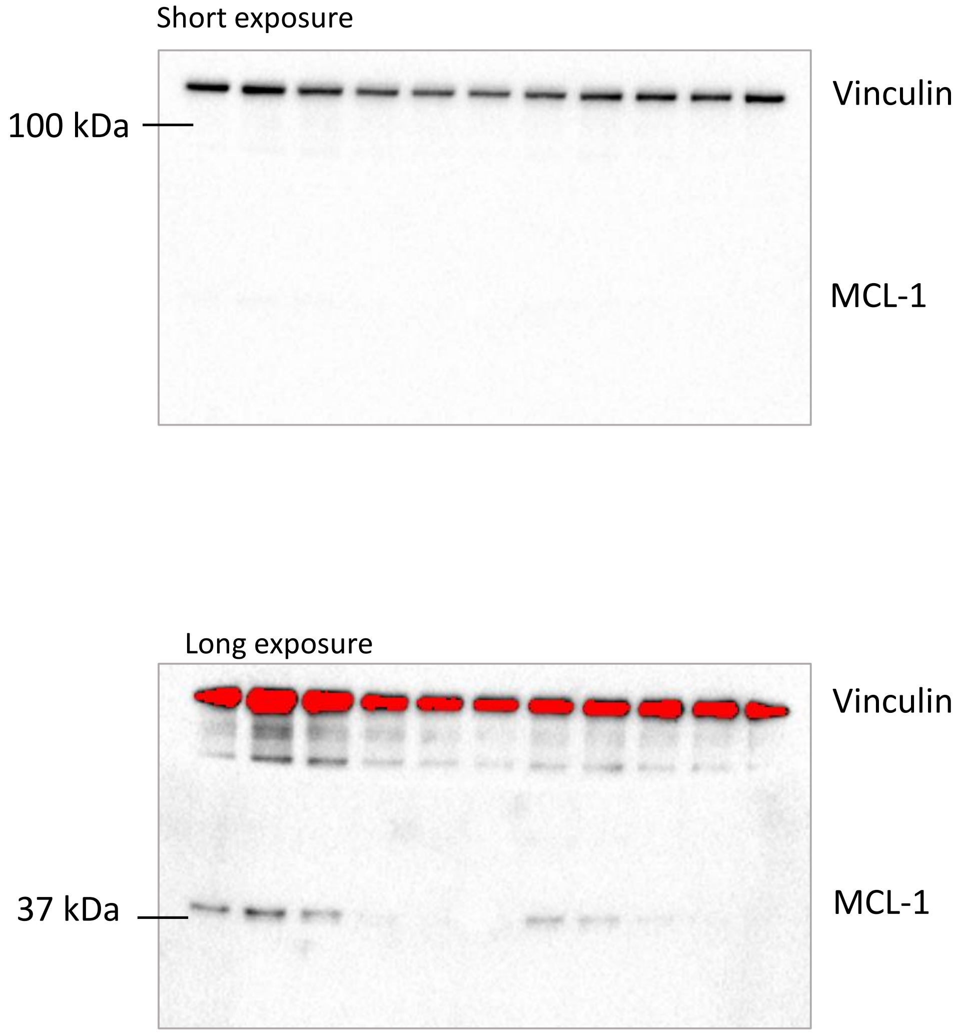

Short exposure
100 kDa
Vinculin
MCL-1
Long exposure
37 kDa
Vinculin
MCL-1

8C

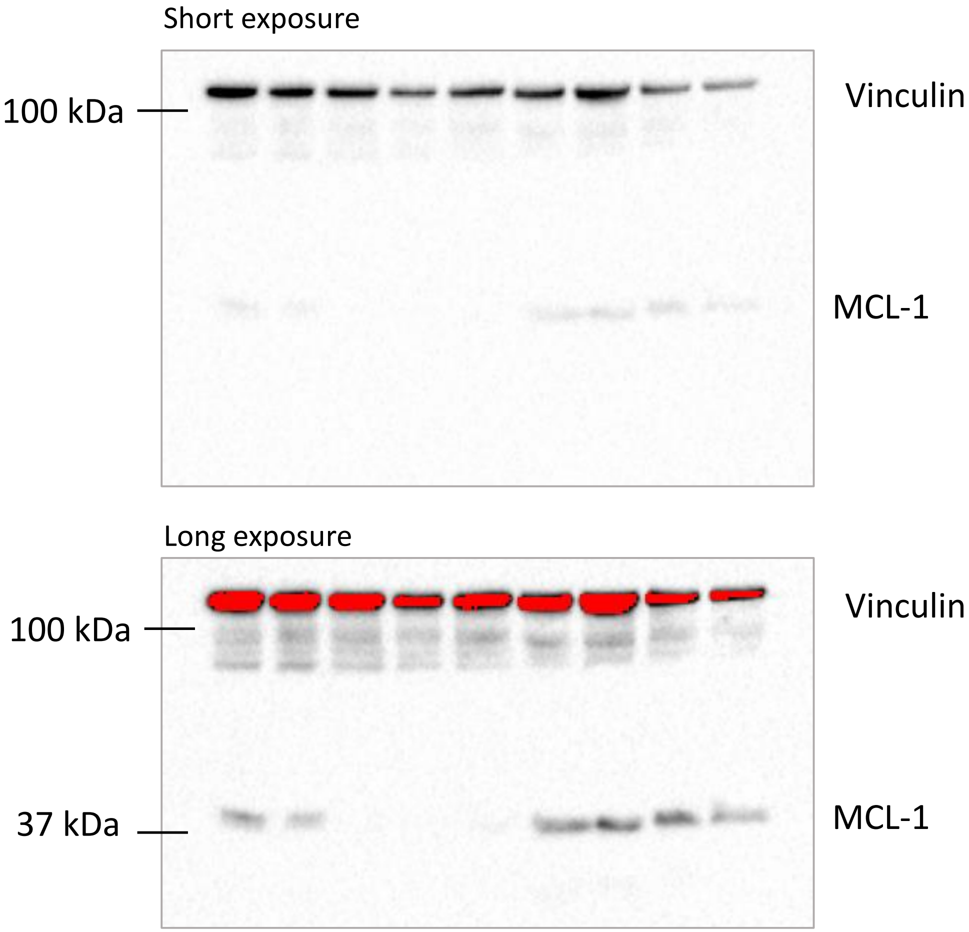
Short exposure
100 kDa
Vinculin
MCL-1
Long exposure
100 kDa
37 kDa
Vinculin
MCL-1

11C

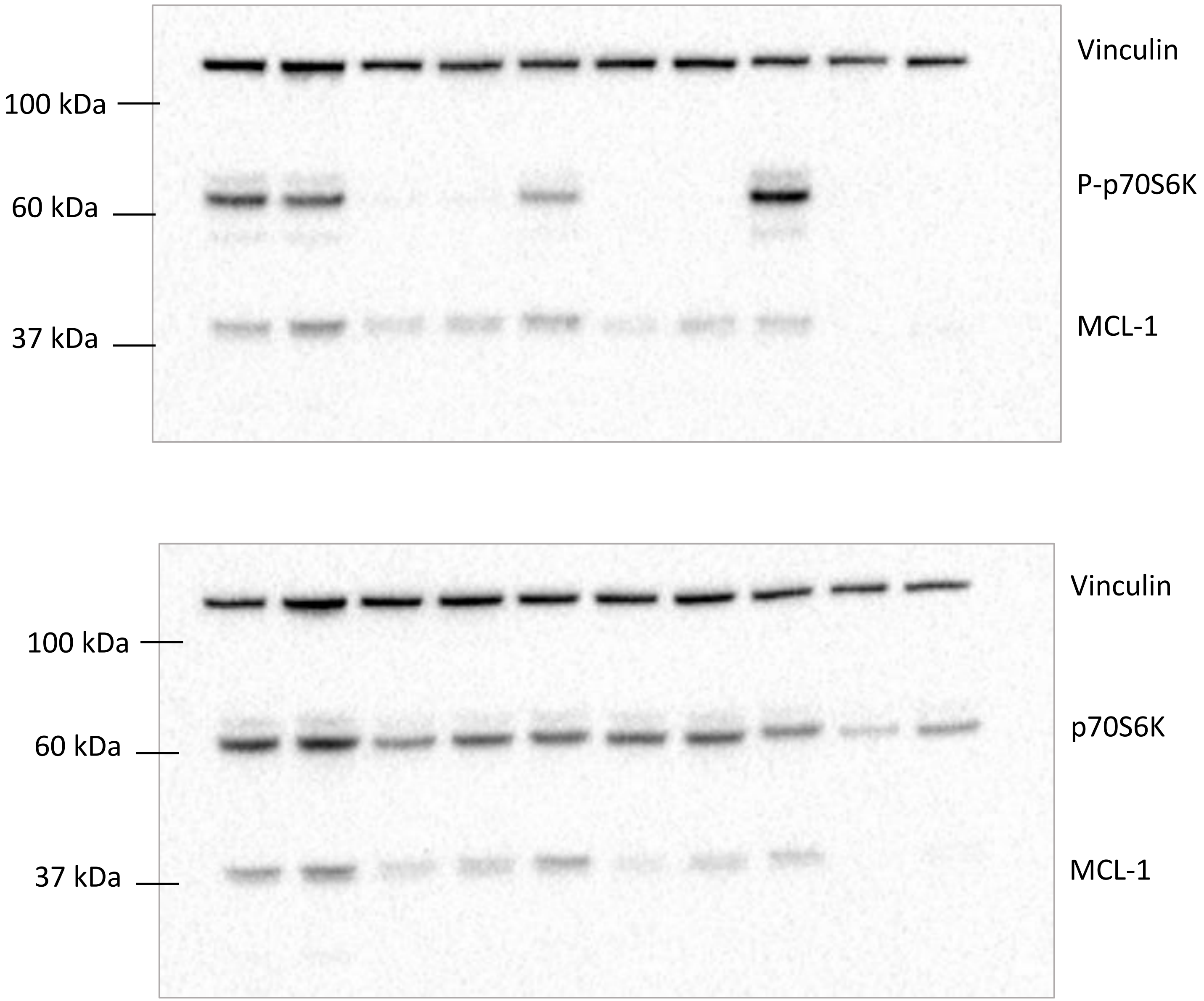
Vinculin
100 kDa
P-p70S6K
60 kDa
MCL-1
37 kDa
Vinculin
100 kDa
p70S6K
60 kDa
MCL-1
37 kDa
